# Mapping mycological ignorance – checklists and diversity patterns of fungi known for West Africa

**DOI:** 10.1186/s43008-020-00034-y

**Published:** 2020-07-07

**Authors:** Meike Piepenbring, Jose G. Maciá-Vicente, Jean Evans I. Codjia, Carola Glatthorn, Paul Kirk, Yalemwork Meswaet, David Minter, Boris Armel Olou, Kai Reschke, Marco Schmidt, Nourou Soulemane Yorou

**Affiliations:** 1 Department of Mycology, Goethe University Frankfurt am Main, Biologicum, Max-von-Laue-Str. 13, 60438 Frankfurt am Main, Germany; 2 Research Unit Tropical Mycology and Plant-Soil Fungi Interactions, Faculty of Agronomy, University of Parakou, BP 123 Parakou, Benin; 3 Key Laboratory for Plant Diversity and Biogeography of East Asia, Kunming Institute of Botany, Chinese Academy of Sciences, Kunming, 650201 China; 4 Royal Botanic Garden, Kew, Richmond, Surrey UK; 5 CABI International, Bakeham Lane, Egham, Surrey TW20 9TY UK; 6 Department of Ecology, University of Kassel, Heinrich-Plett-Str. 40, Kassel, Germany; 7 Senckenberg Biodiversity and Climate Research Centre (SBiK-F), Senckenberganlage 25, 60325 Frankfurt am Main, Germany; 8 Palmengarten der Stadt Frankfurt am Main, Siesmayerstr. 61, 60323 Frankfurt am Main, Germany

**Keywords:** Benin, Countries of West Africa, Fungal diversity, Fungal ecology, History of mycology, Lichens, Phytopathology, No new taxa

## Abstract

Scientific information about biodiversity distribution is indispensable for nature conservation and sustainable management of natural resources. For several groups of animals and plants, such data are available, but for fungi, especially in tropical regions like West Africa, they are mostly missing. Here, information for West African countries about species diversity of fungi and fungus-like organisms (other organisms traditionally studied by mycologists) is compiled from literature and analysed in its historical context for the first time. More than 16,000 records of fungi representing 4843 species and infraspecific taxa were found in 860 publications relating to West Africa. Records from the Global Biodiversity Information Facility (GBIF) database (2395 species), and that of the former International Mycological Institute fungal reference collection (IMI) (2526 species) were also considered. The compilation based on literature is more comprehensive than the GBIF and IMI data, although they include 914 and 679 species names, respectively, which are not present in the checklist based on literature. According to data available in literature, knowledge on fungal richness ranges from 19 species (Guinea Bissau) to 1595 (Sierra Leone). In estimating existing species diversity, richness estimators and the Hawksworth 6:1 fungus to plant species ratio were used. Based on the Hawksworth ratio, known fungal diversity in West Africa represents 11.4% of the expected diversity. For six West African countries, however, known fungal species diversity is less than 2%. Incomplete knowledge of fungal diversity is also evident by species accumulation curves not reaching saturation, by 45.3% of the fungal species in the checklist being cited only once for West Africa, and by 66.5% of the fungal species in the checklist reported only for a single country. The documentation of different systematic groups of fungi is very heterogeneous because historically investigations have been sporadic. Recent opportunistic sampling activities in Benin showed that it is not difficult to find specimens representing new country records. Investigation of fungi in West Africa started just over two centuries ago and it is still in an early pioneer phase. To promote proper exploration, the present checklist is provided as a tool to facilitate fungal identification in this region and to aid conceptualisation and justification of future research projects. Documentation of fungal diversity is urgently needed because natural habitats are being lost on a large scale through altered land use and climate change.

## INTRODUCTION

Scientific data on biodiversity are indispensable for nature conservation and sustainable management of natural resources. Targets 1–3 of the Global Strategy for Plant Conservation (URL 1 2019) provide an example of this and are just as relevant for fungi as for plants. These data are urgently needed to secure the survival of species and natural habitats providing ecosystem services, in the face of pressure from an increasing human population causing land use changes, pollution, and climate change. In West Africa, for large animals like amphibians, birds, fish, mammals, and reptiles, checklists, red lists, and knowledge of the ecology of selected species relevant for their protection are available. To a lesser extent, such data are also available for plants (e.g. Sosef et al. 2017; Schmidt et al. 2012). For fungi and fungus-like organisms (other organisms traditionally studied by mycologists) in West Africa, however, documentation of species diversity started later and was slow because there were only few mycologists. Identification of fungi is a great challenge due to a lack of monographs, reference specimens, and expertise, particularly in the tropics (Piepenbring et al. 2018), and many areas have never been visited by mycologists specifically interested in documenting fungal species diversity (e.g. Hyde and Hawksworth 1997; Hawksworth 2001; Piepenbring et al. 2011a; Rossman et al. 1998). As a result, information about fungal diversity is lacking for most tropical regions. To date, only 91 fungal species have been evaluated for the global Red List established by the International Union for Conservation of Nature, in contrast to more than 70,119 animals and more than 28,000 plants (URL 2 2019). One important reason for this is the very incomplete knowledge of most fungi that is due to their often inconspicuous way of life and the difficulties in identifying them (Willis 2018).

To date, worldwide, approximately 135,000 species of fungi have been described (Kirk 2019b). Total global fungal diversity is, however, undoubtedly much greater. A figure of 1.5 million species (Hawksworth 1991) was, for many years, used as a working estimate. Currently, however, most mycologists believe the number is even greater, with a conservative estimate now placed in the range of 2.2–3.8 million species (Hawksworth and Lücking 2017). Numerous species of fungi are thought to remain undiscovered in tropical regions and biodiversity hotspots (Hawksworth and Lücking 2017). This has been confirmed by diverse studies, for example, for Central America (Bermúdez and Sánchez 2000), for Panama (Piepenbring 2007; Piepenbring et al. 2012), for macrofungi (e.g. Mueller et al. 2007) and for microfungi (e.g. Koukol et al. 2018).

To estimate how many fungal species exist in a given area, the so-called Hawksworth ratio can be used (Hawksworth 1991, 2001). It suggests that in a given area, the ratio of species richness of fungi to species richness of vascular plants is about 6:1. This fungus to plant species ratio has been roughly confirmed by Rudolph et al. (2018) through species inventories using traditional methods. Recent data obtained by environmental sequencing, however, has shown that 6:1 is rather conservative and probably overlooks diversity of fungi revealed by molecular methods (Hawksworth and Lücking 2017; see also discussion).

Checklists on species diversity are fundamental sources of information for the characterization of biodiversity in any given area and recognized as such by the 1992 Rio Convention on Biological Diversity. The need to compile all available records of fungi into a unified database is evident by the following benefits of a checklist.

A checklist of fungal species for a given area:

1. Provides information on the history of mycological activities and thereby helps to understand the present state of knowledge of fungi in the area.
2. Is indispensable for the acquisition of species knowledge and knowledge on species is indispensable to understand ecological processes (“Kein Ökologieverständnis ohne Organismenkenntnis”, transl. “No understanding of ecology without knowledge on organisms”, Oberwinkler 2012).
3. Helps to identify fungi collected in the area by providing possible names of species and references to literature for identification. Information about associated organisms is important for identification, for example host plants (species, families) for plant pathogenic fungi.
4. Provides forgotten names that may be revived by new collections.
5. Is indispensable to decide whether a record of a fungal species is new for the area.
6. Helps to identify undersampled taxonomic or ecological groups as well as poorly explored geographical areas; yields arguments to justify research projects.
7. Provides numbers for the comparison of biodiversity among regions/countries/continents and along gradients (biogeography).
8. Yields ecological and distributional data for environmental management, exploitation of natural resources, the discussion of conservation strategies, and decision taking.
9. Is essential in monitoring movements of species in response to environmental factors, for example climate change, or as potential invasives.

For vascular plants in Africa, many publications have drawn attention to the lack of species diversity information and georeferenced data (e.g. Küper et al. 2006). Nevertheless, there are published accounts (floras) of the plants of most West African countries including keys and species descriptions, as well as plant checklists and databases (e.g. Daget 2012; Schmidt et al. 2012) available for phytogeographic analyses (e.g. Klopper et al. 2007). For fungi, however, there are no published accounts including species descriptions for any West African country, and checklists of fungi, macrofungi, or plant pathogenic fungi are available for only a few countries, e.g. Ghana (Dade 1940; Hughes 1952, 1953; Piening 1962), Senegal (Kane and Courtecuisse 2013), and Sierra Leone (Deighton 1936a, 1936b, 1956).

The goal of the present study is to gather and analyse the information available for fungal species diversity for West Africa and West African countries based on records available in scientific publications. Specifically, it is to: (1) assess and evaluate the current knowledge of fungal species richness, systematic positions, and ecology in West Africa and West African countries; (2) assess the potential of databases (e.g. GBIF, IMI fungarium database) for contributing further records; (3) explain the present situation in the historical context of mycological research; and (4) contribute new records of fungi for Benin and West Africa to exemplify the current lack of mycological knowledge. By compiling this checklist, we are making available a tool for the identification of fungi in West Africa and for development of informed strategies to promote mycology and fungal conservation in West African countries.

## METHODS

### Geographic focus and plant diversity in West Africa

**Fig. 1:**
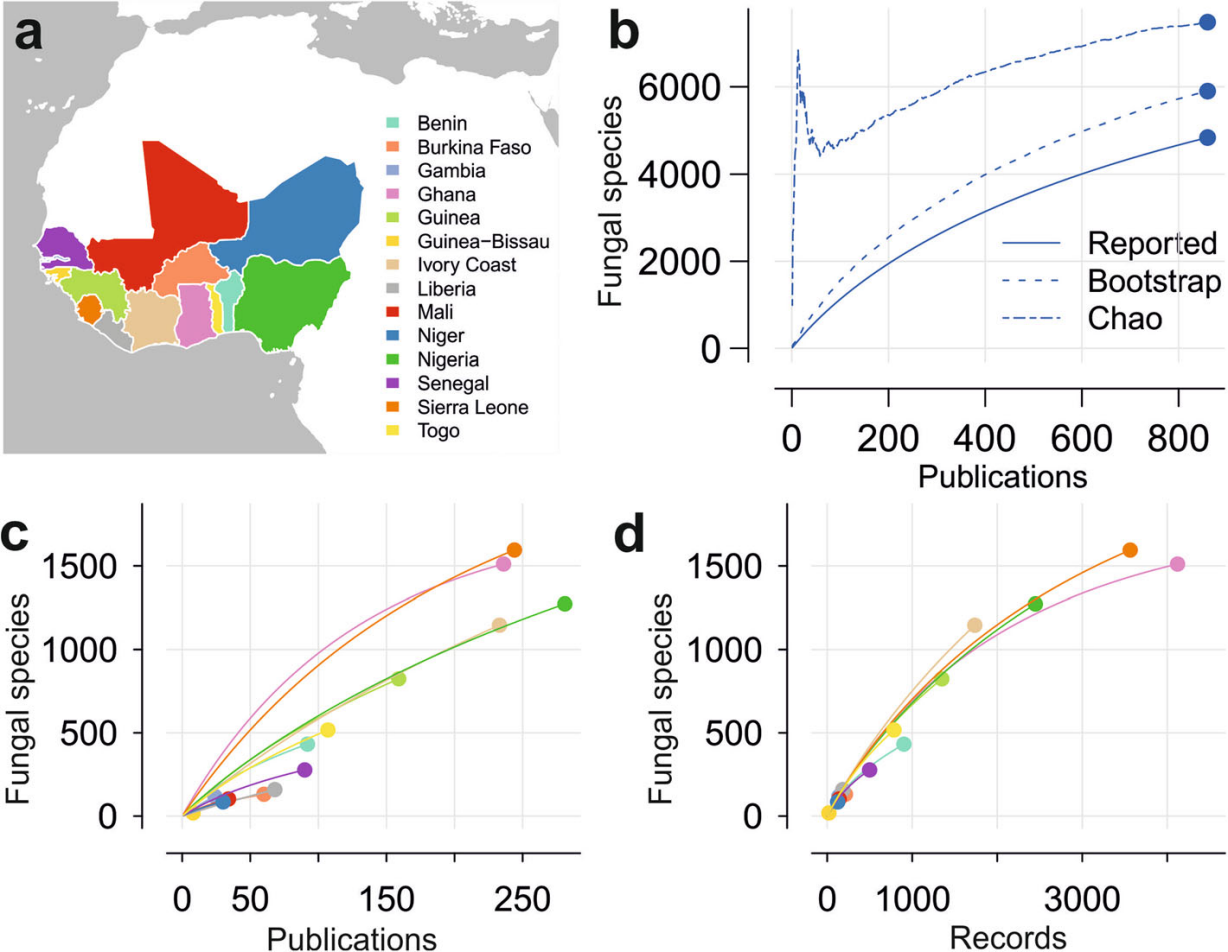
Numbers of fungal species and estimations for fungal species richness in West Africa and West African countries. **a** Countries of West Africa considered for the present analysis. **b** Accumulation curve of numbers of fungal species known for West Africa reported by publications. Dashed lines show the accumulation of species as calculated with the richness estimators Bootstrap or Chao. **c** Accumulation curves of numbers of fungal species known for West African countries based on increasing numbers of publications that were analysed. Colours indicate the countries as shown in Fig. 1a. **d** Accumulation curves of numbers of fungal species known for West African countries based on increasing numbers of records. Colours indicate the countries as shown in Fig. 1a

West Africa is considered here as the geographic region formed by the regions of 14 West African, subsaharan, tropical, continental countries (Fig. 1a, Table 1).

**Table 1:** Current (and historical) names and surface areas of West African countries, known species diversity of plants according to literature, and known and estimated species diversity of fungi according to data in the checklist for fungi in West Africa

							Estimated species richness of fungi			
	Surface (km^**2**^)	Plant species (n)	Reference for plant species	Fungal records (n)	Fungal species (n)	Publications (n)	Chao^a^	Bootstrap^a^	Hawks-worth’s^b^	Known richness (%)^c^
**WEST AFRICA**	5,983,016	7072	A	16,222	4843	860	7485 ± 144	5900 ± 151	42,432	11.4
**Benin** (Dahomey)	117,034	2807	B	902	432	92	923 ± 85	543 ± 31	16,842	2.6
**Burkina Faso** (Upper Volta)	274,748	2080	C, D	214	131	60	225 ± 28	166 ± 19	12,480	1.0
**Gambia**	10,354	1760^d^	E	137	118	24	481 ± 122	157 ± 21	10,560	1.1
**Ghana** (Gold Coast)	240,496	2971	F	4121	1511	236	1798 ± 33	1785 ± 175	17,826	8.5
**Guinea** (French Guinea)	278,060	2923	G	1348	824	159	1957 ± 138	1056 ± 68	17,538	4.7
**Guinea-Bissau** (Portuguese G.)	32,182	1507	H	20	19	8	161 ± 157	25 ± 5	9042	0.2
**Ivory Coast**	323,544	3853	I, J	1736	1146	233	2958 ± 184	1488 ± 89	23,118	5.0
**Liberia**	96,024	2403	F	184	159	68	999 ± 282	213 ± 24	14,418	1.1
**Mali** (French Sudan)	1,263,106	1739	K	140	103	34	258 ± 52	134 ± 14	10,434	1.0
**Niger**	2,104,762	1218	L, M	123	85	30	169 ± 30	109 ± 13	7308	1.2
**Nigeria**	915,098	3378	F	2449	1273	281	2376 ± 106	1613 ± 69	20,268	6.3
**Senegal**	198,094	2300	N	499	277	90	511 ± 48	350 ± 28	13,800	2.0
**Sierra Leone**	72,154	1883^e^	F	3564	1595^e^	244	2307 ± 66	1966 ± 102	11,298	14.1
**Togo**	57,362	3134	O	785	517	107	1310 ± 123	665 ± 64	18,804	2.7

References for plant species:

A, Hepper (1972); B, Akoegninou et al. (2006); C, Thiombiano et al. (2012); D, Schmidt (2018); E, Jones (1994); F, Sosef et al. (2017); G, Lisowski (2009a, 2009b); H, Catarino et al. (2008); I, Aké Assi (2001); J, Aké Assi (2002); K, Boudet et al. (1986) cited by Zizka et al. (2015); L, Lebrun et al. (1983); M, Lebrun et al. (1996); N, Berhaut (1967) cited by Jones (1994); O, Fousseni et al. (2014)

^a^ Mean ± standard error of estimated species

^b^ Hawksworth’s index is calculated based on the ratio of 6 fungal species on all substrates:one vascular plant species (Hawksworth 1991)

^c^ Percentage of species known in a given area based the Hawksworth’s ratio

^d^ The only information available for species diversity of plants in Gambia was found in Jones (1994), who established that his selection of 160 species of flowering plants represent 10–12% of the total number of flowering plants in Gambia. We used the value of 11% to calculate a tentative number of known vascular plants for this country

^e^ Deighton (1903–1992), who worked as a botanist and mycologist in Sierra Leone for many years before being based at IMI, had a typescript checklist that he was continually updating long after his retirement. This typescript checklist was never published and has not been re-found (D.L. Hawksworth, pers. comm.). In 1990 this evidently included some 2050 fungal species as he then gave the number of plant species as about 4100 and the fungus:plant ratio as 0.5:1 (Hawksworth 1991). These figures have not been used in these calculations as in the absence of his manuscript the records could not be updated to current species concepts for plants or fungi

Country names and borders have changed over time. From the end of the fourteenth century until the end of the nineteenth century, the entire West African area adjacent to the gulf of Guinea, and even as far south as Angola, was referred to by Europeans as the Coast of Guinea, so records for Guinea from this time are particularly difficult to interpret. Several countries adopted names of precolonial kingdoms, not necessarily from the same place, like the country of Benin, which has the same name as the former kingdom of Benin which was located in what is now Nigeria. Borders also changed in colonial times: between 1932 and 1947, for example, Upper Volta was divided among the territories of French Sudan (Mali), Ivory Coast, and Niger. The German colonies of Cameroon and Togoland were divided after World War I between the British and French, with the British part of Togoland now belonging to Ghana and the northern part of British Cameroon now being part of Nigeria.

Most of the area of West Africa is dominated by extensive plains that are only locally interrupted by mountain chains, high plateaux, or inselbergs (Porembski 2000). With few exceptions, topodiversity is relatively low (Mutke et al. 2001) and, accordingly, vegetation is rather homogenous with relatively low levels of plant endemism (e.g. for Ivory Coast: Aké Assi 2002, for Togo: Brunel et al. 1984). Vegetation is influenced by a steep rainfall gradient that increases from the Sahara desert towards the coastal rainforests in the south, expressed in a series of vegetation zones forming parallel bands in an East-West direction throughout West Africa (Barthlott et al. 2007; Da et al. 2018; Hahn-Hadjali et al. 2010; White 1983; Linder et al. 2012). At the southern border of the Sahara desert lies the Sahel, with dry savannas of thornbushes and low grasses. The Sudanian Zone is characterized by tall-grass savannas with a denser, richer tree layer and frequent fires. In its southern part, *Isoberlinia* dominated woodlands prevail, similar to the southern African miombo. The Guinean Zone was covered mainly by rainforests, but has now mostly been converted to moist secondary savannas. In the southern parts of Benin, Togo, and up to the Accra plain (Ghana), the coastal rainforest zone is interrupted by savannas, known as the ‘Dahomey gap’ (Salzmann and Hoelzmann 2005).

Numbers for species richness of vascular plants known for West African countries were retrieved from literature and compiled in Table 1. When different numbers for vascular plants are available for a given country, the higher number is cited in Table 1 and used for further analyses. More vascular plant species are probably known, particularly for Ghana, Liberia, and Sierra Leone, because Sosef et al. (2017) only used the records available from the RAINBIO-database, but higher numbers for these countries in recent literature were not found during the present study.

Plant species richness is relatively low in West Africa compared with other parts of Africa (Barthlott et al. 2007). Hepper (1972) cites a total number of 7072 species of angiosperms, gymnosperms, and pteridophytes in his quantitative analysis of the *Flora of West Tropical Africa*.

### Data considered for the checklist of fungi

The checklist of fungi known for West Africa is based on primary literature (scientific publications in international journals with peer review process, books with ISBN number) and secondary literature (review papers, lists). These publications were found by searches, including Google Scholar, Cybertruffle (Minter 2020), the database of the US Department of Agriculture (Farr and Rossman 2019), and using references included in Lindau and Sydow (1915), Hawksworth and Ahti (1990), and the analysed publications. A list of species that were described from West African countries was obtained from data compiled in Index Fungorum (Kirk 2019a). So-called ‘grey literature’, i.e. documents produced outside of the traditional academic publishing and distribution channels, unpublished theses, databases, and “private” publications were not included. The checklist (Additional files 1, 2) contains information for records of fungi for West African countries in an excel file, including taxonomy, systematic positions, research contexts, life forms, substrata, associated organisms, and references to literature (for references, see Additional file 3). Data included in the checklist based on literature is publicly available via the PANGAEA portal (URL 4 2019) and by the MyCoPortal online database (MyCoPortal 2019).

### Data analyses

Data analyses were performed with R version 3.6.0 (R Core Team 2013). For the analyses, species and infraspecific taxa were considered, while records limited to genus level in the literature (e.g. *Russula* sp.) were not considered. Records with names that were not validly published or that could not be found in Index Fungorum (Kirk 2019a) were included because most probably they refer to distinct species, whereas synonyms are not included in the analyses.

We compared the species records in the checklist based on literature with those for West Africa on the website of the Global Biodiversity Information Facility (GBIF, URL 3 2019) and in the database of the former International Mycological Institute (IMI) fungarium, using an edited version of records produced up to May 1989, not yet available on-line (D. Minter, pers. comm.). For these comparisons, we first normalized the species names in all datasets by comparing and where appropriate substituting them with accepted species names from the Species Fungorum (SF) and Index Fungorum (IF) database, so that disparities between the records represented genuinely different taxa rather than mere nomenclatural differences. In addition, before comparisons, records in all three datasets that were identified only above the rank of species (e.g. genus) were dropped, and those with intra-specific identifications were replaced by species-level names. Species names in our checklist were compared with both the GBIF and IMI databases, and the species which were unique or shared were recorded, both for entire West Africa as a whole and for every West African country individually.

Numerical and graphical summaries for data obtained from literature were obtained for West Africa or for individual West African countries via custom functions and plots. The R package *maps* version 3.3.0 (Becker et al. 2018) was used to draw maps of West Africa and to extract geographical information about the countries studied, such as their centroid coordinates and total areas. The sources of this information were the maps of the Natural Earth project (URL 5 2019). Functions in the package *vegan* version 2.5–4 (Oksanen et al. 2007, 2019) were used to calculate species richness as well as the richness estimators Bootstrap and Chao (Magurran and McGill 2011), and to build curves of species accumulation with sampling coverage, based on records or on the number of publications screened. The similarity in species composition across countries as well as potential correlations of species similarity with geographical distance (using countries’ geographical centroids for each country) were evaluated using the Mantel test and Mantel correlograms based on the Jaccard index (Legendre and Legendre 2012), using functions available in *vegan*.

For the analysis of the geographic origin of first authors of publications containing records of fungi in West African countries, first author names were classified as African origin / European origin / rest of the world. This information was deduced mainly from names and institutional affiliations, and may contain errors.

All data and R code necessary to reproduce the analyses have been made available online at Figshare (URL 6 2020).

### Case study: results of mycological fieldwork in Benin

Our interest in the fungal diversity of Benin and neighbouring countries stemmed from several Summer Schools in Benin for African and European mycologists carried out in Benin, each held over 3 weeks. The Summer Schools carried out in July/August in 2016 and 2017 are relevant for the present study. In the context of teaching on fungal diversity, fungi were collected opportunistically by Summer School trainers and participants in forests, savannah areas, at roadsides, and in cultivated areas, mostly in central Benin (Bellefoungou forest, Kota waterfalls, Okpara forest, Papatia Botanical Garden, Parakou, Taneka, Wari Maro) and in southern Benin (campus of University of Abomey-Calavi, Lokoli forest, Niaouli, Pahou forest, Pobé forest).

Selected fungal specimens collected in 2016 and 2017 were documented by photos and drawings, analysed by light microscopy, dried over 2–3 days using a Dörrex fruit drier at approximately 40 °C during 2–3 days, and stored in plastic bags. They are deposited in the Fungarium of the University of Parakou (UNIPAR). Taxonomic identifications are based on morphological characteristics and data in literature cited in Additional file 17. For some specimens, ITS region molecular sequence data were obtained. For DNA isolation and sequencing methods see Reschke et al. (2018). DNA sequences are deposited in GenBank (for accession numbers see Additional file 17).

## RESULTS

### Species richness of fungi and fungus-like organisms in West Africa

#### Richness of fungal species reported for West Africa

The checklist for fungi and fungus-like organisms in West Africa based on data available in literature contains 16,222 records. These refer to 4843 fungal species and infraspecific taxa (Fig. 1b, Additional files 1, 2). Records were retrieved from 860 publications (Additional file 3), plus 20 records which resulted from recent fieldwork in Benin (see below). If records of species identified only down to genus level are also counted, there are a total of 17,089 records representing 5137 taxa based on 867 references.

The numbers of records, fungal species, and publications analysed for West Africa and each of the West African countries are presented in Fig. 1c-d and Table 1.

**Fig. 2:**
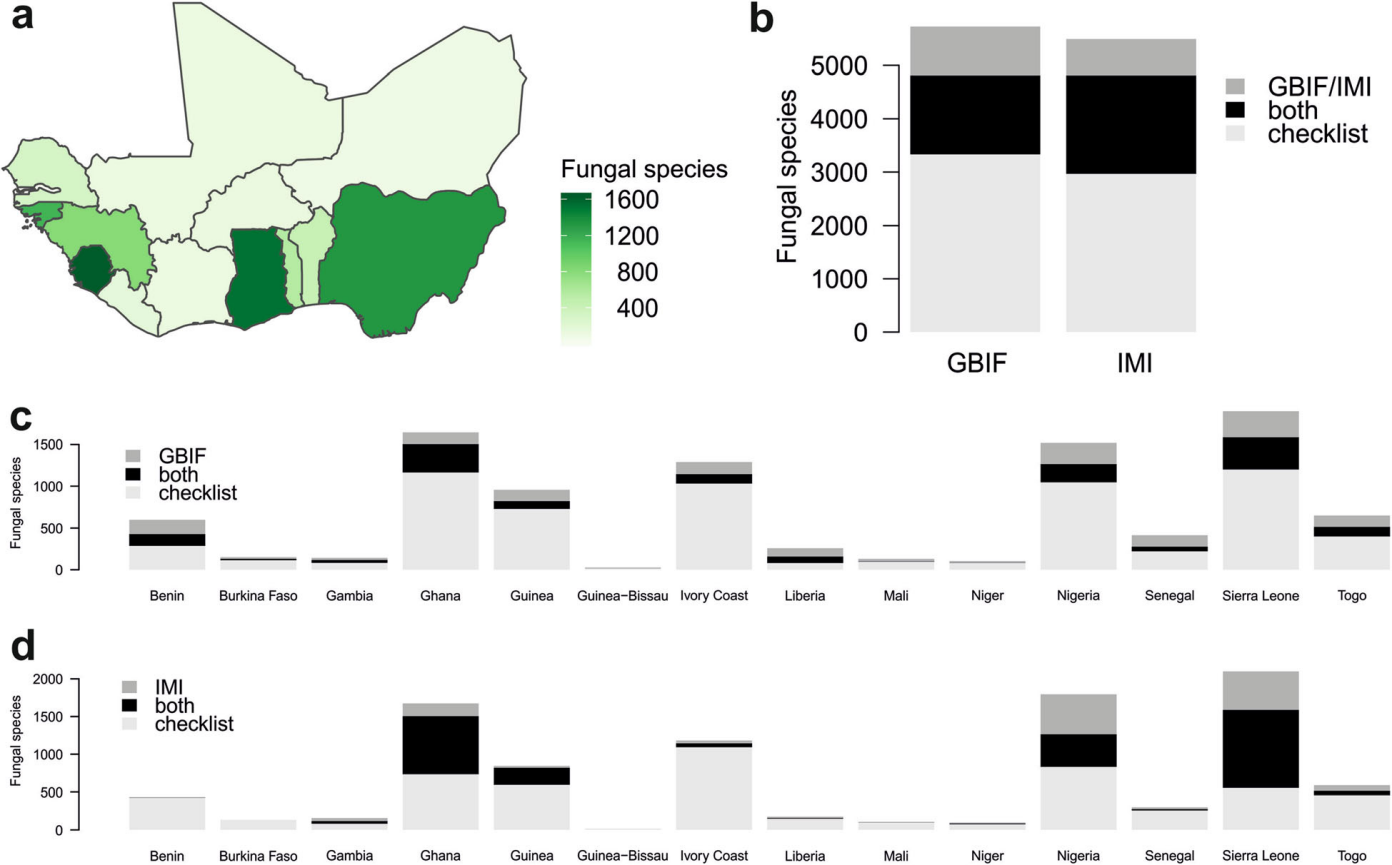
Knowledge of fungal species diversity in West Africa and West African countries. **a** Map of West African countries with colour intensities relative to the number of fungal species known per country according to the checklist based on literature. **b** Number of fungal species known for West Africa by the checklist based on literature only (light grey), by the checklist based on literature and the GBIF database or the IMI fungarium database (black), and only by GBIF/IMI (dark grey). **c** Number of fungal species known for West African countries by the checklist based on literature only (light grey), by this checklist and GBIF (black), and only by GBIF (dark grey). **d** Number of fungal species known for West African countries by the checklist based on literature only (light grey), by this checklist and IMI (black), and only by IMI (dark grey)

The number of publications is maximal for Nigeria (281), with relatively low numbers of species reported in each of the publications (Fig. 1c). Numbers of fungal records vary from a minimum of 20 records referring to 19 fungal species for Guinea Bissau to a maximum of 4121 records referring to 1511 fungal species for Ghana, and 3564 records referring to 1595 species for Sierra Leone (Table 1). For comparison and the analysis of the geographic distribution of data, the knowledge of fungal species known per country is plotted in Fig. 2a with colour intensities relative to the numbers of fungal species known per country.

#### Estimated numbers of fungal species for West Africa

Accumulation curves for fungal species known for West Africa and for West African countries do not reach saturation for any country (Fig. 1b, c). This result shows that the documentation of fungal diversity in these countries is incomplete. Only the curves for Ghana and less obviously for Benin show slight inclinations towards an asymptote (Fig. 1c, d). Numbers of species estimated by the Chao and Bootstrap estimators confirm the incomplete knowledge of fungal diversity for all countries, with values and standard errors well above those reported in other cases (Table 1).

Estimations for the number of fungal species existing in West Africa and West African countries based on the Hawksworth ratio rely on the number of vascular plants known for the respective areas. If we assume a Hawksworth ratio of six fungal species to one plant species, and take the numbers of vascular plant species available in literature, we conclude that 11.4% of the fungal species existing in West Africa are reported (Table 1). Among West African countries, this percentage varies from 0.2% for Guinea Bissau to 14.1% for Sierra Leone (Table 1).

#### Comparison of data found in literature with data in two databases

For the analysis of GBIF data, all records of fungi and fungus-like organisms based on specimens or cultures (not on personal observations) cited for West African countries were retrieved, resulting in a list with 7277 records referring to 2395 species (Additional file 4). Comparison of this list with the checklist based on literature showed that more than half (1481 of 2395) of the fungal species recorded by GBIF are also mentioned by literature analysed for the present checklist (Fig. 2b). Benin, Ghana, Nigeria, and Sierra Leone are represented in GBIF by more than 300 species (Additional file 5). Individual country records in GBIF which are not in the compiled checklist, i.e., that are apparently not yet published, are available in particular for Benin, Guinea Bissau, Liberia, Mali, Senegal, and Togo (Fig. 2c). For these countries, the GBIF records increase the presently known species diversity by at least 25% (Additional file 5).

The IMI data comprise 18,421 records referring to 2526 species (Additional file 6). Almost three quarters of the fungal species mentioned in the IMI database (1847 of 2526) are also mentioned in the checklist based on literature (Fig. 2b). For individual countries, the database of the IMI herbarium provides most records (> 300 fungal species) for Ghana, Nigeria, and Sierra Leone, and new, not yet published data especially (increase of at least 25%) for Gambia, Nigeria, and Sierra Leone (Fig. 2d, Additional file 7).

### History of mycological activities in West Africa

#### Mycological investigation in West Africa in the 18th and nineteenth century

In West Africa, mycological knowledge developed over centuries through indigenous people using fungi for food, medicine, or other purposes, developing complex popular knowledge about the diversity, ecology, and edibility of mushrooms. This knowledge started to be scientifically documented by ethnomycological investigation first in Western West Africa (e.g. Heim 1936, 1940) and later in Nigeria (Ogundana 1979; Zoberi 1973, 1979).

The scientific documentation of fungi in West Africa started with two European naturalists who independently collected fungi in there before the end of the eighteenth century: Ambroise Palisot de Beauvois and Adam Afzelius.

Ambroise Marie François Joseph Palisot, Baron de Beauvois, briefly called **Palisot de Beauvois** (1752/55–1820) was a French naturalist who studied and collaborated with the botanist Antoine Laurent de Jussieu in Paris, France. He became friend of a prince from the Kingdom of Oware (now Nigeria) who visited France towards the end of the eighteenth century and accompanied him to West Africa in 1786 (Palisot de Beauvois 1804; Chase 1925). He became ill soon after his arrival, but he survived and spent 15 months collecting animals (insects), fungi, and plants in the area (Oware; Galbar; Kingdom of Benin, now Nigeria). In 1788, however, his health was so poor that he left for Haiti. After his return to France in 1798, he analysed the specimens that he had sent from Africa to France and published information about the fungi, insects and plants. Information about the fungi was included in “Flore d’Oware et de Benin, en Afrique” (Palisot de Beauvois 1804, 1807). His specimens were cited, e.g. by Fries (1821, 1830, 1836–38) and Santesson (1952; lichens).

**Adam Afzelius** (1750–1837) was a Swedish botanist who was a student of Linnaeus. After moving to England in 1789, he was engaged by the Sierra Leone Company and made two expeditions to the coast of the gulf of Guinea, from March 1792 to August 1793, when he was obliged to return to England for health reasons, and from March 1794 until May 1796 (Kup 1967). At this time, the name “Guinea” referred to the entire coastal area of West Africa. Specimens cited for “Guinea” were most probably collected in what is now Sierra Leone (Acharius 1803). As we do not know where exactly Afzelius worked in the field, fungal specimens may also have been collected in what is now Guinea (Dodge 1953). Data on his fungal specimens were published, e.g. by Acharius (1803, 1806, lichens), Fries (1828, 1830, 1836–38, 1851a, b), Fries and Nyman (1837), and Afzelius and Fries (1860).

**Fig. 3:**
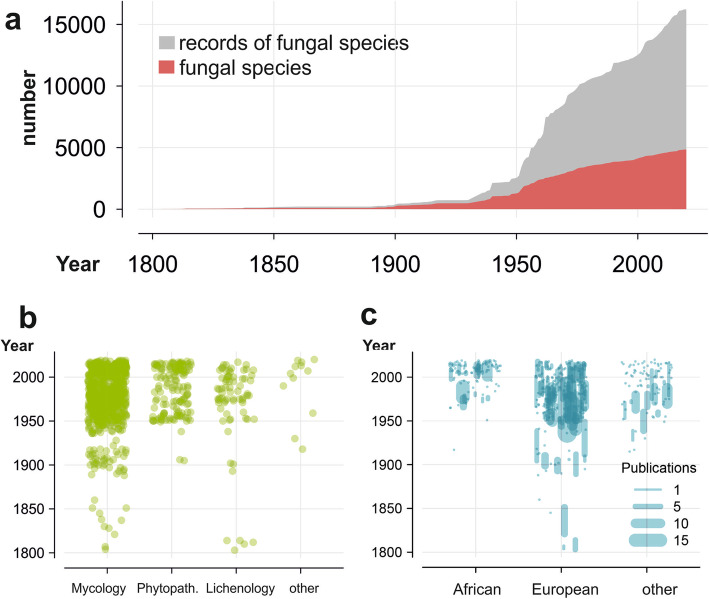
Increase of knowledge on the diversity of fungi and fungus-like organisms in West Africa over time. **a** Knowledge increasing over years. The upper limit of the grey area shows the increasing number of records and the red area the cumulative number of species and infraspecific taxa reported for West Africa. **b** Research fields yielding records of fungal species over time. Publications are attributed to mycological, phytopathological/agricultural, or lichenological research fields. Each green dot corresponds to one publication. **c** Origin of first authors of publications including records of fungal species over time. One dot or line reflects activities of one first author. The length of the lines indicates the range of years in which a given author published. The width of the lines is proportional to the number of publications from that author

Apart from publications in which specimens collected by Afzelius or Palisot de Beauvois are cited, mycological activities were very limited during the nineteenth century (Fig. 3a). At the end of that century, however, the German mycologist and leader of a Research Institute (“Zentralstelle”) for Economic Botany in the German colonies, Paul Hennings, published data on fungal specimens collected by R. Büttner in Togo (Hennings 1893a, 1893b; Timler and Zepernick 1987). The Swiss lichenologist Müller (1893) published data on lichens collected in Sierra Leone by a clergyman called Scott-Elliot.

In the nineteenth century, fungi (lichenized and non-lichenized) and fungus-like organisms were collected mostly by naturalists and published by mycologists or lichenologists. By literature searches for the present work, we found no nineteenth century record of fungi in the context of plant pathology (Fig. 3b).

All these nineteenth century West African records were generated exclusively by European naturalists or mycologists (Fig. 3c, Additional file 8). They received their formation in European colonial powers that were trading with or otherwise active in West African countries.

#### Mycological investigation in West Africa in the twentieth century

During the twentieth century, activities associated with the documentation of fungi in West Africa increased, starting in the 1930s and reaching a peak between 1950 and the early 1960s in numbers of records published (Fig. 3a). Major contributions evident by steps in the accumulation lines in Fig. 3a are mainly due to the publications by Deighton (1936a: 146 species; 1936b: 252 species; and 1956: 324 species mostly reported for Sierra Leone; see also Table 1 footnote), Dade (1940: 444 species mostly reported for Ghana), Hughes (1952: 289; and 1953: 164 species reported mostly for Ghana), and Piening (1962: 934 species mostly reported for Ghana) (Additional file 9). Corresponding steps in the species cumulative curve are not as high as these numbers of records might suggest, because many species were reported more than once. In addition, many published records were cited repeatedly, especially in the context of checklist compilations. As a result, since the 1960s, the total number of records increased much more rapidly than the total number of species known for West Africa.

Since the beginning of the twentieth century and, in particular, since the middle of the twentieth century, publications with fungal records from agricultural and plant pathology sources became increasingly numerous (Fig. 3b). Activities related to the documentation of lichens show a similar pattern except that a few publications including lichens appeared at the beginning of the nineteenth century.

Data plotted in Fig. 3c also show that, until the middle of the twentieth century, only mycologists from Europe and some other countries outside West Africa were first authors of publications on West African fungi. There is, however, one exception: in 1917, Augusta Vera Duthie (born in South Africa, 1881) cited records of slime moulds for West Africa (Duthie 1917). From the 1960s, around the time when the first universities were established in West Africa, African authors started to appear as first authors of publications about West African fungi. Since then, African authors have contributed mycological publications of increasing number and importance.

### Data analysis of systematic groups, species, and diversity patterns

#### Systematic groups of fungal species known for West Africa

After more than two hundred years of mycological investigation, the knowledge of fungi and fungus-like organisms in West Africa remains very patchy across different systematic groups and different countries (Table 2, for complete data see Additional file 10).

**Table 2:**
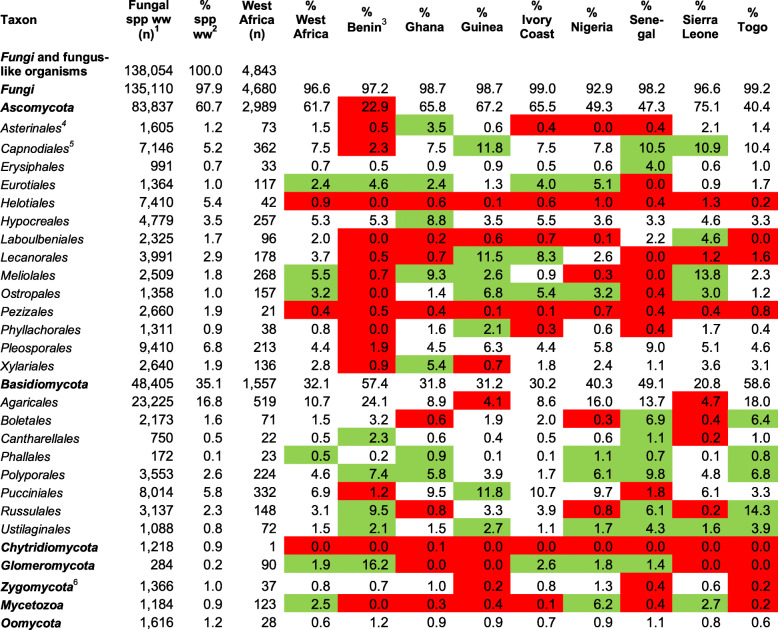
Relatively well known (green) or underexplored (red) systematic groups of fungi in West Africa and West African countries (with at least 200 species known). Numbers highlighted by green indicate groups that present more than double percentage value in comparison to the value worldwide, those highlighted by red indicate understudied groups with less than half of the percentage value in comparison to the value worldwide

^1^ Numbers of fungal species (spp.) known worldwide (ww) for systematic groups of fungi and fungus-like organisms (data from Catalogue of Life, Kirk 2019b)

^2^ Percentage values of systematic groups related to the total number of species known worldwide (138,054)

^3^ Percentage values of systematic groups related to the total number of species known for Benin (432, comp. Table 1). Columns to the right are filled with percentage values resulting from similar calculations

^4^ Orders cited here include more than 100 species known for West Africa or are otherwise relevant for the present study (i.e. *Erysiphales* and *Phallales*)

^5^*Capnodiales* including *Mycosphaerellaceae*

^6^ According to the old systematic concept

As evident by the green coloured numbers in Table 2, groups of relatively conspicuous fungi like *Phallales* (as in Fig. 6f) and *Polyporales* (as in Fig. 6g) are less poorly represented, as are fungi important in applied contexts, such as moulds (*Eurotiales*) and plant pathogens, like cercosporoid fungi (*Capnodiales*), powdery mildews (*Erysiphales* in Senegal; as in Fig. 5b, c), and smut fungi (*Ustilaginales*), that are important in applied research contexts. Relatively high numbers of species in certain groups of fungi for single countries result from the efforts of individual experts; for example for *Laboulbeniales* in Sierra Leone published by Rossi from 1978 to 2013; for *Meliolales* (like Fig. 5d) in Sierra Leone studied mainly by Deighton and Hansford from 1936 to 1963; for *Pucciniales* (like Fig. 6h) in Guinea mainly published by Kranz (1964) and Viennot-Bourgin (1959) and in Nigeria mostly published by Eboh from 1977 to 1989; and for *Mycetozoa* in Nigeria (Ing 1964; Ing and McHugh 1968; Farquharson and Lister 1916).

Red in Table 2 indicates numerous groups of fungal species that are not well represented. These are groups of inconspicuous fungi, like *Chytridiomycota*, *Oomycota*, and *Zygomycota*, and fungi that have not been generally recognized as important in applied contexts, like discomycetes (*Helotiales*, *Pezizales*). For individual countries, the lack of knowledge is particularly striking for *Asterinales* (as in Fig. 5a) in Benin, Ivory Coast, Nigeria, and Senegal, for *Laboulbeniales* in Benin and Togo, for *Xylariales* (as in Fig. 5e, f) in Guinea, for *Agaricales* (as in Figs. 5g-j, 6a-b) in Guinea and Sierra Leone. The lack of knowledge is even more striking for those countries that are not mentioned in Table 2 because fewer than 200 species of fungi are reported for the entire country (comp. Table 1). For Guinea Bissau, for example, according to our data not a single species of *Basidiomycota* has been reported.

The numbers of species known for individual genera are compiled in Additional file 11. Genera with more than 40 species reported for West Africa are mostly plant parasitic microfungi, i.e., black mildews (*Meliola,* as in Fig. 5d), cercosporoid fungi (*Pseudocercospora*, *Cercospora*), rusts (*Aecidium,* as in Fig. 6h, *Puccinia*, *Uredo*), and *Asterinales* (*Asterina*, as in Fig. 5a). *Agaricales* and *Russulales* each include two genera with more than 40 species (*Entoloma* and *Marasmius*, and *Lactifluus* and *Russula*, respectively). Further species-rich genera are moulds (*Eurotiales*, *Aspergillus*) and microfungi associated with arthropods (*Laboulbeniales*, *Laboulbenia*).

#### Analyses of West African fungal diversity at species level

**Fig. 4:**
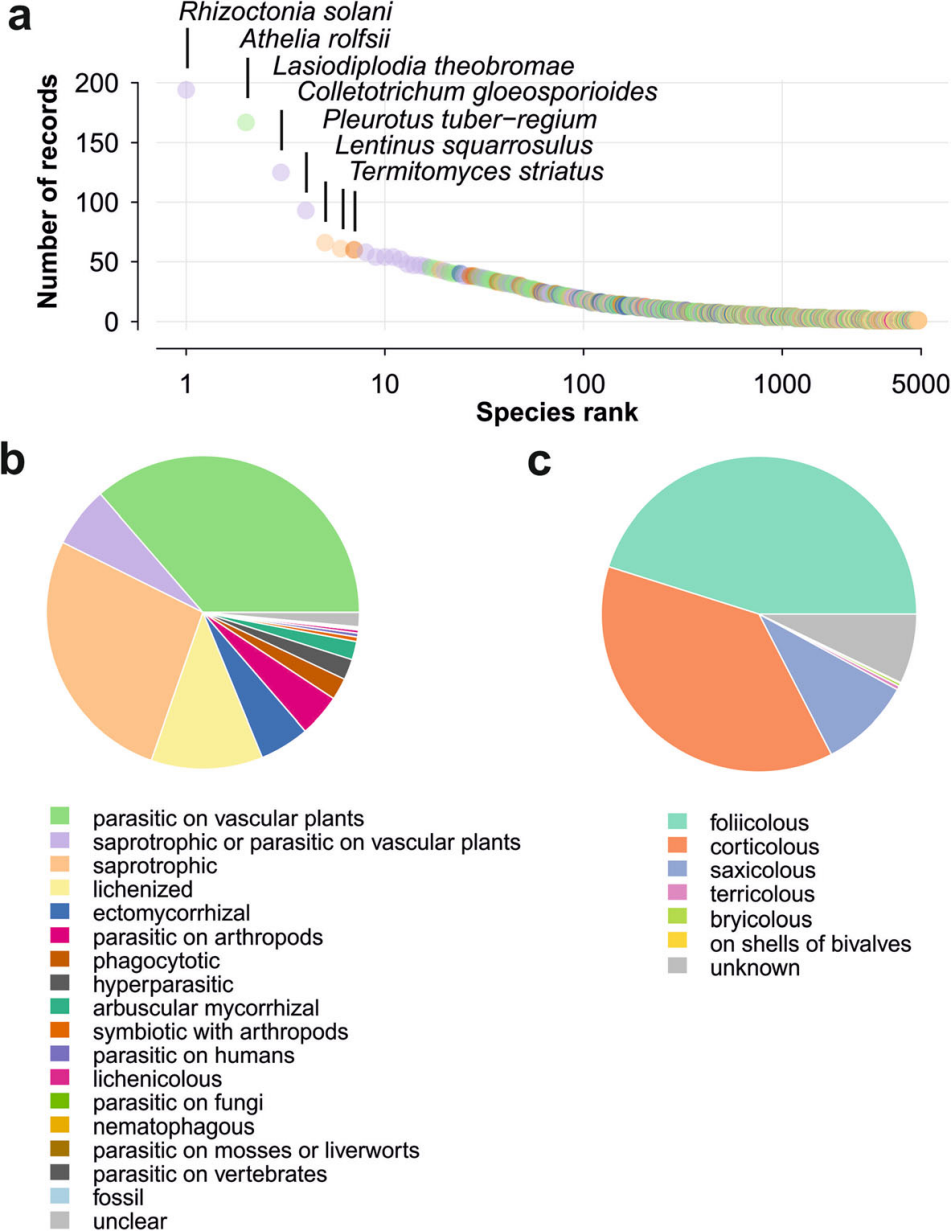
Analyses of records, life forms, and substrata of species of fungi and fungus-like organisms reported for West Africa. **a** Rank abundance curve showing the numbers of records per species. Species ranks are presented in a logarithmic scale. Names of the most frequently reported species are indicated. For the interpretation of colors of dots see Fig. 4b. **b** Relative frequency of life forms of fungal species recorded for West Africa. **c** Relative numbers of species of lichens recorded for different substrata in West Africa. Foliicolous = lichen thallus growing on leaves, corticolous = on bark, saxicolous = on stones or rocks, terricolous = on soil, bryicolous = on bryophytes. If several substrata are cited for the same species of lichen, this species is counted for each of the substrata

Among the 4843 fungal species reported for West Africa, 2195 species (45.3%) have been reported only once, 1026 species (21.2%) have been reported twice, and 461 species (9.5%) have been reported three times (Fig. 4a, Additional file 12). Four species have been recorded more than 80 times, namely *Rhizoctonia solani*, *Athelia rolfsii*, *Lasiodiplodia theobromae*, and *Colletotrichum gloeosporioides s.lat.* These four species are plant pathogens or saprotrophs reported on many different host plant species, mostly in phytopathological publications. The species following in the ranking are two common, conspicuous, and saprotrophic macrofungi, namely *Pleurotus tuber-regium* and *Lentinus squarrosulus*, and *Termitomyces striatus* living in mutualistic symbioses with termites.

As shown in Fig. 4b (for numbers see Additional file 13), most fungal species recorded for West Africa are parasites on vascular plants (36.4%), numerous species are saprotrophic (27.0%) and some species are both, saprotrophs and parasites of plants (6.2%). Other important ecological groups are lichenized fungi (11.5%), ectomycorrhizal fungi (5.1%), and parasites on arthropods (4.4%). Among the lichen fungi (as in Fig. 4c, for numbers see Additional file 14), most species were found on leaves (foliicolous) or on bark (corticolous). Only four species of lichens (0.4%) were documented from soil (terricolous).

Most fungal species (3222 species, 66.5%) have been reported for a single country only, with a further 884 species reported for two countries (18.3%) (Additional file 15). Only 51 species (1.1%) have been reported for most (i.e., more than seven) of the 14 countries that are considered. One fungal species, *Nothopassalora personata*, is reported from 13 countries, seven are known from 12 countries, namely *Cercospora arachidicola*, *Cercospora sorghi*, *Curvularia lunata*, *Epicoccum sorghinum*, *Macrophomina phaseolina*, *Moesziomyces bullatus*, and *Sclerospora graminicola*, all arising from the phytopathological research context. No fungal species has been reported for all 14 countries.

We tested the hypothesis that countries that are geographically closer to each other have more fungal species in common than geographically more distant countries using a Mantel correlogram. The correlations of species similarity across distance classes only yielded low and non-significant correlation values (R = 0.12, *P* = 0.14, Additional file 16), thus indicating no association between distance and shared fungal species.

In total, 1382 names of species and infraspecific taxa (i.e., 28.5% of a total of 4843 species) are based on type specimens collected in West African countries (Table 3). Most of them, i.e., 1262 taxa, are currently accepted, being cited with the original names, or they are combined into other genera or changed from infraspecific to specific level or vice versa (homotypic synonyms). With 547, 266, and 216 types, respectively, Sierra Leone, Ivory Coast, and Ghana are the countries from which the highest numbers of new species have been described.

**Table 3:** The number (n) of types for species or infraspecific taxa originating from West African countries and West Africa. Most of the names are accepted as basionym or as homotypic synonym

	n types	n types names accepted
**Benin**	39	37
**Burkina Faso**	12	12
**Gambia**	2	2
**Ghana**	216	189
**Guinea**	111	106
**Guinea-Bissau**	9	8
**Ivory Coast**	266	247
**Liberia**	32	30
**Mali**	6	6
**Niger**	5	5
**Nigeria**	88	85
**Senegal**	21	19
**Sierra Leone**	547	491
**Togo**	29	26
total	1383	1263
**West Africa**^**a**^	1382	1262

^a^Total minus one because of one type being a syntype originating from two countries

When information for the systematic position of a given species is not available, the term “*incertae sedis*” (“uncertain position”) is used. In the checklist (Additional file 1), there are a total of 1657 records with an *incertae sedis* taxon. For 1051 records, the systematic position at order level is not known and for 1502 records the systematic position at family level is not known. The number of species which are *incertae sedis* at order and/or family level is 583. Most of the taxa *incertae sedis* at order and/or family level are species of *Ascomycota* (448 species), i.e., 15% of the species of *Ascomycota* reported for West Africa.

### Case study: results of fieldwork in Benin

During fieldwork which formed part of Benin Summer School teaching in 2016 and 2017, approximately 200 specimens of fungi from diverse systematic and ecological groups were collected and analysed. Some specimens of macrofungi already well known for Benin through investigations carried out by N.S. Yorou and collaborators were quickly identified, while the identification of specimens representing poorly-studied groups required more detailed analyses and literature research. Some of them probably represent species new to science.

**Fig. 5:**
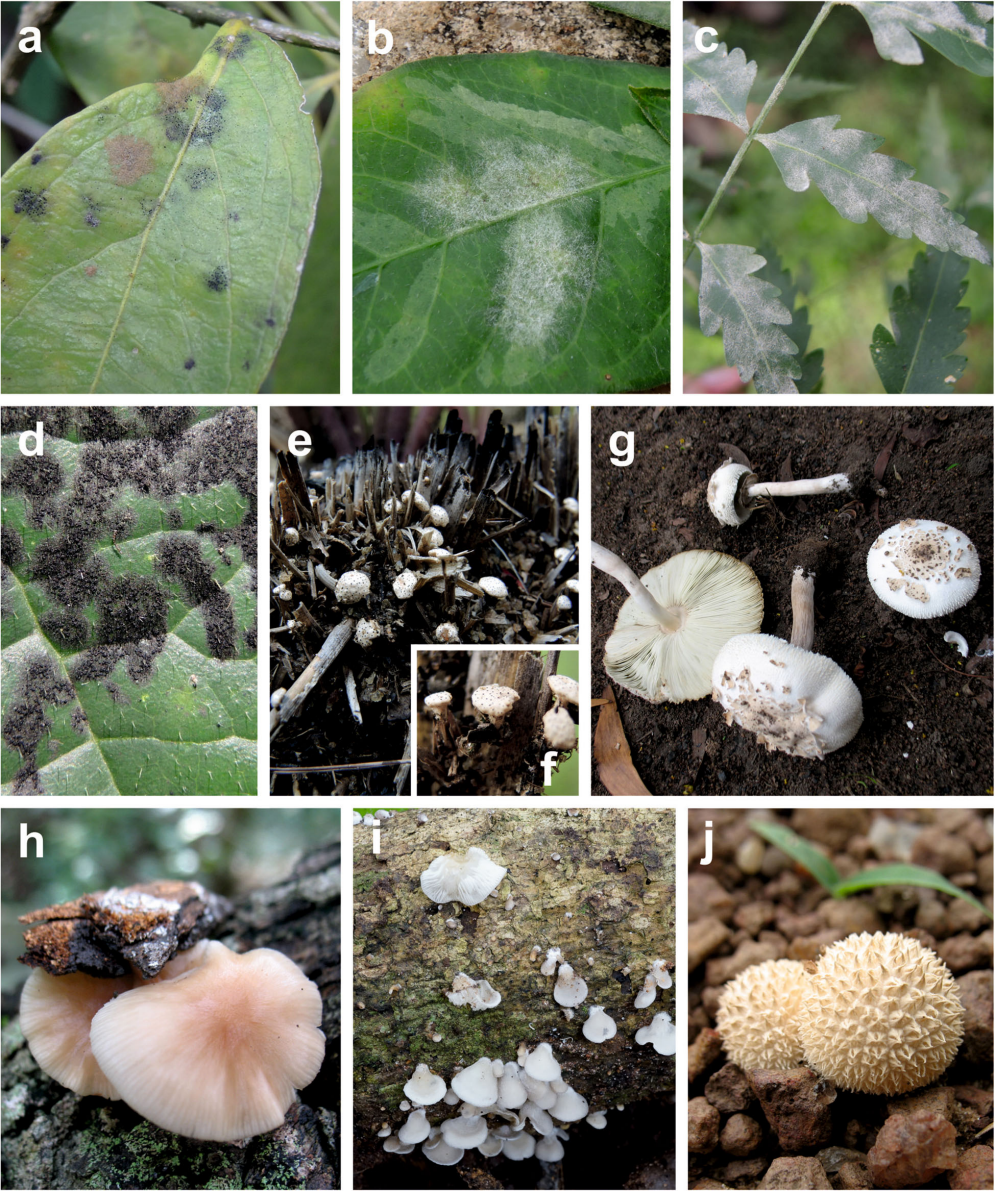
Fungal species reported here for the first time for Benin. **a** Black colonies of *Asterina opiliae* on *Opilia celtidifolia* (MP 5360). **b** Powdery mildew caused by *Leveillula clavata* on *Euphorbia heterophylla* (B 16). **c** Powdery mildew caused by *Pseudoidium azadirachtae* on *Azadirachta indica* (MP 5377). **d** Black mildew caused by *Meliola clerodendricola* on *Clerodendrum capitatum* (MP 5371). **e–f***Podosordaria ustorum* (Taneka 25.7.2017, no specimen). **e** Spore-producing bodies on a burnt tussock of *Andropogon tectorum*. **f** Individual spore-producing bodies with stipes attached to dead grass. **g***Chlorophyllum globosum* (KaiR111). **h.***Gymnopus gibbosus* (KaiR72). **i***Hohenbuehelia* aff. *grisea* (KaiR770). **j***Lycoperdon endotephrum* (KaiR86)

**Fig. 6:**
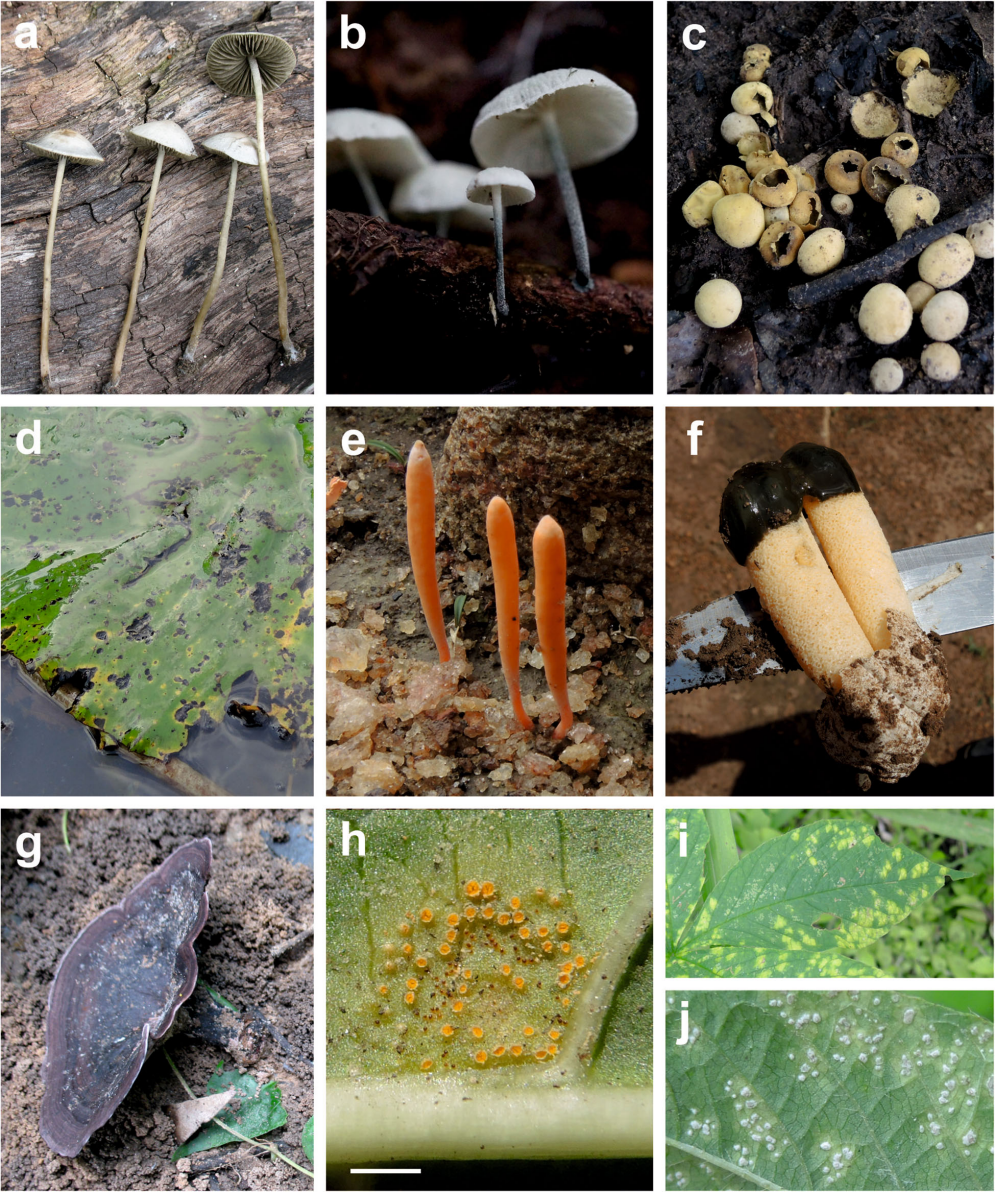
Fungal species reported here for the first time for Benin. **a***Panaeolus bisporus* (KaiR95). **b***Tetrapyrgos atrocyanea* (KaiR783). **c***Scleroderma dictyosporum* (B 06). **d***Rhamphospora nymphaeae* on *Nymphaea* sp. (MP 5382). **e***Sulzbacheromyces miomboensis* (Taneka 26.7.2017, no specimen). Note three clavarioid spore-producing bodies and a thin green lichen thallus covering the soil. **f***Phallus aurantiacus*. **g***Nigroporus stipitatus* (KaiR116). **h** The rust fungus *Aecidium flavidum* on the lower side of a leaf of *Pavetta crassipes* (B11). Note the presence of tiny brown spermatogonia surrounded by aecidia. Scale bar = 2 mm. **i–j** White rust fungus *Albugo ipomoeae-panduratae* on *Merremia aegyptia* (B 22).** i** Lesions evident on the upper side of a leaf. **j** White sori on the lower side of a leaf

Here, we report 20 species of fungi as new for Benin (Figs. 5, 6). They are presented in Additional file 17 by short descriptions, specimen data, references to sequence data, information on host ranges, information on known areas of distribution, discussions of identifications, and references to literature. These records form part of the main checklist file with “present publication” as the literature reference.

Among the 20 species reported as new for Benin, nine are probably also new for West Africa, and among these three have probably never been cited for any African country. *Sulzbacheromyces miomboensis* is the first species of lichenized *Basidiomycota* being reported for West Africa; *Leveillula clavata* and *Pseudoidium azadirachtae* are the first species of *Erysiphales* being reported in internationally available scientific literature for Benin; *Albugo ipomoeae-panduratae* is the first species of *Albuginales* being reported for Benin. *Asterina opiliae* is the second species of *Asterinales*, *Meliola clerodendricola* is the third species of *Meliolales*, and *Podosordaria ustorum* is the fourth species of *Xylariales* known for Benin. As *Asterinales*, *Meliolales*, and *Xylariales* are species rich groups, these results indicate that probably, up to now, no mycologists investigated them for Benin (comp. Table 2).

## DISCUSSION

### Species richness of fungi and fungus-like organisms in West Africa

#### Species richness reported by the checklist

For the first time, information on species of fungi and fungus-like organisms available in literature for West African countries is compiled in a checklist. This checklist contains 4843 species and infraspecific taxa documented by more than 16,000 records in 860 publications. More than 3 years were spent locating literature with records of West African fungi and a team of collaborators helped to retrieve the records from each publication. The checklist is, however, undoubtedly still incomplete, because some relevant publications were not available for analysis. In addition, further publications with records of West African fungi are hidden in old, local, or otherwise inaccessible journals.

In addition to literature records, further information about West African fungi is available as annotated specimens in fungaria (e.g. in Meise Botanic Garden BR, Royal Botanic Gardens Kew K, Muséum National d’Histoire Naturelle Paris PC), culture collections, and databases. The GBIF and IMI databases, for example, contain many unpublished records of fungal species, and they add 914 and 679 species, respectively, taken together 1431 species, to the total knowledge of fungi in West Africa, as well as new records for some of the countries. The IMI database includes detailed additional information on interactions between fungi and the other organisms recorded, and that is crucially important for the understanding of fungal ecology. IMI records generated after May 1989 (not yet available), and other on-line databases, such as Cybertruffle [www.cybertruffle.org.uk/robigalia], MyCoPortal [https://mycoportal.org/portal/index.php], and the USDA Fungal Databases [https://nt.ars-grin.gov/fungaldatabases/] are also likely to have further records. These valuable data were not included in the present checklist because they are beyond the scope of this project.

The records in the checklist were extracted from literature and adjusted to the checklist concept to the best of our knowledge. Nevertheless, there are certainly errors in the checklist data due to:

- Incorrect identifications of species published in the literature.
- Several species are now recognized, from molecular sequence data, as being species complexes including more or less cryptic species. This is the case, e.g. for frequently reported species like *Colletotrichum gloeosporioides* and *Ganoderma lucidum*. With more detailed morphological studies and molecular sequence data, the records would most probably refer to several distinct species.
- Errors in Species Fungorum, such as missing or wrongly cited synonyms. Many have been corrected as a result of this research but undoubtedly other errors remain.
- The checklist includes orphaned species, i.e., species names established in genera now recognized as synonyms, but the particular species have not been re-assigned elsewhere. This happens especially when only few characteristics are provided in the original description of the species, so the concept is difficult or even impossible to apply. Such species names may be scientifically sound but require transfer elsewhere, or be synonyms of other, cited names.
- Information on geographic locations of the records may be erroneous due to country names and borders changing during history.

Despite these limitations, the data on fungi and their substrata provided in this list together with references to original publications are important for fungal conservation, quarantine regulations, and studies of fungal diversity, taxonomy, ecology, and biogeography.

The numbers of fungal species known for West African countries are very heterogeneous, with Sierra Leone, Ghana, and Nigeria presenting the highest numbers (Table 1). These differences are primarily due to strong differences in sampling efforts and number of studies performed in different territories. These differences are also evident in data retrieved from the GBIF and IMI databases. As evident by the map in Fig. 2a, numbers tend to be higher for coastal countries, where a strong influence of European countries led to a faster development of science than in countries distant from the coast. In addition to this geopolitical factor, the climate close to the coast is wetter than inland towards the Sahara. This may have favoured greater development of fungal diversity in the southern part of the investigation area.

#### Known and unknown species diversity

The lists of fungal species for Africa and for West African countries are very far from complete. Only Ghana and Benin show accumulation curves with slight inclinations towards asymptotes. This, however, should not be interpreted as reflecting lists close to completeness, but as a consequence of already known fungal species being cited repeatedly by the same or different authors more frequently than is the case for other countries.

Chao and Bootstrap values are based on the data which were used to calculate the accumulation curves and are therefore strongly influenced by errors arising from incomplete knowledge of fungal diversity. Species diversity is not estimated until reaching saturation because the errors become too large.

For a given country, the maximum of possible species records is the maximum of fungal species names known to mycologists working for this region. This knowledge is limited as many fungal species are not yet described and many described species cannot be identified because of a lack of monographs with keys that would allow all species to be identified. If mycologists working in the field in West Africa could be trained to consider fungi of any group (macro- and microfungi, lichens), and if they would include morphospecies, the numbers would be much higher (cfr. Rudolph et al. 2018). This incomplete knowledge results in saturation curves showing slight and premature tendencies to saturation and in an underestimation of species richness by statistical estimators.

For estimations based on the fungus to plant ratio of 6:1 (Hawksworth 1991), the numbers of vascular plant species are used that are independent from mycological research efforts. They yield hypotheses for the numbers of fungal species existing in the West African countries that are about 7 (Sierra Leone) to 476 (Guinea-Bissau) times as high as the recorded values, and are mostly more than ten times higher than the values yielded by Chao and Bootstrap estimators. For Sierra Leone, the percentage value of fungal species known based on the Hawksworth ratio is higher due to the relatively high number of fungal species and a doubtfully low number of vascular plant species reported for this country (but see Table 1 footnote 5).

We decided to apply the Hawksworth ratio of six fungi to one plant species, although it is a conservative value for this ratio (Hawksworth and Lücking 2017) because it was useful to estimate the diversity that can be directly observed collecting in the field in a given area (Rudolph et al. 2018). Use of environmental sequencing, however, results in many more fungi hidden in diverse substrata being detected. Furthermore the fungus to plant ratio undoubtedly varies at different latitudes (e.g. 17:1 in Alaska: Taylor et al. 2014), tending to increase with higher latitudes (Tedersoo et al. 2014). In addition to this source of error, the estimations for fungi based on known diversity of vascular plants are problematic because that diversity itself is insufficiently known as well (Sosef et al. 2017).

Numbers resulting from these estimations are thus hypothetical and most probably lower than the true fungal species richness that exists in West Africa.

### Systematic groups, history, and diversity patterns of fungi in West Africa

#### Systematic groups of fungal species known for West Africa

The knowledge of species for a given area strongly depends on research efforts of individual specialists, projects, accessibility of areas, and the historical context (Sosef et al. 2017: 10). The fungal specimens forming the basis for the records of West African fungi were at first mostly collected by naturalists (like Afzelius) and identified as well as published by mycologists (including lichenologists) who received collections or found specimens deposited in fungaria. The first fungi collected and repeatedly reported for West African countries were mostly species of *Polyporales* and other macrofungi with persistent spore-producing bodies, as well as lichens. These fungi are easy to preserve in tropical environmental conditions, i.e. with high temperatures and humidity, and are relatively well represented in the checklist. Later, fungi were collected, identified, and published by mycologists or phytopathologists. Other fungi were collected by botanists or entomologists and identified, as well as published, by mycologists. Fungi may be discovered on animal or plant specimens in scientific collections. Spegazzini (1915), for example, discovered species of *Laboulbeniales* on insects collected in West African countries but preserved in Italian museum insect collections.

Most mycologists are specialists working on species diversity of one specific, mostly systematic or ecological, group of fungi. This is mainly because different groups of fungi present specific morphological characteristics and there is a tradition of how the characteristics are used to define species and genera. The uneven and patchy distribution patterns of fungal records shown in Table 2 reflect collection activities, not species diversity patterns in nature. There are strong biases for different systematic groups depending of the speciality of the collectors.

The analysis referring to over- and under-represented systematic groups (Table 2) is based on the assumption that the diversity of systematic groups of fungi is similar all over the world. This may be the case for some orders, but not for all. For example, the relatively high value of species richness of *Russulales* (relative species number in West Africa 3.1% vs. 2.3% worldwide) is not only the consequence of more intensive collection efforts but also the result of species of *Russulales* being important ectomycorrhizal partners of trees in the Soudanian zone (Bâ et al. 2012). Species diversity of *Asterinales*, *Meliolales*, and *Phyllachorales* is higher in the tropics than in extratropical latitudes (e.g. Piepenbring et al. 2011b). For *Meliolales*, this is reflected by present data for West Africa with 5.7% versus 1.8% worldwide. Most species of smut fungi classified in *Ustilaginales* are host specific pathogens on species of *Poaceae*. Their high values probably reflect the high diversity of species of *Poaceae* and high abundance of individual species in the savannahs providing optimal conditions for a high species diversity of these fungi.

#### Ecology of West African fungi

More than one third (43.1%) of fungal species reported for West Africa can live as pathogens of plants. This value is higher than results obtained by previous sampling activities, that showed that about one third of the fungi recorded are parasites of plants (e.g. Piepenbring 2007; Shivas and Hyde 1997). The high percentage is a consequence of numerous phytopathological studies and the focus on cultivated plants. That many plant pathogenic fungi remain to be discovered on wild plants is evident from numbers in Table 2 and from the new records obtained by the fieldwork in Benin.

Approximately 17,500 species of lichenized fungi are known worldwide (cfr. Kirk et al. 2008), representing approximately 12.7% of the total known fungal species diversity known to date. The value of 11.5% for lichens from West Africa is very close to this number. The low number of terricolous lichen species (4 species, 0.4%) is striking (Fig. 4c) and confirmed by field observations in Benin (pers. obs.). In a range of European lichen checklists (Aptroot et al. 1998; Nimis and Tretiach 1995; Nimis et al. 2018; Krause et al. 2017), more than 10% of the lichenized fungal species recorded are terricolous. The very low value for West Africa may be not only because terricolous lichens are reduced there by fire and agricultural activities including trampling by cattle, but also most probably because up to now nobody looked for them.

Although vegetation in tropical lowlands is often dominated by arbuscular mycorrhizal associations (e.g. Piepenbring 2015), ectomycorrhizal fungi are well represented in the checklist with more than 5%. This is largely a consequence of ectomycorrhizal *Isoberlinia* dominated woodlands prevailing in the southern part of the Sudanian Zone.

### Case study: results of fieldwork in Benin

Results of opportunistic sampling of fungi in Benin (Additional file 17) yielded specimens representing new species (e.g. Meswaet et al. 2019, others still being studied), as well as new records of species for Benin, West Africa, and the African continent. These results were obtained as a valuable by-product of mycological teaching activities, and illustrate the incompleteness of knowledge of fungal diversity in Benin.

During the process of identifying specimens from Benin, problems typical for mycological studies in the tropics arose (cfr. Piepenbring et al. 2018):

- Very short (incomplete) species diagnoses do not always allow for the recognition of some species.
- Lack of publications and monographs with detailed descriptions and keys for identification.
- Information spread across a large number of journals, some of them difficult to obtain, even some recent electronic ones because of pay-walls.
- Type specimens are scattered in collections all over the world, some with restrictive policies on loans.
- Lack of molecular sequence data for comparison.

A preliminary version of the checklist provided here was very helpful during the identification process.

In addition to new information concerning the geographical distribution of fungal species, results presented in Additional file 17 include new morphological details (e.g. on spermatogonia in the rust *Aecidium flavidum*), new host species for plant pathogens, and new sequence data.

### Results showing high existing fungal species diversity and incompleteness of knowledge

Diverse observations presented above indicate that the diversity of fungi and fungus-like organisms existing in West Africa is much higher than documented in the present checklist:

- The numbers of records and fungal species known for West African countries are highly variable as a result of more or less effort being directed towards mycological investigation.
- Systematic groups are represented very heterogeneously in the lists of different countries, as a result of scattered efforts of experts.
- All species accumulation curves are far from reaching saturation.
- Estimations are very tentative and yield strongly diverging results.
- More than half of the fungal species (66.5%) known for West Africa have been reported only once (45.3%) or twice (21.2%).
- Most fungal species have been reported only for a single country in West Africa (66.5%). This is most probably the main reason why it was not possible to detect similarities in species composition for geographically close countries (Mantel correlation).
- Numerous fungal species cited for West Africa are incompletely known, as evidenced by many species being only incompletely described and the systematic position of more than 580 species included in the checklist being unknown (*incertae sedis*) at some systematic level.
- It was rather easy to find species new to Benin in particular, and West Africa as a whole by opportunistic sampling during teaching in Benin.
- More than a quarter (28.6%) of the currently accepted fungal species and infraspecific taxa cited in the checklist were described as new based on type specimens from West Africa. This suggests there may be a continuing high potential for the discovery of new species in this area.

### Comparison with knowledge on vascular plants in West Africa

Documentation of plants in West Africa started earlier than the documentation of fungi, because plants are easier to preserve, were perceived by early explorers and naturalists of the colonial administrations to be of high economic interest, and because there were more botanists than mycologists active in West African countries, a situation which continues to this day. However, even for vascular plants, presently available distribution data are incomplete, heterogeneous, and often biased to collection hotspots or specific groups (Sosef et al. 2017; García Márquez et al. 2012; Schmidt et al. 2010).

The sampling of plants in Africa peaked in the 1970s and 1980s (Stropp et al. 2016) slightly later than the peak of mycological publications from 1950 to the early 1960s. Collecting activities in individual countries often peaked with national assessments of plant diversity like flora projects or national checklists. Exploration effort for plants in tropical Africa has diminished since the start of the twenty-first century (Sosef et al. 2017), while mycological activities are steadily increasing. Lack of local mycologists and deficiencies of local infrastructure in West Africa, however, have made progress slow and the documentation of native fungi is still very incomplete (Gryzenhout et al. 2012).

### Conservation

Our knowledge of fungi in West Africa is very incomplete. Why does this matter? Why is it important to know and to conserve fungal diversity? The answer, in the broadest and most simplistic of terms, is that it is not possible to protect producers (plants) and consumers (animals) unless recyclers (fungi) are also conserved.

Even a casual reading of, for example, national reports and biodiversity action plans submitted to the Convention on Biological Diversity (www.cbd.int/reports/search), shows that Governments and their advisors, not just in West Africa, but throughout the world, routinely ignore the fungal species which have been described, and their known ecological roles and properties, and seem unaware of the huge number of other fungi which have not yet been described, and all their still unknown properties (www.fungal-conservation.org/micheli.htm).

The specific roles of fungi in ecosystems and possible uses they may have or problems they may cause to objects and processes of human interest are also ignored. For any fungal strain, even for already known species, exploitable physiological properties may be discovered, like the capacity to produce useful enzymes or compounds with antibiotic or pesticidal properties, or the capacity to promote the growth of cultivated plants. Mycorrhizal fungi are mutualistic symbionts of plants indispensable for the development of forests on poor soils and beneficial for reforestation (e.g. Bâ et al. 2012). On the other hand, and very frequently as a result of human disruption, a fungus which has a natural biocontrol role as one of the checks and balances in an ecosystem may become the agent of new emerging diseases threatening cultivated plants or the health of domesticated animals or human beings (e.g. Burdon 1987; Stukenbrock and McDonald 2008; Kidd et al. 2004). In this case, knowledge about fungi is essential in order to understand what has gone wrong and to develop strategies to rectify it. Similar knowledge, still very incomplete, would enable use of the huge diversity and complex physiological properties of fungi, to mitigate the effects of deforestation, land use changes, or climate change.

Given the enormous importance of fungal diversity for ecosystems and human life, available knowledge about fungi should be applied in order to conserve fungal diversity (May et al. 2018). Africa has lost most of its wild vegetation (Sosef et al. 2017), and, most probably, numerous endemic species of fungi have lost their habitat and became extinct without ever being detected by scientists. For some of the less poorly known groups of fungi, the present checklist can serve as basis for a Red List of fungal species in West Africa evaluated using the criteria of the International Union for the Conservation of Nature (IUCN) as adopted for fungi (Dahlberg and Mueller 2011). Furthermore, fungal species need to be defined in order to evaluate ecosystem health and change.

Based on the very incomplete data made available in the present checklist, it is not possible to detect endemic species. Most species cited can probably be discovered elsewhere by a corresponding collection effort, as long as the required habitat and associated animals, plants, or other hosts are available. In the present study, no analysis has been made of apparently endemic species in order to avoid an overestimation of the area’s endemism richness that can result from incompletely documented ranges of distribution (Küper et al. 2006). Nevertheless, the checklist as presented here is a key step towards recognizing endemic, introduced, or invasive species of fungi.

As the incomplete knowledge of fungal diversity in West Africa will not change soon, it is not appropriate to claim that “we need to know fungal species in order to protect them”. On the contrary: we claim that in the case of fungi “we need to know what we do not know” as this awareness of ignorance should lead us to interact more carefully with our environment.

## CONCLUSIONS

The state of knowledge of fungi and fungus-like organisms in West African countries is typical for tropical countries in that in general less than 10% of the estimated existing diversity is documented. In the tropics, there are numerous and vast areas from which very few or no fungi have been reported, and the knowledge is patchy concerning the coverage of systematic groups due to sporadic activities of specialists (Caribbean area: Minter et al. 2001, Panama: Piepenbring 2007, Venezuela: Iturriaga et al. 2000). To change this situation for West Africa and other parts of the tropics, the following recommendations are made:

- Intensive fieldwork by mycologists from Africa and elsewhere with complementary specialisms is needed in diverse regions in West Africa at different times (seasons) of the year (Hawksworth et al. 1997). This activity will provide fresh specimens which can be analysed by traditional morphological methods, confirming their taxonomy and obtaining barcode sequence data. International experts worldwide should collaborate for diverse systematic and ecological groups, profiting from modern tools for communication. The working conditions in the region for these activities are currently much better than in the past, except where fieldwork is not recommendable at time of political instability.
- We should re-collect and investigate specimens that may correspond to orphaned or little-understood species in order to reject these names or to consolidate the species concepts by providing complete descriptive and molecular sequence data.
- Priority target groups of fungi for sampling activities can be defined by using information on poorly represented systematic groups of fungi in Table 2.
- As shown by the data analysed from the IMI fungarium database, the investigation of preserved specimens can yield interesting additional information on West African fungi.
- We need similar checklists for all countries/continents worldwide to facilitate identification, to increase knowledge of the distribution of species, and for the identification of hotspots of fungal species diversity.
- Culture collections are important for research on applied aspects.
- Citizen science can be integrated, e.g. by portals created by Schmidt & Yorou (https://www.inaturalist.org/projects/fungi-of-tropical-africa). In this context, it is very important to require voucher specimens, as identifications just by observation are not reliable for most species of fungi.
- All of this requires adequate funding.

## Supplementary information

**Additional file 1.** Checklist of fungi known for West Africa based on literature.

**Additional file 2.** Readme, background information for data in Additional file 1.

**Additional file 3.** References to literature containing records of fungi for West African countries and cited in Additional file 1.

**Additional file 4.** Records of fungal species for West Africa retrieved from the GBIF database.

**Additional file 5.** Records of fungal species for West African countries retrieved from the GBIF database in comparison to records in the checklist based on literature.

**Additional file 6.** Records of fungal species for West Africa retrieved from the database of the IMI herbarium.

**Additional file 7.** Records of fungal species for West African countries retrieved from the database of the IMI herbarium in comparison to records in the checklist based on literature.

**Additional file 8.** Information about the origin of first authors of publications containing records of fungi for West Africa.

**Additional file 9.** Numbers of species of fungi reported for West African countries by individual publications.

**Additional file 10.** Numbers of species of fungi known for West Africa per taxon.

**Additional file 11.** Numbers of species of fungi known for West Africa per genus.

**Additional file 12.** Numbers of records per fungal species known for West Africa.

**Additional file 13.** Numbers of West African, fungal species per ecological group.

**Additional file 14.** Numbers of West African lichenized fungal species per substrate.

**Additional file 15.** Numbers of records of fungal species for West African countries.

**Additional file 16.** Mantel correlogram for knowledge of fungal species in West African countries.

**Additional file 17.** Presentation and discussion of fungal species new to Benin.

## Data Availability

Most data generated or analysed during this study are included in this published article and its supplementary information files. Further datasets generated and analysed during the current study are available on internet as indicated in the text.

## ABBREVIATIONS

GBIF: Global Biodiversity Information Facility
IMI: The former International Mycological Institute and its fungal reference collection. The fungarium is now at the Royal Botanic Gardens, Kew (K), while the cultures remained with CAB International at the Institute’s former site in Egham
IUCN: International Union for the Conservation of Nature
