# Supplementary material for: Mapping mycological ignorance – checklists and diversity patterns of fungi known for West Africa
Source: IMA Fungus. 2020 Jul 7;11:13. doi: 10.1186/s43008-020-00034-y (PMC7341642; doi:10.1186/s43008-020-00034-y)
Supplement: Supplementary file 2 — Additional file 2. Readme, background information for data in Additional file 1. [file 43008_2020_34_MOESM2_ESM.docx]

**Readme for Supplement 2**

The Checklist of fungi reported for West Africa (Suppl. 2) contains the following elements in an excel file:

**Status of the scientific name:** correct, synonym, name not found, etc.

**Systematic position:** kingdom or another higher taxon, phylum, order, and family of the species

**Records cited in the context of the description of a new species or infraspecific taxon**:

- “type of a new species” = The name of this species is currently correct as such or a nomenclatural (homotypic) synonym of it. In the case of a newly described variety, this taxon may also be accepted at species level.
- “type of new species (synonym)” = This species is currently considered a taxonomic (heterotypic) synonym.
- “type of a new variety” = This variety is presently accepted.

**Doubtful species identifications:** According to information in literature doubtful species identifications are indicated by the information “cf.” (lat. confer = to compare) or “aff.” (lat. affinis = similar in structure). For doubts resulting from our analyses, see column “comments”.

**Scientific name of the species or infraspecific taxon:** genus, epithet, subspecies or variety, epithet

**Author(s) of names**

**Research context:** The studies including records of fungi in West African countries mostly could be attributed to one of three fields: (i) **mycology**: mycological investigation of fungi (including diversity studies, ecological studies, investigation of ectomycorrhizae, ethnomycology, and cultivation); (ii) **phytopathology** and **agriculture**: investigation of fungi parasitic on cultivated plants by phytopathologists, fungi in agricultural soils, postharvest fungi, fungi on food; (iii) **lichenology**: investigation of lichenized fungi. Until the 1860´s, lichens were not recognized as fungi and treated separately, so there are few studies including lichenized and non-lichenized fungi. Some studies refer to fungi in the medicinal context that were only marginally considered.

**Life forms/ecology:** Fungal species are saprotrophic, mycorrhizal (arbuscular or ectomycorrhizal), symbiotic with termites, parasitic (on fungi, on humans, on arthropods incl. insects, mites, spiders, on mosses or liverworts, on vascular plants, or hyperparasitic, i.e., parasitic on plant parasitic fungi), lichenized, lichenicolous, nematode trapping, or phagocytotic in the case of slime moulds. Numerous plant parasitic fungi can also grow on dead organic material, i.e., saprotrophically. They are annotated as “saprotrophic or parasitic on vascular plants”.

**Substrate/host species (plants, animals, or fungi) and family of the host species.** Names and family positions of host plants are cited according to the International Plant Names Index ([www.ipni.org](http://www.ipni.org)). Names of host organisms were not checked for synonymy.

**Reference to literature:** with page number and eventually the synonymous name under which the record was published

**Country/ies for which the fungal species is reported:** There is one column for each country. A given reference can inform about the existence of a fungal species or infraspecific taxon in one or several countries.

**Comments:** information about problematic identifications, problematic locality interpretation etc.

There is one line for each fungal species reported for one or more West African countries by a given publication and marks (“x”) in the country column(s) for which the species is/are reported. When a given plant pathogenic species was reported from hosts belonging to different families, there is a separate line for each family. In the case of not host specific, plurivorous pathogens the host plants are mentioned as “diverse species of plants” in “diverse families”.

In addition to record lines, there are lines for synonyms that crosslink synonyms with currently used names. Information on synonyms was standardized mostly based on information provided by the Index Fungorum Database (www.indexfungorum.org). During the revision of synonyms and correct names, errors in Index Fungorum / Species Fungorum concerning currently accepted names versus synonyms, lacking species names and combinations were noticed. These data and further pieces of information were incorporated in Index Fungorum / Species Fungorum.
