# Supplementary material for: Mapping mycological ignorance – checklists and diversity patterns of fungi known for West Africa
Source: IMA Fungus. 2020 Jul 7;11:13. doi: 10.1186/s43008-020-00034-y (PMC7341642; doi:10.1186/s43008-020-00034-y)
Supplement: Supplementary file 3 — Additional file 3. References to literature containing records of fungi for West African countries and cited in Additional file 1. [file 43008_2020_34_MOESM3_ESM.docx]

# References to literature cited in Additional file 1

Acharius E (1803) Methodus qua omnes detectos lichenes secundum organa carpomorpha ad genera, species et varietates redigere atque observationibus illustrare tentavit Erich Acharius. F.D.D. Ulrich, typis C.F. Marquard, Stockholm.

Acharius E (1810) Lichenographia universalis. In qua lichenes omnes detectos, adiectis observationibus et figuris horum vegetabilium naturam et organorum carpomorphorum structuram illustrantibus, ad genera, species, varietates differentiis et observationibus sollicite definitas. Apud Iust. Frid. Danckwerts, Gottingae.

Acharius E (1812) Anmarkningar vid Lafslagtet *Thelotrema* med nogare beltammande af dels Arter. Kungl. Svenska Vetenskapasakademiens Handlingar 33:79–95.

Acharius E (1814a) Synopsis methodica lichenum. Litteris et Sumtibus Svanborg et Soc., Lundae.

Acharius E (1814b) Monographie der Lichen-Gattung *Pyrenula*. Magazin Gesellschaft Naturforschender Freunde Berlin 6:3–25.

Acharya K, Paloi S, Dutta AK, Sikder R, Saha T (2017) *Gymnopilus purpureosquamulosus* Høil. (Agaricales, Basidiomycota): A new distributional record from India. Check List 13:1–6. https://doi.org/10.15560/13.2.2064.

Adandonon A, Aveling TAS, Tamo M (2004) Occurrence and distribution of cowpea damping-off and stem rot and associated fungi in Benin. Journal of Agricultural Science 142:561–566. https://doi.org/10.1017/S0021859604004629.

Adandonon A, Datinon B, Baimey H, Toffa J (2014) First report of *Lasiodiplodia theobromae* (Pat.) Griffon & Maubl. causing root rot and collar rot disease of *Jatropha curcas* L. in Benin. Journal of Applied Biosciences 79:6873–6877. https://doi.org/10.4314/jab.v79i1.2.

Adejumo TO, Awosanya, OB (2005) Proximate and mineral composition of four edible mushroom species from South Western Nigeria. African Journal of Biotechnology 4:1084–1088.

Adekoya I, Obadina A, Phoku J, Nwinyi O, Njobeh P (2017) Contamination of fermented foods in Nigeria with fungi. LWT-Food Science and Technology 86:76–84. https://doi.org/10.1016/j.lwt.2017.07.044.

Adekunle VAJ, Ajao K (2005) Contribuitons of edible mushroom (a non-timber forest product of tropical forest ecosystem) to rural livelihood in Oyo state, Nigeria. Pakistan Journal of Biological Science 3:809–812.

Adeniji MO (1970) Fungi associated with storage decay of yam in Nigeria. Phytopathology 60:590–592.

Adewusi SRA, Alofe FV, Odeyemi O, Afolabi A, Oke OL (1993) Studies on some edible wild mushrooms from Nigeria: 1. Nutritional, teratogenic and toxic considerations. Plant Foods for Human Nutrition 43:115–121. https://doi.org/10.1007/BF01087916.

Afiukwa CA, Ugwu Okechukwu PC, Ebenyi LN, Ossai EC, Nwaka AC (2013) Phytochemical analyses of three wild edible mushrooms, coral mushroom, *Agaricus bisporus* and *Lentinus sajor,* common in Ohaukwu Area of Ebonyi State, Nigeria. International Journal of Pharmacy 3:410–414.

Afzelius A, Fries E (1860) Reliquiae Afzelianae, sistensis icones fungorum, quos in Guinea collegit et in aere incisas exudi curavit Adamus Afzelius. Interpretatur E. Fries. Folio (6) pp + 12 engr. aquatint plates. Edquist, Uppsala.

Akpaja EO, Isikhuemhen OS, Okhuoya JA (2003) Ethnomycology and usage of edible and medicinal mushrooms among the Igbo people of Nigeria. International Journal of Medicinal Mushrooms 5:313–319.

Akpaja EO, Okhuoya JA, Ehwerheferere BA (2005) Ethnomycology and indigenous uses of mushrooms among the Bini-speaking people of Nigeria: A case study of Aihuobabekun community near Benin City, Nigeria. International Journal of Medicinal Mushrooms 7:373–374.

Alasoadura SO (1966) Studies in the higher fungi of Nigeria. II. Macrofungi associated with termite nests. Nova Hedwigia 11:387–393.

Alasoadura SO (1967a) Studies in the higher fungi of Nigeria. I. The genus *Termitomyces* Heim. Journal of the West African Science Association 12:139–148.

Alasoadura SO (1967b) Studies in the higher fungi of Nigeria III. Fruiting in *Pleurotus squarrosulus* (Mont.) Sing. Nova Hedwigia 14:327-337, pl. 101-107.

Alasoadura SO (1968a) *Flabellopora crassa* n. gen., n. sp., an aquatic hyphomycete from Nigeria. Nova Hedwigia 15:415–418.

Alasoadura SO (1968b) *Flabellospora verticillata*, a new species of aquatic hyphomycete from Nigeria. Nova Hedwigia 15:419–421.

Alasoadura SO (1968c) Some aquatic hyphomycetes from Nigeria. Transactions of the British Mycological Society 51:535–540. https://doi.org/10.1016/S0007-1536(68)80023-3.

Alasoadura SO (1972) Studies in the higher fungi of Nigeria IV. Some operculate discomycetes. Nova Hedwigia 23:767–780.

Alcorn JL (1990) Additions to *Bipolaris*, *Cochliobolus* and *Curvularia.* Mycotaxon 39:361–392.

Alli OAT, Ogbolu DO, Ademola O, Oyenike MA (2012) Molecular detection of *Pneumocystis jirovecii* in patients with respiratory tract infections. North American Journal of Medical Sciences 4:479–485. https://doi.org/10.4103/1947-2714.101993.

Almborn O (1960) Lichens Africani. Fasc. II (Nos 26-50).

Alofe FV, Odeyemi O, Oke OL (1996) Three edible wild mushrooms from Nigeria: Their proximate and mineral composition. Plant Foods for Human Nutrition 49:63–73.

Amano K (1986) Host range and geographical distribution of the powdery mildew fungi. Japan Scientific Societies Press, Tokyo.

Ames LM (1963 (Reprint 1969)) A monograph of the Chaetomiaceae. Bibliotheca Mycologica 17:1-65, 30 plates.

Antonín V (1998) *Marasmius heinemannianus,* a new edible species from Benin, West Africa. Belgian Journal of Botany 131:127–132.

Antonín V (2003) New species of *Marasmius* (Basidiomycetes, Tricholomataceae) from tropical Africa - I. Sect. *Epiphylli*, *Fusicystides*, *Globulares, Hygrometrici* and *Neosessiles*. Mycotaxon 85:109–130.

Antonín V (2004) New species of marasmioid genera (Basidiomycetes, Tricholomataceae*)* from tropical Africa III. *Marasmius* sect. *Sicci.* Mycotaxon 89:399–422.

Antonín V (2007) Monograph of *Marasmius*, *Gloiocephala, Palaeocephala* and *Setulipes* in tropical Africa. Fungus flora of tropical Africa, Volume 1. National Botanic Garden (Belgium), Meise.

Antonín V (2012) *Chaetocalathus* and *Crinipellis* (Basidiomycota, Marasmiaceae) in tropical Africa: taxonomic and nomenclatural novelties. Cryptogamie Mycologie 33:395–410.

Antonín V (2013a) Monograph of *Crinipellis* and *Chaetocalathus* in tropical Africa. Fungus flora of tropical Africa, vol 3. National Botanic Garden (Belgium), Meise.

Antonín V (2013b) Supplements to the monograph of tropical African species of *Marasmius* (Basidiomycota, Marasmiaceae). Cryptogamie Mycologie 34:113–135.

Antonín V, de Kesel A (2012) *Crinipellis beninensis* (Basidiomycota, Marasmiaceae), a new species from Benin (tropical Africa). Czech Mycology 64:175–180.

Apinis AE, Clark BM (1974) *Neoxenophila foetida* gen. et sp. nov., a new keratinophilic ascomycete. Transactions of the British Mycological Society 63:261–265. https://doi.org/10.1016/S0007-1536(74)80171-3.

Apinis AE, Eggins HOW (1966) *Thermomyces ibadanensis* sp. nov. from oil palm kernel stacks in Nigeria. Transactions of the British Mycological Society 49:629–632. https://doi.org/10.1016/S0007-1536(66)80012-8.

Appiah, AA, Flood J, Bridge PD, Archer, SA (2003) Inter- and intraspecific morphometric variation and characterization of *Phytophthora* isolates from cocoa. Plant Pathology 52:168–180.

Aptroot A (1995) Redispostion of some species excluded from *Didymosphaeria* (Ascomycotina). Nova Hedwigia 60:325–379.

Aptroot A (2001) Lichens from Gambia with a new black-fruiting isidiate *Caloplaca* on savannah trees. Cryptogamie Mycologie 22:265–270.

Arvidsson L (1982) A monograph of the lichen genus *Coccocarpia*. Opera Botanica 67:1–96.

Asaolu V, Odeyinka S, Akinbamijo OO (2012) The effects of four strains of mycorrhizal fungi and goat manure on fodder production by *Moringa oleifera* under rain-fed conditions in the Gambia. Agriculture and Biology Journal of North America 3:365–373. https://doi.org/10.5251/abjna.2012.3.9.365.373.

Aveskamp MM, Verkley GJM, Gruyter J de, Murace MA, Perelló A, Woudenberg JHC, Groenewald JZ, Crous PW (2009) DNA phylogeny reveals polyphyly of *Phoma* section *Peyronellaea* and multiple taxonomic novelties. Mycologia 101:363–382. https://doi.org/10.3852/08-199.

Aveskamp MM, Gruyter J de, Woudenberg JHC, Verkley GJM, Crous PW (2010) Highlights of the Didymellaceae: A polyphasic approach to characterise *Phoma* and related pleosporalean genera. Studies in Mycology 65:1–60. https://doi.org/10.3114/sim.2010.65.01.

Awasthi DD (1975) A monograph of the lichen genus *Dirinaria*. Bibliotheca Lichenologica, vol 2. J. Cramer, Vaduz.

Ayesu-Offei EN (1989) Formae speciales of *Leveillula taurica* (Lév.) Arn. infecting pepper and eggplant in Ghana. Tropical Agriculture 65:355–360.

Ayodele SM, Okhuoya JA (2007) Cultivation studies on *Psathyrella atroumbonata* Pegler. A Nigerian edible mushroom on different agro industrial wastes. International Journal of Botany 3:394–397.

Ayodele SM, Akpaja EO, Adamu Y (2011) Some edible and medicinal mushrooms of Igala Land in Nigeria, their sociocultural and ethnomycological uses. International Journal of Science and Nature 2:473–476.

Bâ AM, Plenchette C, Danthu P, Duponnois R, Guissou T (2000) Functional compatibility of two arbuscular mycorrhizae with thirteen fruit trees in Senegal. Agroforestry Systems 50:95–105. https://doi.org/10.1023/A:1006482904452.

Bâ AM, Diédhiou AG, Prin Y, Galiana A, Duponnois R (2010) Management of ectomycorrhizal symbionts associated to useful exotic tree species to improve reforestation performances in tropical Africa. Annals of Forest Science 67:1–9. https://doi.org/10.1051/forest/2009108.

Bâ AM, Duponnois R, Diabaté M, Dreyfus B (2011) Les champignons ectomycorhiziens des arbres forestiers en Afrique de l'Ouest: Méthodes d'étude, diversité, écologie, utilisation en foresterie et comestibilité. Collection Didactiques. IRD éd, Marseille.

Bâ AM, Duponnois R, Moyersoen B, Diédhiou AG (2012) Ectomycorrhizal symbiosis of tropical African trees. Mycorrhiza 22:1–29. https://doi.org/10.1007/s00572-011-0415-x.

Babacauh KD (1983) Structure des populations de *Phytophthora palmivora* (Butl.) Butl. emend. Bras. et Griff. parasite du Cacaoyer (*Theobroma cacao* L.). Bulletin de la Société Botanique de France 130:15–25.

Babana AH, Antoun H (2006) Effect of Tilemsi phosphate rock-solubilizing microorganisms on phosphorus uptake and yield of field-grown wheat (*Triticum aestivum* L.) in Mali. Plant and Soil 287:51–58. https://doi.org/10.1007/s11104-006-9060-0.

Badou SA, Esel AD, Raspé O, Ryberg MK, Atsu KG, Yorou NS (2018) Two new African siblings of *Pulveroboletus ravenelii* (Boletaceae). MycoKeys 43:115–130. https://doi.org/10.3897/mycokeys.43.30776.

Bako SP, Afolabi S, Funtua II (2008) Spatial distribution and heavy metal content of some bryophytes and lichens in relation to air pollution in Nigeria's Guinea savanna. International Journal of Environment and pollution; Science, Policy, Engineering 33:195–206.

Balazuc J (1980) Laboulbéniales nouvelles (Ascomycètes), parasites de coléoptères et de diptères. Bulletin du Muséum National d'Histoire Naturelle, Ser. 4, Botanique 2:209–219.

Barbosa MAF (1965) A new species of *Neocosmospora* found in stored peanuts. Garcia de Orta Série de Estudos Agrónomicos 13:15–18.

Barreto RW, Evans HC (1998) Fungal pathogens of *Euphorbia heterophylla* and *E. hirta* in Brazil and their potential as weed biocontrol agents. Mycopathologia 141:21–36.

Bartoli A, Maggi O (1978) Four new species of *Aspergillus* from Ivory Coast soil. Transactions of the British Mycological Society 71:383–394. https://doi.org/10.1016/S0007-1536(78)80064-3.

Bartoli A, Maggi O, Persiani AM (1984) *Effetia,* a new genus of Ascomycets from tropical forest soil. Mycotaxon 19:515–522.

Batista AC, Ciferri R (1959) Sistemática dos fungos imperfeitos de picnostromas com himenio invertido (Peltasterales). Mycopathologie et Mycologia Applicata 11:1–102.

Batista AC, da Silva Maia H (1960) *Cirsosia* Arnaud e *Cirsosina* Bat. - Novas espècies. Revista de Biología 2:115–136.

Batista AC, Peres GEP (1960) Um grupo de espécies de Phyllachoraceae da Jamaica 1. Saccardoa 1:48–54.

Batista AC, Vital AF (1957) Contribuição ao estudo dos fungos Sphaeropsidales. Anais da Sociedade de Biologia de Pernambuco 15:413–427.

Batista AC, Vital AF, Maia (1957) Contribuiça o Pernambucana ao estudo dos fungos imperfeitos. Revista de Biologia Lisboa 1:116–124.

Becker U, Lücking R (1995) Foliikole Flechten aus dem Tai-Nationalpark, Elfenbeinküste (Tropisches Afrika): I Neue Arten. In: Daniels FJA (ed) Flechten, Follmann: Contributions to lichenology in honour of Gerhard Follmann. Koeltz Scientific Books, Koenigstein, Germany, pp 161–173.

Beed F, Hotegni Houessou JH, Kelly P, Ezin V (2011) First report of *Aecidium cantense* (potato deforming rust) on *Solanum macrocarpon* (African eggplant) in Bénin. New Disease Reports 23:12. https://doi.org/10.5197/j.2044-0588.2011.023.012.

Beeli M (1938) Étude de la flore mycologique africaine. Note sur des Basidiomycetes recoltés à Sierra Leone par F. C. Deighton. Bulletin du Jardin botanique de l'État à Bruxelles 15:25–53. https://doi.org/10.2307/3666575.

Beenken L, Berndt R (2010) Rust fungi on Annonaceae: the genus *Sphaerophragmium*. Mycologia 102:650–663.

Beenken L, Wood AR (2015) *Puccorchidium* and *Sphenorchidium*, two new genera of Pucciniales on Annonaceae related to *Puccinia psidii* and the genus *Dasyspora*. Mycological Progress 14:1–13. https://doi.org/10.1007/s11557-015-1073-8.

Beenken L, Sainge MN, Kocyan A (2016) *Lactarius megalopterus*, a new angiocarpous species from a tropical rainforest in Central Africa, shows adaptations to endozoochorous spore dispersal. Mycological Progress 15:1–10. https://doi.org/10.1007/s11557-016-1198-4.

Berthet P, Boidin J (1966) Observations sur quelques hymenomycètes récoltés en République Camerounaise. Cahiers la Maboké 4:27–54.

Bi Voko D-RR, Ahonzo-Niamke LL, Zeze A (2013) Impact des propriétés physicochimiques des sols de culture du manioc sur l’abondance et la diversité des communautés de champignons mycorhiziens à arbuscules dans la zone agroécologique d’Azaguie, Sud-Est de la Côte D’Ivoire. Agronomie Africaine 25:251–264.

Bitancourt AA, Jenkins AE (1955) Estudos sobre as Miriangiales V. Elsinoaceas da Costa do Marfim (África Ocidental Francesa). Arquivos do Instituto Biológico de São Paulo 22:69–78.

Blomme G, Ploetz R, Jones D, Langhe E de, Price N, Gold C, Geering A, Viljoen A, Karamura D, Pillay M, Tinzaara W, Teycheney P-Y, Lepoint P, Karamura E, Buddenhagen I (2013) A historical overview of the appearance and spread of *Musa* pests and pathogens on the African continent: highlighting the importance of clean *Musa* planting materials and quarantine measures. Annals of Applied Biology 162:4–26. https://doi.org/10.1111/aab.12002.

Boa E (2004) Wild edible fungi: A global overview of their use and importance to people. Non-wood forest products, vol 17. Food and Agriculture Organization of the United Nations, Rom.

Boamponsem LK, Adam JI, Dampare SB, Nyarko BJB, Essumang DK (2010) Assessment of atmospheric heavy metal deposition in the Tarkwa gold mining area of Ghana using epiphytic lichens. Nuclear Instruments and Methods in Physics Research Section B: Beam Interactions with Materials and Atoms 268:1492–1501. https://doi.org/10.1016/j.nimb.2010.01.007.

Boidin J, Lanquetin P (1975) *Vararia* subgenus *Vararia* (Basidiomycètes Lachnocladiaceae): étude spéciale des espèces. Bulletin de la Société Mycologique de France 91:457–513.

Boidin J, Lanquetin P (1977) *Scytinostroma albo-cinctum* et *S. phaeosarcum* sp. nov. (Basidiomycètes, Lachnocladiaceae). Kew Bulletin 31:621–628.

Boidin J, Lanquetin P (1983) Basidiomycetes Aphyllophorales épitheloïdes étales. Mycotaxon 16:461–499.

Boidin J, Lanquetin P (1987) Le genre *Scytinostroma* Donk. (Basidiomycètes, Lachnocladiaceae). Bibliotheca Mycologica, vol 114. Cramer, Borntraeger, Berlin.

Boidin J, Lanquetin P, Gilles G (1997) Le genre *Gloeocystidiellum* sensu lato (Basidiomycotina). Bulletin de la Société Mycologique de France 113:1–80.

Bongomin F, Gago S, Oladele RO, Denning DW (2017) Global and multi-national prevalence of fungal diseases-estimate precision. Journal of Fungi (Basel, Switzerland) 3:1–29. https://doi.org/10.3390/jof3040057.

Boni S, Yorou NS (2015) Diversité et variabilité inter ethniques dans la consommation de champignons sauvages de la région de N'Dali au Bénin. Tropicultura 33:266–276.

Booth C, Robertson JS (1961) *Leptosphaeria elaeidis* sp. nov. isolated from anthracnosed tissue of oil palm seedlings. Transactions of the British Mycological Society 44:24–26. https://doi.org/10.1016/S0007-1536(61)80002-8.

Boucher H (1918) Les mycoses gommeuses de la Côte d'Ivoire. Bulletin de la Société de Pathologie Exotique 11:306–338.

Bouhot D (1966) Quelques champignons phytopathogènes nouveaux ou peu connus en Sénégal. Bulletin de la Société Mycologique de France 82:274–300.

Brady BL (1980a) *Sarocladium oryzae*, Set 68. CMI 68:No. 673.

Brady BL (1980b) *Sarocladium anntenuatum*, Set 68. CMI 68:No 674.

Brady BL (1986) *Acremonium cajani* sp. nov. (Hyphomycetes) from pigeon pea. Transactions of the British Mycological Society 87:486–487. https://doi.org/10.1016/S0007-1536(86)80232-7.

Braun U, Cook RTA (2012) Taxonomic manual of the Erysiphales (powdery mildews). CBS biodiversity series, vol 11. CBS-KNAW Fungal Biodiversity Centre, Utrecht.

Bresadola J (1890) Fungi Kamerunenses a cl. viro Joanne Braun lecti, additis nonnullis aliis novis, vel criticis ex regio museo bot. berolinensi. Bulletin de la Société Mycologique de France 6:32–49.

Bridge PD, Prior C, Sagbohan J, Lomer CJ, Carey M, Buddie A (1997) Molecular characterization of isolates of *Metarhizium* from locusts and grasshoppers. Biodiversity and Conservation 6:177–189. https://doi.org/10.1023/A:1018387918686.

Buchner P (1912) Studien an intracellularen Symbionten: 1. Die intracellularen Symbionten der Hemipteren. Archiv für Protistenkunde 26:1–116.

Büdel B, Becker U, Porembski S, Barthlott W (1997) Cyanobacteria and cyanobacterial lichens from inselbergs of the Ivory Coast, Africa. Botanica Acta 110:458–465. https://doi.org/10.1111/j.1438-8677.1997.tb00663.x.

Büdel B, Becker U, Follmann G, Sterflinger K (2000) Algae, fungi, and lichens on inselbergs. Ecological Studies 146:69–90.

Busse W (1905) Reisebericht II der pflanzenpathologischen Expedition des kolonialwirtschaftlichen Komitees nach Westafrika. Der Tropenpflanzer 9:169–184.

Busse W (1906) Bericht über die pflanzenpathologische Expedition nach Kamerun und Togo 1904/1905. Beihefte zum Tropenpflanzer 7:163–262.

Buyck B (1993) *Russula* I (Russulaceae). Flore Illustrée des Champignons d'Afrique Centrale 15:335–408, Tf. 55-68.

Buyck B (1994) *Russula* II (Russulaceae). Flore Illustrée des Champignons d'Afrique Centrale 16:411–539, Tf. 69 - 87.

Buyck B (1997) *Russula* III (Russulaceae). Flore Illustrée des Champignons d'Afrique Centrale 17:545-598, Tf. 88-93.

Buyck B, Verbeken A, Eberhardt U (2007) The genus *Lactarius* in Madagascar. Mycological Research 111:787–798. https://doi.org/10.1016/j.mycres.2007.04.006.

Buyck B, Hofstetter V, Eberhardt U, Verbeken A, Kauff F (2008) Walking the thin line between *Russula* and *Lactarius*: the dilemma of *Russula* subsect. *Ochricompactae*. Fungal Diversity 28:15–40.

Calduch M, Gené J, Guarro J, Mercado Sierra A, Castaňeda Ruíz RF (2002) Hyphomycetes from Nigerian rain forests. Mycologia 94:127–135.

Caneva G, Rambelli A (1981) *Danaëa* nuovo genere di ifale demaziaceo. Micologia Italiana 10:47–49.

Cannon PF (1991) A revision of *Phyllachora* and some similar genera on the host family Leguminosae. Mycological Papers, vol 163. CAB International, Wallingford UK.

Carvajal-Campos A, Manizan AL, Tadrist S, Akaki DK, Koffi-Nevry R, Moore GG, Fapohunda SO, Bailly S, Montet D, Oswald IP, Lorber S, Brabet C, Puel O (2017) *Aspergillus korhogoensis*, a novel aflatoxin producing species from the Côte d'Ivoire. Toxins 9:1–22. https://doi.org/10.3390/toxins9110353.

Cassini R, Massenot M (1966) Une méliole nouvelle sur sansevière en Côte-d'Ivoire: *Meliola sansevierae* n. sp. Revue de Mycologie 31:167–170.

Castellano MA, Verbeken A, Walleyn R, Thoen D (2000) Some new or interesting sequestrate Basidiomycota from African woodlands. Karstenia 40:11–21.

Cejp K (1969) Some African species of *Phyllosticta* and *Septoria*. Mycological Papers 117:2–7.

Chadoeuf J, Pierrat JC, Nandris D, Geiger J-P, Nicole M (1993) Modeling rubber tree root disease epidemics with a Markov spatial process. Forest Science 39:41–54.

Chen AJ, Frisvad JC, Sun BD, Varga J, Kocsubé S, Dijksterhuis J, Kim D-H, Hong S-B, Houbraken J, Samson RA (2016) *Aspergillus* section *Nidulantes* (formerly *Emericella*): Polyphasic taxonomy, chemistry and biology. Studies in Mycology 84:1–118. https://doi.org/10.1016/j.simyco.2016.10.001.

Chen J, Xu L-L, Liu B, Liu XZ (2007) Taxonomy of *Dactylella* complex and *Vermispora*. I. Generic concepts based on morphology and ITS sequences data. Fungal Diversity 26:73–83.

Chen SF, Morgan DP, Hasey JK, Anderson K, Michailides TJ (2014) Phylogeny, morphology, distribution, and pathogenicity of Botryosphaeriaceae and Diaporthaceae from English Walnut in California. Plant Disease 98:636–652. https://doi.org/10.1094/PDIS-07-13-0706-RE.

Chevaugeon J (1950) Maladies cryptogamiques du manioc en Côte d'Ivoire 1 - Observations préliminaires sur la nécrose des sommités. Revue de Pathologie Végétale et d'Entomologie Agricole de France 29:3–8.

Chevaugeon J (1951a) Une fusariose du piment en Côte d'Ivoire. Revue de Mycologie, Supplément Colonial 16:81–86.

Chevaugeon J (1951b) *Cercospora personata* (B. et C.) Ellis. Cercosporiose de l'arachide. Revue de Mycologie, Supplément Colonial 16:1–8.

Chevaugeon J (1952) Maladies des plantes cultivées en moyenne-Casamance et dans le delta central nigerien. Revue de Pathologie Végétale et d'Entomologie Agricole de France 31:3–51.

Chevaugeon J (1956a) Les maladies cryptogamiques du manioc en Afrique occidentale. Encyclopédie Mycologique 28:1–205.

Chevaugeon J (1956b) Enquête phytopathologique dans le bassin du Cavally. Revue de Mycologie, Supplément Colonial 21/22:57–86.

Chevaugeon J, Merny G (1956) Maladies des arbres à quinquina en Guinée Française. Journal d'Agriculture Traditionnelle et de Botanique Appliquée 3:605–626. https://doi.org/10.3406/jatba.1956.2340.

Christan J, Yorou NS (2009) *Ramaria sinsinii* sp. nov. und *Ramaria beninensis* sp. nov., zwei neue Ramarien aus Benin (Westafrika). Zeitschrift für Mykologie 75:117–128.

Chupp C (1954) A monograph of the fungus genus *Cercospora*. Publ. by the Author, Ithaca N.Y.

Ciccarone C (1986) *Anthostoma lophirae*, nuova specie di sferiale della foresta di Tai (Costa d’Avorio). Micologia Italiana 15:27–29.

Ciccarone C (1988a) Alcuni saprofiti del genere *Hypoxylon* su legni termitati della foresta di Tai. Micologia Italiana 17:43–47.

Ciccarone C (1988b) *Minimodochium crepuscolare* e *Polynema perlaceum*: due saprofiti foliicoli della foresta equatoriale Africana. Micologia Italiana 17:41–45.

Clay K, Frentz IC (1993) *Balansia pilulaeformis*, an epiphytic species. Mycologia 85:527–534.

Codjia JE, Yorou NS (2014) Ethnicity and gender variability in the diversity, recognition and exploitation of wild useful fungi in Pobè region (Benin, West Africa). Journal of Applied Biosciences 78:6729–6742. https://doi.org/10.4314/jab.v78i1.14.

Cooke MC (1888) Some exotic fungi. Grevillea 16:69-72.

Coppins BJ, Kondratyuk SY (1995) *Stygiomyces* and *Pseudonitschkia:* two new genera of lichenicolous fungi. Edinburgh Journal of Botany 52:229–236.

Corner EJH (1952) Addenda Clavariacea I. Two new pteruloid genera and *Deflexula*. Annals of Botany 16:269–291.

Corner EJH (1966) Clavarioid genera and *Thelephora* from the Congo. Bulletin du Jardin botanique de l'État à Bruxelles 36:257–279.

Costa MEA, Sousa da Camara E (1954) Species aliquae mycologicae Lusitaniae. III. Portugaliae Acta Biologica Sér. B 4:331–345.

Couch JN, Bland CE (eds) (1985) The genus *Coelomomyces*. Acad. Pr, Orlando, Flo.

Coutinho IBL, Freire FCO, Lima CS, Lima JS, Gonçalves FJT, Machado AR, Silva AMS, Cardoso JE (2017) Diversity of genus *Lasiodiplodia* associated with perennial tropical fruit plants in northeastern Brazil. Plant Pathology 66:90–104. https://doi.org/10.1111/ppa.12565.

Crane JL, Schoknecht JD (1981) Revision of *Torula* species. *Pseudoaegerita corticalis*, *Taeniolina deightonii* and *Xylohypha bowdichiae*. Mycologia 73:78–87.

Crouch JA (2014) *Colletotrichum caudatum* s.l. is a species complex. IMA Fungus 5:17–30. https://doi.org/10.5598/imafungus.2014.05.01.03.

Crous PW, Braun U (2003) *Mycosphaerella* and its anamorphs: 1. Names published in *Cercospora* and *Passalora*. CBS biodiversity series, vol 1. Centraalbureau voor Schimmelcultures, Fungal Biodiversity Centre, Institute of the Royal Netherlands Academy of Sciences and Arts, Utrecht, the Netherlands.

Crous PW, Wingfield MJ, Le Roux JJ, Richardson DM, Strasberg D, Shivas RG, Alvarado P, Edwards J, Moreno G, Sharma R, Sonawane MS, Tan YP, Altés A, Barasubiye T, Barnes CW, Blanchette RA, Boertmann D, Bogo A, Carlavilla JR, Cheewangkoon R, Daniel R, de Beer ZW, Jesús Yáñez-Morales M de, Duong TA, Fernández-Vicente J, Geering ADW, Guest D, Held BW, Heykoop M, Hubka V, Ismail AM, Kajale SC, Khemmuk W, Kolařík M, Kurli R, Lebeuf R, Lévesque CA, Lombard L, Magista D, Manjón JL, Marincowitz S, Mohedano JM, Nováková A, Oberlies NH, Otto EC, Paguigan ND, Pascoe IG, Pérez-Butrón JL, Perrone G, Rahi P, Raja HA, Rintoul T, Sanhueza RMV, Scarlett K, Shouche YS, Shuttleworth LA, Taylor PWJ, Thorn RG, Vawdrey LL, Solano-Vidal R, Voitk A, Wong PTW, Wood AR, Zamora JC, Groenewald JZ (2015) Fungal Planet description sheets: 371-399. Persoonia 35:264–327. https://doi.org/10.3767/003158515X690269.

Crous PW, Wingfield MJ, Richardson DM, Le Roux JJ, Strasberg D, Edwards J, Roets F, Hubka V, Taylor PWJ, Heykoop M, Martín MP, Moreno G, Sutton DA, Wiederhold NP, Barnes CW, Carlavilla JR, Gené J, Giraldo A, Guarnaccia V, Guarro J, Hernández-Restrepo M, Kolařík M, Manjón JL, Pascoe IG, Popov ES, Sandoval-Denis M, Woudenberg JHC, Acharya K, Alexandrova AV, Alvarado P, Barbosa RN, Baseia IG, Blanchette RA, Boekhout T, Burgess T, Cano-Lira JF, Čmoková A, Dimitrov RA, Dyakov MY, Dueñas M, Dutta AK, Esteve-Raventós F, Fedosova AG, Fournier J, Gamboa P, Gouliamova DE, Grebenc T, Groenewald M, Hanse B, Hardy GESJ, Held BW, Jurjević Ž, Kaewgrajang T, Latha KPD, Lombard L, Luangsa-ard, JJ, Lysková P, Mallátová N, Manimohan P, Miller AN, Mirabolfathy M, Morozova OV, Obodai M, Oliveira NT, Ordóñez ME, Otto EC, Paloi S, Peterson SW, Phosri C, Roux J, Salazar WA, Sánchez A, Sarria GA, Shin H-D, Silva BDB, Silva GA, Smith MT, Souza-Motta CM, Stchigel AM, Stoilova-Disheva MM, Sulzbacher MA, Telleria MT, Toapanta C, Traba JM, Valenzuela-Lopez N, Watling R, Groenewald JZ (2016) Fungal Planet description sheets: 400-468. Persoonia 36:316. https://doi.org/10.3767/003158516X692185.

Cruz-Laufer AJ, Mardones M, Piepenbring M (2019) Systematics, taxonomy, and distribution of species of *Myriogenospora* G.F. Atk. (Clavicipitaceae, Hypocreales, Ascomycota). Check List 15:735–746. https://doi.org/10.15560/15.5.735.

Cummins GB (1939) New species of Uredinales. Mycologia 31:169–174.

Cummins GB (1941a) New rusts from America and Africa. Bulletin of the Torrey Botanical Club 68:43–48.

Cummins GB (1941b) Descriptions of tropical rusts - IV. Bulletin of the Torrey Botanical Club 68:467–472.

Cummins GB (1945) Descriptions of tropical rusts - VII. Bulletin of the Torrey Botanical Club 72:205–222.

Cummins GB (1952) Uredinales from various regions. Bulletin of the Torrey Botanical Club 79:212–234.

Cummins GB (1956) Descriptions of tropical rusts - VIII. Bulletin of the Torrey Botanical Club 83:221–233.

Cummins GB (1960) Descriptions of tropical rusts - IX. Bulletin of the Torrey Botanical Club 87:31–45.

Dade HA (1940) A revised list of Gold Coast fungi and plant diseases. Bulletin of Miscellaneous Information 1940:205–247.

Dagno K, Lahlali R, Diourté M, Jijakli MH (2012) Fungi occurring on waterhyacinth (*Eichhornia* *crassipes* [Martius] Solms-Laubach) in Niger river in Mali and their evaluation as mycoherbicides. Journal of Aquatic Plant Management 50:25–32.

Damm U, Woudenberg JHC, Cannon PF, Crous PW (2009) *Colletotrichum* species with curved conidia from herbaceous hosts. Fungal Diversity 39:45–87.

Damm U, O'Connell RJ, Groenewald JZ, Crous PW (2014) The *Colletotrichum destructivum* species complex - hemibiotrophic pathogens of forage and field crops. Studies in Mycology 79:49–84. https://doi.org/10.1016/j.simyco.2014.09.003.

Daniëls PP, Ribes Ripoll MA, Christan J (2012) Notas en Gomphales V: Primeros registros de *Ramaria cokeri* R.H. Petersen, para Macaronesia y África. Cryptogamie Mycologie 33:481–488.

Daniëls PP, Hama O, Justo Fernández A, García-Pantaleón FI, Barage M, Ibrahim D, Rosas Alcántara M (2015) First records of some Asian macromycetes in Africa. Mycotaxon 130:337–359. https://doi.org/10.5248/130.337.

Daniëls PP, Baroni T, Hama O, Kluting K, Bergemann S, García-Pantaleón FI, Barage M, Ibrahim D (2017) A new species and a new combination of *Rhodophana* (Entolomataceae, Agaricales) from Africa. Phytotaxa 306:223–233. https://doi.org/10.11646/phytotaxa.306.3.5.

Danquah O-A (1975) Two new species of *Drechslera*. Transactions of the British Mycological Society 64:544–546.

de Clercq D (1983) *Nannizzia cookiella,* a new species of dermatophyte. Mycotaxon 18:23–28.

de Crop E, van de Putte, Wilde S de, Njouonkou AL, de Kesel A, Verbeken A (2016) *Lactifluus foetens* and *Lf*. *albomembranaceus* sp. nov. (Russulaceae): look-alike milkcaps from gallery forests in tropical Africa. Phytotaxa 277:159–170.

de Hoog GS (1974) The genera *Blastobotrys, Sporothrix, Calcarisporium* and *Calcarisporiella* gen. nov. Studies in Mycology 7:1–84.

de Kesel A, Codjia JT, Yorou NS (2002) Guide des champignons comestibles du Bénin. Jardin Botanique National de Belgique; Centre International d'Ecodéveloppement Intégré, Meise, Belgique, Cotonou [Benin].

de Kesel A, Guelly AK, Yorou NS, Codjia J-C (2008) Ethnomycological notes on *Marasmiellus inoderma* from Benin and Togo (West Africa). Cryptogamie Mycologie 29:313–319.

de Kesel A, Yorou NS, Buyck B (2011) *Cantharellus solidus*, a new species from Benin (West-Africa) with a smooth hymenium. Cryptogamie Mycologie 32:277–283. https://doi.org/10.7872/crym.v32.iss3.2011.277.

de Kesel A, Amalfi M, Kasongo Wa Ngoy B, Yorou NS, Raspé O, Degreef J, Buyck B (2016) New and interesting *Cantharellus* from tropical Africa. Cryptogamie Mycologie 37:283–327. https://doi.org/10.7872/crym/v37.iss3.2016.283.

Decock C, Mossebo DC (2002) Studies in *Perenniporia* (Basidiomycetes, Polyporaceae): African taxa III The new species *Perenniporia djaensis* and some records of *Perenniporia* for the Dja Biosphere reserve, Cameroon. Systematics and Geography of Plants 72:55–62.

Decock C, Ryvarden L (2002) Two undescribed *Microporellus* species and notes on *M. clemensiae*, *M. setigerus,* and *M. subincarnatus*. Czech Mycology 54:19–30.

Degelius G (1974) The lichen genus *Collema* with special reference to the extra-European species. Symbolae Botanicae Upsalienses 20:1–215.

Deighton FC (1936a) Preliminary list of fungi and diseases of plants in Sierra Leone. Bulletin of Miscellaneous Information 1936:397–424. https://doi.org/10.2307/4111838.

Deighton FC (1936b) List of fungi collected in Sierra Leone. Bulletin of Miscellaneous Information 7:424–433.

Deighton FC (1944) West African Meliolineae. I. Meliolineae on Malvaceae and Tiliaceae. Mycological Papers 9:1–24.

Deighton FC (1951) New African Meliolaceae. Sydowia, Annales Mycologici Ser. II. 5:1–8.

Deighton FC (1956) Diseases of cultivated and other economic plants in Sierra Leone. Government of Sierra Leone, London.

Deighton FC (1957 (1958)) New African Meliolaceae - II. Sydowia 11:93–115.

Deighton FC (1959) Studies on *Cercospora* and allied genera. I. *Cercospora* species with coloured spores on *Phyllanthus* (Euphorbiaceae). Mycological Papers 71:1–23.

Deighton FC (1960) African fungi. I. Mycological Papers 78:1–43.

Deighton FC (1969a) Microfungi III: New genera and species and redispositions of some hyphomycetes, mainly African. Mycological Papers 117:8–33.

Deighton FC (1969b) Microfungi IV: Some hyperparasitic hyphomycetes and a note on *Cercosporella uredinophila* Sacc. Mycological Papers 118:1-41; 23 figs. 2 plates.

Deighton FC (1972) Four leaf-spotting hyphomycetes from Africa. Transactions of the British Mycological Society 59:419-427. https://doi.org/10.1016/S0007-1536(72)80123-2.

Deighton FC (1973a) *Sclerographiopsis* and *Spinulospora*, two new monotypic hyphomycetous genera from Sierra Leone. Transactions of the British Mycological Society 61:193–196. https://doi.org/10.1016/S0007-1536(73)80104-4.

Deighton FC (1973b) Studies in *Cercospora* and allied genera. IV. *Cercosporella* Sacc*., Pseudocercosporella* gen. nov. and *Pseudocercosporidium* gen. nov. Mycological Papers 133:1–62.

Deighton FC (1974) Studies on *Cercospora* an allied genera. V. *Mycovellosiella* Rangel and a new species of *Ramulariopsis*. Mycological Papers 137:1–75.

Deighton FC (1975) *Sporidesmium brachystegiae* sp. nov. from Sierra Leone. Transactions of the British Mycological Society 64:355–358. https://doi.org/10.1016/S0007-1536(75)80130-6.

Deighton FC (1976) Studies on *Cercospora* and allied genera. VI. *Pseudocercospora* Speg., *Pantospora* Cif. and *Cercoseptoria* Petr. Mycological Papers 140:1–168.

Deighton FC (1979) Studies on *Cercospora* and allied genera. VII. New species and redispositions. Mycological Papers 144:1–56.

Deighton FC (1981) Two species of *Pseudocercospora* from Sierra Leone. Transactions of the British Mycological Society 77:450–453. https://doi.org/10.1016/S0007-1536(81)80058-7.

Deighton FC (1983) Studies on *Cercospora* and allied genera. VIII. Further notes on *Cercoseptoria* and some new species and redispositions. Mycological Papers 151:1–20.

Deighton FC (1985) Some species of *Nodulisporium*. Transactions of the British Mycological Society 85:391–395. https://doi.org/10.1016/S0007-1536(85)80032-2.

Deighton FC (1987) New species of *Pseudocercospora* and *Mycovellosiella*, and new combinations into *Pseudocercospora* and *Phaeoramularia*. Transactions of the British Mycological Society 88:365–391. https://doi.org/10.1016/S0007-1536(87)80011-6.

Deighton FC (1990) A new species of *Cladosporium* causing leaf spots on *Cercestis* in Sierra Leone. Mycological Research 94:570.

Deighton FC, Pirozynski KA (1972) Microfungi. V. More hyperparasitic hyphomycetes. Mycological Papers 128:1–110.

Delassus M, Resplandy R (1955) Observations phytopathologiques sur le maïs en basse Côte d'Ivoire durant la campagne 1953. Riz et Riziculture:69–75.

Denchev TT, Denchev CM (2016a) *Jamesdicksonia anadelphiae-trichaetae* (Georgefischeriales) and *Sporisorium anadelphiae-trichaetae* (Ustilaginales), new species on *Anadelphia trichaeta* (Poaceae) from Guinea. Phytotaxa 252:205–216. https://doi.org/10.11646/phytotaxa.252.3.3.

Denchev TT, Denchev CM (2016b) *Anthracocystis rhytachnes-rottboellioidis* and *A. urelytri,* two new combinations of smut fungi (Ustilaginales) from Africa. Phytotaxa 253:227–231.

Denchev TT, Sipman HJM, Denchev CM (2014) Contribution to the smut fungi (Ustilaginomycetes) of Togo and Benin. Mycobiota 4:25–32. https://doi.org/10.12664/mycobiota.2014.04.02.

Dennis RWG (1950) New fungi. Kew Bulletin 5:170.

Dennis RWG (1953) New species of *Dictyonia* and *Coccomyces*. Kew Bulletin 8:49–50.

Dennis RWG (1955) Fungi from Sierra Leone: Pezizales and Helotiales. Kew Bulletin 10:363–369.

Dennis RWG (1958) Some xylosphaeras of tropical Africa. Revista de Biología 1:175–208.

Dennis RWG (1988) *Melanotaenium majus*, an apparently overlooked African smut. Transactions of the British Mycological Society 90:471–472. https://doi.org/10.1016/S0007-1536(88)80157-8.

Dennis RWG, Reid DA (1957) Some marasmioid fungi allegedly parasitic on leaves and twigs in the tropics. Kew Bulletin 12:287–292.

des Abbayes H (1951) Lichens récoltés en Guinée Française et en Côte d'Ivoire (Mission H. des Abbayes, 1948): III. - Physiacées. Bulletin de l'Institut Français d'Afrique Noire 13:749–761.

des Abbayes H (1958) Lichenes récoltés en Guinée Française et en Côte d'Ivoire: IX. - Supplément aux Parméliacées. Bulletin de l'Institut Français d'Afrique Noire 20:1–27.

des Abbayes H, Motyka J (1950) Lichens récoltés en Guinée française et en Côte d'Ivoire (Mission H. des Abbayes, 1948): I.- Introduction, par H. des Abbayes. Bulletin de l'Institut Français d'Afrique Noire 12:601–610.

Dickinson CH (1968) *Gliomastix* Guéguen. Mycological Papers 115:1–24.

Diedhiou AG, Verpillot F, Gueye O, Dreyfus B, Duponnois R, Bâ AM (2004a) Do concentrations of glucose and fungal inoculum influence the competitiveness of two early-stage ectomycorrhizal fungi in *Afzelia africana* seedlings? Forest Ecology and Management 203:187–194. https://doi.org/10.1016/j.foreco.2004.07.048.

Diédhiou AG, Bâ AM, Sylla SN, Dreyfus B, Neyra M, Ndoye I (2004b) The early-stage ectomycorrhizal thelephoroid fungal sp. is competitive and effective on *Afzelia africana* Sm. in nursery conditions in Sénégal. Mycorrhiza 14:313–322. https://doi.org/10.1007/s00572-003-0276-z.

Diédhiou AG, Gueye O, Diabaté M, Prin Y, Duponnois R, Dreyfus B, Bâ AM (2005) Contrasting responses to ectomycorrhizal inoculation in seedlings of six tropical African tree species. Mycorrhiza 16:11–17. https://doi.org/10.1007/s00572-005-0007-8.

Diédhiou AG, Selosse M-A, Galiana A, Diabaté M, Dreyfus B, Bâ AM, Faria SM de, Béna G (2010) Multi-host ectomycorrhizal fungi are predominant in a Guinean tropical rainforest and shared between canopy trees and seedlings. Environmental Microbiology 12:2219–2232. https://doi.org/10.1111/j.1462-2920.2010.02183.x.

Dietel P (1895) Drei neue Uredineengattungen: *Masseeella, Phakopsora* und *Schizospora*. Berichte der Deutschen Botanischen Gesellschaft 13:332–335.

Dixon PA (1959a) *Sphaerobolus stellatus* Tode in Ghana. Journal of the West African Science Association 5:105–107.

Dixon PA (1959b) Stream spora in Ghana. Transactions of the British Mycological Society 42:174–176. https://doi.org/10.1016/S0007-1536(59)80025-5.

Döbbeler P (1998) Ascomyceten auf der epiphyllen *Radula flaccida* (Hepaticae). Nova Hedwigia 66:325–373.

Döbbeler P (2018) Hypercrealan hyperepiphyllous ascomycetes. In: Blanz P, Poelt J (eds) Biodiversity and ecology of fungi, lichens, and mosses: Kerner von Marilaun Workshop 2015 in memory of Josef Poelt. Verlag der Österreichischen Akademie der Wissenschaften, Budapest, 387-225.

Dodge CW (1953) Some lichenes of tropical Africa. Annals of the Missouri Botanical Garden 40:271–401.

Dodge CW (1971) Some lichens of tropical Africa V: Lecanoraceae to Physciaceae. Beihefte zur Nova Hedwigia 38:1–225.

Doi Y, Abe Y, Sugiyama J (1987) *Trichoderma* Sect. *Saturnisporum*, sect. nov. and *Trichoderma ghanense,* sp. nov. Bulletin of the National Museum of Nature and Science Tokyo, Serie B 13:1–9.

Dominik T, Ihnatowicz A (1975) Soil fungi from Eloka near Abidjan in Equatorial West Africa. Zeszyty Naukowe Akademii Rolniczej W Szczecinie 50:13–27.

Dominik T, Majchrowicz I (1966) Some new species of fungi from the soil of Conakry. Mycopathologia 28:209–219.

Dring DM (1964) Gasteromycetes of West tropical Africa. Mycological Papers 98:1–60.

Dring DM (1980) Contributions towards a rational arrangement of the Clathraceae. Kew Bulletin 35:1–96.

Dring DM, Pegler DN (1978) New and noteworthy gasteroid relatives of the Agaricales from tropical Africa. Kew Bulletin 32:563–569.

Dring DM, Rose AC (1977) Additions to West African phalloid fungi. Kew Bulletin 31:741–751.

Dring VJ (1959) *Phymatotrichum fimicola* sp. nov., a coprophilous hyphomycete. Transactions of the British Mycological Society 42:406–408. https://doi.org/10.1016/S0007-1536(59)80040-1.

Driver F, Milner RJ, Trueman JWH (2000) A taxonomic revision of *Metarhizium* based on a phylogenetic analysis of rDNA sequence data. Mycological Research 104:134–150.

Drouillon R (1951) La maladie des taches brunes de l'arachide. Revue de Mycologie, Supplément Colonial 16:1–11.

Du M, Schardl CL, Nuckles Etta M, Vaillancourt Lisa J (2005) Using mating-type gene sequences for improved phylogenetic resolution of *Collectotrichum* species complexes. Mycologia 97:641–658.

Duan J, Wu W, Liu XZ (2007) *Dinemasporium* (Coelomycetes). Fungal Diversity 26:205–218.

Ducousso M, Bâ AM, Thoen D (2003) Les champignons ectomycorhiziens des forêts naturelles et des plantations d’Afrique de l’Ouest: une source de champignons comestibles. Bois et Forêts des Tropiques 275:51–63.

Duthie AV (1917) African Myxomycetes. Transactions of the Royal Society of South Africa 6:297–310. https://doi.org/10.1080/00359191709520190.

Eboh DO (1978a) A taxonomic survey of Nigerian rust fungi: Uredinales Nigerianensis: I. Mycologia 70:1077–1085. https://doi.org/10.2307/3759139.

Eboh DO (1978b) *Uredo* *andropogonis-gayani* sp. nov. from Nigeria. Transactions of the British Mycological Society 70:150–152. https://doi.org/10.1016/S0007-1536(78)80188-0.

Eboh DO (1981) A taxonomic survey of Nigerian rust fungi. Uredinales Nigerianensis. II. Mycologia 73:445–453. https://doi.org/10.2307/3759598.

Eboh DO (1983a) A new species of *Hapalophragmium* from Nigeria. Mycologia 75:167–169. https://doi.org/10.2307/3792933.

Eboh DO (1983b) A new species of *Newinia* from Nigeria. Mycologia 75:316–318. https://doi.org/10.2307/3792816.

Eboh DO (1983c) A new species of *Sphaerophragmium* on *Dalbergia hostilis* from Nigeria. Transactions of the British Mycological Society 80:160–162.

Eboh DO (1983d) A new species of *Sphaerophragmium* on *Monodora brevipes* from Nigeria. Canadian Journal of Botany 61:2762–2763.

Eboh DO (1984) A taxonomic survey of Nigerian rust fungi: Uredinales Nigerianensis. III. Mycologia 76:179–189. https://doi.org/10.2307/3793093.

Eboh DO (1985a) A re-evaluation of *Ypsilospora*. Transactions of the British Mycological Society 85:39–46.

Eboh DO (1985b) Nigerian graminicolous Uredinales. Grass rusts from Nsukka. II. Mycologia 77:205–211. https://doi.org/10.2307/3793069.

Eboh DO (1986a) A taxonomic survey of Nigerian rust fungi: Uredinales Nigerianensis. IV. Mycologia 78:577–586. https://doi.org/10.2307/3807769.

Eboh DO (1986b) A new variety of *Puccinia substriata* from Nigeria. Transactions of the British Mycological Society 87:476–478.

Eboh DO (1989) A taxonomic study of *Puccinia* species on *Smilax* (Smilacaceae). Sydowia 41:136–143.

Eboh DO, Cummins GB (1980) Species of *Sorataea* (Uredinales). Mycologia 72:203–204. https://doi.org/10.2307/3759434.

Eboh DO, Hennen JF (1987) A re-evaluation of *Aecidium crassocephali*. Transactions of the British Mycological Society 88:134–136. https://doi.org/10.1016/S0007-1536(87)80201-2.

Eboh DO, Obike AO (1977) A preliminary taxonomic study of Nigerian graminicolous Uredinales. Grass rusts from Nsukka. I. Mycologia 69:734–739. https://doi.org/10.2307/3758863.

Ejale AU, Gill LS (1991-1992) Two new species of Myxomycetes from Southern Nigeria. Acta Mycologica 27:267–269.

Ellis MB (1957a) Some species of *Corynespora*. Mycological Papers 65:1–15.

Ellis MB (1957b) Some species of *Deightoniella*. Mycological Papers 66:1–12.

Ellis MB (1957c) *Haplobasidion, Lacellinopsis* and *Lacellina*. Mycological Papers 67:1–27.

Ellis MB (1958) *Clasterosporium* and some allied Dematiaceae - Phragmosporae. I. Mycological Papers 70:1–89.

Ellis MB (1959) *Clasterosporium* and some allied Dematiaceae - Phragmosporae II. Mycological Papers 72:1–75.

Ellis MB (1960) Dematiaceous Hyphomycetes. I. Mycological Papers 76:1–36.

Ellis MB (1961a) Dematiaceous Hyphomycetes. II. Mycological Papers 79:1–22.

Ellis MB (1961b) Dematiaceous Hyphomycetes. III. Mycological Papers 82:1–55.

Ellis MB (1963) Dematiaceous Hyphomycetes. V. Mycological Papers 93:1–32.

Ellis MB (1965) Dematiaceous Hyphomycetes. VI. Mycological Papers 103:1–46.

Ellis MB (1966) Dematiaceous Hyphomycetes. VII. *Curvularia, Brachysporium*, etc. Mycological Papers 106:1–57.

Ellis MB (1967) Dematiaceous Hyphomycetes. VIII. *Periconiella, Trichodochium*, etc. Mycological Papers 111:1–46.

Ellis MB (1968) Dematiaceous Hyphomycetes. IX. *Spiropes* and *Pleurophragmium*. Mycological Papers 114:1–44.

Ellis MB (1971) Dematiaceous Hyphomycetes. Commonwealth Mycological Inst, Kew.

Ellis MB (1972) Dematiaceous Hyphomycetes. XI. Mycological Papers 131:1–25.

Ellis MB (1976 (reprinted1985)) More dematiaceous Hyphomycetes. Commonwealth Mycological Inst, Kew.

Ellison CA, Sawadogo A, Braman S, Nacro S (2015) First report of *Colletotrichum truncatum* causing stem cankers on *Jatropha curcas* in Burkina Faso. Plant Disease 99:14–20. https://doi.org/10.1094/PDIS-02-14-0181-RE.

Elzein A, Brändle F, Cadisch G, Kroschel J, Marley P, Thines M (2008) *Fusarium oxysporum* strains as potential *Striga* mycoherbicides: Molecular characterization and evidence for a new forma specialis. The Open Mycology Journal 2:89–93.

Ertz D (2009) Revision of the corticolous *Opegrapha* species from the paleotropics. Bibliotheca Lichenologica, vol 102. Cramer, Berlin.

Ertz D, Diederich P (2008) Lichens and lichenicolous fungi new for Tenerife (Canary Islands). Cryptogamie Mycologie 29:389–396.

Esimone CO, Ofokansi KC, Adikwu MU, Ibezim EC, Abonyi DO, Odaibo GN, Olaleye DO (2007) In vitro evaluation of the antiviral activity of extracts from the lichen *Parmelia perlata* (L.) Ach. against three RNA viruses. The Journal of Infection in Developing Countries 1:315–320.

Evans HC (1974) Natural control of arthropods, with special reference to ants (Formicidae) by fungi in the tropical high forest of Ghana. Journal of Applied Ecology 11:37–49.

Evans HC, Samson RA (1984) *Cordyceps* species and their anamorphs pathogenic on ants (Formicidae) in tropical forest ecosystems II. The *Camponotus* (Formicinae) complex. Transactions of the British Mycological Society 82:127–150. https://doi.org/10.1016/S0007-1536(84)80219-3.

Evans HC, Shah PA (2002) Taxonomic status of the genera *Sorosporella* and *Syngliocladium* associated with grasshoppers and locusts (Orthoptera, Acridoidea) in Africa. Mycological Research 106:737–744. https://doi.org/10.1017/S0953756202006056.

Evans HC, Johnston PR, Park D, Barreto RW, Soares DJ, Soares DR (2010) *Claviradulomyces*, a new genus of Odontotremataceae from West African rainforest. Fungal Biology 114:41–48. https://doi.org/10.1016/j.mycres.2009.10.003.

Eyi Ndong H, Degreef J, de Kesel A (2011) Champignons comestibles des forêts denses d’Afrique Centrale. Taxonomie et identification. ABC Taxa 10, 253 pp.

Fadeyi OG, Assogba FM, Chabi D, Yorou NS, Gbenou JD (2019) Ethnomycology, myco-chemical analyzes and antioxidant activity of eleven species of the genus *Amanita* (Basidiomycota, fungi) from Benin (West Africa). Journal of Pharmacognosy and Phytochemistry 8:335–341.

Fandohan P, Gnonlonfin BGJ, Hell K, Marasas WFO, Wingfield MJ (2005) Natural occurrence of *Fusarium* and subsequent fumonisin contamination in preharvest and stored maize in Benin, West Africa. International Journal of Food Microbiology 99:173–183. https://doi.org/10.1016/j.ijfoodmicro.2004.08.012.

Farkas E (1987) Foliicolous lichens of the Usambara Montains, Tanzania I. The Lichenologist 19:43–59.

Farquharson CO, Lister G (1916) Notes on South Nigerian Mycetozoa. Journal of Botany 54:121–133.

Farr DF, Elliott M, Rossman AY, Edmonds RL (2005) *Fusicoccum arbuti* sp. nov. causing cankers on pacific madrone in Western North America with notes on *Fusicoccum dimidiatum*, the correct name for *Scytalidium dimidiatum* and *Nattrassia mangiferae*. Mycologia 97:730–741.

Farr ML (1959) O. F. Cook's myxomycete collection from Liberia and the Canary Islands. Lloydia 22:295–301.

Fasidi IO, Kadiri M (1995) Toxicological screening of seven Nigerian mushrooms. Food Chemistry 52:419–422. https://doi.org/10.1016/0308-8146(95)93293-Z.

Fasola TR, Gbolagade JS, Fasidi IO (2007) Nutritional requirements of *Volvariella speciosa* (Fr. ex. Fr.) Singer, a Nigerian edible mushroom. Food Chemistry 100:904–908. https://doi.org/10.1016/j.foodchem.2005.10.061.

Fassatiova O (1967) Notes on the genus *Humicola* Traaen. II. Ceská Mykologie 21:78–89.

Fawole OB, Yahaya JU (2017) Occurrence of fungal strains with herbicidal potentials in agricultural soils of Southern Guinea savanna agro-ecology of Nigeria. Albanian Journal of Agricultural Sciences 16:143–150.

Fennell DI, Raper KB (1955) New species and varieties of *Aspergillus*. Mycologia 47:68–89. https://doi.org/10.2307/3755757.

Fernández-García E, Evans HC, Samson RA (1990) *Hirsutella cryptosclerotium* sp. nov., an entomopathogen of the mealybug pest, *Rastrococcus invadens*, in West Africa. Mycological Research 94:1111–1117. https://doi.org/10.1016/S0953-7562(09)81342-7.

Ferreira BW, Alves JL, Miranda BEC, Barreto RW (2018) Fungi on *Commelina benghalensis* from Brazil, with notes on potential for weed biological control. Tropical Plant Pathology 43:21–35. https://doi.org/10.1007/s40858-017-0189-6.

Feuerer T (2017) Checklists of lichens. https://lichens.hehoe.de/lichens/portalpages/portalpage_checklists_switch.htm. Accessed repeatedly in 2017–2019.

Fisher PJ, Petrini O (1983) Two new pyrenomycetes from submerged wood. Transactions of the British Mycological Society 81:396–398. https://doi.org/10.1016/S0007-1536(83)80093-X.

Fomba SN (1984) Rice disease situation in mangrove and associated swamps in Sierra Leone. Tropical Pest Management 30:73–81.

Friberg S (2001) Distribution and diversity of arbuscular mycorrhizal fungi in traditional agriculture on the Niger inland delta, Mali, West Africa. CBM: s Skriftserie 3:53–80.

Fries E (1821) Systema Mycologicum: Sistens fungorum ordines, genera et species, huc usque cognitas, quas ad normam methodi naturalis determinavit / disposuit atque descripsit Elias Fries, Vol. 1. Ex Officina Berlingiana, Lundae.

Fries E (1828) Commentarius in systematis mycologici. Elenchus Fungorum 1:1–238.

Fries E (1830) Ecologae fungorum, praecique ex herbariis germanorum de scriptum ab Elia Fries. Linnaea 5:497–553.

Fries E (1836-1838) Epicrisis systematis mycologici: seu synopsis Hymenomycetum. sumptibus auctoris, Upsaliae.

Fries E (1851a) Novae symbolae mycologicae, in peregrinis terris a botanicis dancis collectae. Nova acta Regiae Societatis Scientiarum Upsaliensis Serie 3:17–136.

Fries E (1851b) Novarum symbolarum mycologicarum mantissa. Nova acta Regiae Societatis Scientiarum Upsaliensis Serie 3:225–231.

Fries E, Nyman, CM (1837) Adami Afzelii fungi guineenses: quos ad schedulas eet specimina inventoris, descriptos venia ampl. fac. phil. ups. Excudebant Regiae academia typographi, Upsaliae.

Frisch A, Thor G (2010) *Crypthonia*, a new genus of byssoid Arthoniaceae (lichenised Ascomycota). Mycological Progress 9:281–303. https://doi.org/10.1007/s11557-009-0639-8.

Frisvad JC, Skouboe P, Samson RA (2005) Taxonomic comparison of three different groups of aflatoxin producers and a new efficient producer of aflatoxin B1, sterigmatocystin and 3-O-methylsterigmatocystin, *Aspergillus rambellii* sp. nov. Systematic and Applied Microbiology 28:442–453. https://doi.org/10.1016/j.syapm.2005.02.012.

Fulgence KK, Abibatou K, Vincent D, Henriette V, Etienne AK, Kiki-Barro PC, Yavo W, Koné M, Hervé Menan EI (2013) Tinea capitis in schoolchildren in southern Ivory Coast. International Journal of Dermatology 52:456–460. https://doi.org/10.1111/j.1365-4632.2012.05733.x.

Gams W (1971) *Cephalosporium*-artige Schimmelpilze (Hyphomycetes); with an English summary, glossary of morphological terms and keys. Fischer, Stuttgart.

Gams W, de Hoog GS, Samson, RA, Evans HC (1984) The hyphomycete genus *Engyodontium*: a link between *Verticillium* and *Aphanocladium*. Persoonia 12:135–147.

García D, Stchigel AM, Cano J, Guarro J, Hawksworth DL (2004) A synopsis and re-circumscription of *Neurospora* (syn. *Gelasinospora*) based on ultrastructural and 28S rDNA sequence data. Mycological Research 108:1119–1142. https://doi.org/10.1017/S0953756204000218.

Gardt S, Yorou NS, Guissou M-L, Guelly AK, Agerer R (2011) *Amaurodon angulisporus* (Basidiomycota, Fungi), a new species from West Africa identified by molecular and anatomical features. Nova Hedwigia 93:237–247. https://doi.org/10.1127/0029-5035/2011/0093-0237.

Gbolagade JS (2006) Bacteria associated with compost used for cultivation of Nigerian edible mushrooms *Pleurotus tuber-regium* (Fr.) Singer, and *Lentinus squarrosulus* (Berk.). African Journal of Biotechnology 5:338–342.

Gbolagade JS, Fasidi IO (2005) Antimicrobial activities of some selected Nigerian mushrooms. African Journal of Biomedical Research 8:83–87.

Gbolagade JS, Ajayi A, Oku I, Wankasi D (2006) Nutritive value of common wild edible mushrooms from Southern Nigeria. Global Journal of Biotechnology and Biochemistry 1:16–21.

Ge Z-W, Jacobs A, Vellinga EC, Sysouphanthong P, van der Walt R, Lavorato C, An Y-F, Yang ZL (2018) A multi-gene phylogeny of *Chlorophyllum* (Agaricaceae, Basidiomycota): New species, new combination and infrageneric classification. MycoKeys 32:65–90. https://doi.org/10.3897/mycokeys.32.23831.

Gilman GA (1969) An examination of fungi associated with groundnut pods. Tropical Science 11:38–48.

Gjaerum HB (1988) Rust fungi (Uredinales) on Poaceae mainly from Africa. Mycotaxon 31:351–378.

Guissou KML, Sankara P, Guinko S (2005) *Phlebopus sudanicous* ou - la viande des Bobos -, un champignon comestible dans le départment de Satiri au Burkina Faso. Cryptogamie Mycologie 26:195–204.

Guissou KML, Lykke AM, Sankara P, Guinko S (2008) Declining wild mushroom recognition and usage in Burkina Faso. Economic Botany 62:530–539. https://doi.org/10.1007/s12231-008-9028-5.

Guissou KML, Yorou NS, Sankara P, Guinko S (2015) Assessing the toxicity level of some useful mushrooms of Burkina Faso (West Africa). Journal of Applied Biosciences 85:7784–7793. https://doi.org/10.4314/jab.v85i1.5.

Guzmán G (1970) Monografía del género *Scleroderma* Pers. emend. Fr. Darwiniana 16:233–407.

Guzmán-Davalos L, Contu M, Ortega A, Vizzini A, Herrera ML, Ovrebo CL, Rodríguez A, Villalobos, AAR, Palomera V, Vergas G, Senterre A (2008) New morphological molecular data on *Gymnopilus purpureosquamulosus* and its phylogenetic relationships among similar species. Sydowia 60:41–56.

Gwary DM, Ali O, Gwary SD (2007) Management of *Sorghum* smuts and anthracnose using cultivars selection and seed dressing fungicides in Maiduguri, Nigeria. International Journal of Agricultural and Biology 9:324–328.

Hale ME, JR. (1971-1972) New parmeliae (Lichenes) from Africa. Phytologia 22:94–96.

Hale ME, JR. (1973) Studies on the lichen family Thelotremataceae. 1. Phytologia 26:413–420.

Hale ME (1976a) A monograph of the lichen genus *Pseudoparmelia* Lynge (Parmeliaceae). Smithsonian Contributions to Botany, vol 31. Smithsonian Institution Press, Washington.

Hale ME (1976b) A monograph of the lichen genus *Bulbothrix* Hale (Parmeliaceae). Smithsonian Contributions to Botany, vol 32. Smithsonian Institution Press, Washington.

Hale ME (1976c) A monograph of the lichen genus *Parmelina* Hale (Parmeliaceae). Smithsonian Contributions to Botany, vol 33. Smithsonian Institution Press, Washington.

Hale ME, JR. (1990) A synopsis of the lichen genus *Xanthoparmelia* (Vainio) Hale (Ascomycotina, Parmeliaceae). Smithsonian Contributions to Botany, vol 74. Smithsonian Institution Press, Washington.

Hama O, Barage M, Ibrahim D, Adam T, Saadou M (2008) Inventaire et caractérisation des macromycètes des rives du moyen Niger. Annales de l'Université Abdou Moumouni 9-A:1–12.

Hama O, Barage M, Marafa D, Adam T, Saadou M (2009) Détermination de quelques constituants nutritionnels et anti-nutritionnels sur 6 espèces de macromycètes collectées sur les rives du moyen Niger. Annales de l'Université Abdou Moumouni 10-A:45–52.

Hama O, Maes E, Guissou M-L, Ibrahim D, Barage M, Parra Sánchez LA, Raspé O, de Kesel A (2010) *Agaricus subsaharianus,* une nouvelle espèce comestible et consommée au Niger, au Burkina Faso et en Tanzanie. Cryptogamie Mycologie 31:221–234.

Hama O, Ibrahim D, Barage M, Alhou B, Daniëls PP, Infante F (2012) Utilisations de quelques espèces de macromycètes dans la pharmacopée traditionnelle au Niger occidental (Afrique de l’Ouest). Journal of Applied Biosciences 57:4159–4167.

Hambler DJ (1964) The vegetation of granitic outcrops in Western Nigeria. Journal of Ecology 52:573–594.

Hansford CG (1945) Contributions towards the fungus flora of Uganda - VII. New records and revisions (continued). Proceedings of the Linnean Society of London 157:20–41.

Hansford CG (1946a [1944-1945]) Contributions towards the fungus flora of Uganda - VIII New records. Proceedings of the Linnean Society of London 156:138–212.

Hansford CG (1946b) The foliicolous ascomycetes their parasites and associated fungi. Especially as illustrated by Uganda specimens. Mycological Papers 15:1–240.

Hansford CG (1947a) New or interesting tropical fungi - I. Proceedings of the Linnean Society of London 158:28–50.

Hansford CG (1947b) New tropical fungi - II. Proceedings of the Linnean Society of London 159:21–42.

Hansford CG (1949) Tropical fungi - III. New species and revisions. Proceedings of the Linnean Society of London 160:116–153.

Hansford CG (1955) Tropical fungi - V. New species and revisions. Sydowia, Annales Mycologici Ser. II. 9:1–88.

Hansford CG (1957 (1956)) Tropical fungi - VI. Sydowia 10:41–100.

Hansford CG (1958 (1957)) Tropical fungi - VIII. Sydowia 11:44–69.

Hansford CG (1961) The Meliolineae - A monograph. Beihefte zur Sydowia, Annales Mycologici, Ser. II., vol 2. Ferdinand Berger, Horn.

Hansford CG (1963 [1962]) The Meliolineae supplement. Sydowia, Annales Mycologici Ser. II. 16:302–323.

Hansford CG, Deighton FC (1948) West African Meliolineae: II. Meliolineae collected by F.C. Deighton. Mycological Papers 24:1–79.

Hariot P, Patouillard N (1904) Description de champignons nouveaux de l´herbier du Muséum. Bulletin Trimestiel de la Société Mycologique de France 20:61–65.

Hariot P, Patouillard N (1910) Champignons de la région de Tombouctou et de la Mauritanie, recueillis par M. R. Chudeau. Société Mycologique de France 26:205–209.

Härkönen M (1981) Gambian myxomycetes developed in moist chamber cultures. Karstenia 21:21–25. https://doi.org/10.29203/ka.1981.199.

Härkönen M, Buyck B, Saarimäki T, Mwasumbi L (1993) Tanzanian mushrooms and their uses 1. *Russula*. Karstenia 33:11–50. https://doi.org/10.29203/ka.1993.297.

Harris E (1960) *Ramulispora sorghicola* sp. nov. Transactions of the British Mycological Society 43:80–84. https://doi.org/10.1016/S0007-1536(60)80010-1.

Hawksworth DL (1979) The lichenicolous hyphomycetes. Bulletin of the British Museum (National History) 6:184–300.

Hawksworth DL, Santesson R, Tibell L (2011) *Racoleus*, a new genus of sterile filamentous lichen-forming fungi from the tropics, with observations on the nomenclature and typification of *Cystocoleus* and *Racodium*. IMA Fungus 2:71–79. https://doi.org/10.5598/imafungus.2011.02.01.10.

Heim R (1940) Une amanite mortelle de l'Afrique tropicale. Revue de Mycologie 5:22–28.

Heim R (1941) Études descriptives et expérimentales sur les agarics termitophiles d'Afrique tropicale. Mémoires Académie des Sciences de L'Institut de France 64:1–74.

Heim R (1942) Nouvelles études descriptives sur les agarics termitophiles d'Afrique tropicale. Archives du Muséum National d'Histoire Naturelle Serie 6:107–166.

Heim R (1943) Remarques sur les formes primitives ou dégradées de lactario-russulés tropicaux. Boissiera 7:266–280.

Heim R (1945) Les agarics tropicaux à hyménium tubulé. Revue de Mycologie 10:1-64, Plate 1-4.

Heim R (1951) Les *Termitomyces* du Congo belge recueillis par Madame M. Goossens-Fontana. Bulletin du Jardin botanique de l'État à Bruxelles 21:205. https://doi.org/10.2307/3666672.

Heim R (1952) Les *Termitomyces* du Cameroun et du Congo français. Mémoires de la Société Helvétique des Sciences Naturelles 80:1–41.

Heim R (1955) Les lactaires d'Afrique intertropicale (Congo Belge et Afrique Noire Francaise). Bulletin du Jardin botanique de l'État à Bruxelles 25:1–91. https://doi.org/10.2307/3667100.

Heim R (1958) *Termitomyces*. Flore iconographique des champignons du Congo 7:139-151, Plate 23-25.

Heim R (1963) L'armillarielle elegans Heim. Revue de Mycologie 28:89–94.

Heim R (1966) Breves diagnoses latinae novitatum genericarum specificarumque nuper descriptarum. Revue de Mycologie 30:231–241.

Heim R (1970) Breves diagnoses latinae novitatum genericarum specificarumque nuper descriptarum. Revue de Mycologie 34:343–347.

Heim R (1977) Termites et champignons: Les champignons termitophiles d'Afrique Noire et d'Asie meridionale. Faunes et flores actuelles. Soc. Nouvelle des Éds Boubée, Paris.

Heim R, Perreau J (1964) Deux *Boletellus* nouveaux d'Afrique tropicale. Cahiers de la Maboké 2:13–19.

Heinemann P (1954) Notes sur les Boletineae africaines. Bulletin du Jardin botanique de l'État à Bruxelles 24:113–120.

Heinemann P (1988) Novitates generis *Micropsalliotae* (Agaricaceae). Bulletin du Jardin Botanique National de Belgique / Bulletin van de National Plantentuin van België 58:540–543. https://doi.org/10.2307/3668305.

Heinemann P, Rammeloo J (1990) Taxa nova Boletineae. Bulletin du Jardin Botanique National de Belgique / Bulletin van de National Plantentuin van België 60:416–417. https://doi.org/10.2307/3668234.

Hell K, Gnonlonfin BGJ, Kodjogbe G, Lamboni Y, Abdourhamane IK (2009) Mycoflora and occurrence of aflatoxin in dried vegetables in Benin, Mali and Togo, West Africa. International Journal of Food Microbiology 135:99–104. https://doi.org/10.1016/j.ijfoodmicro.2009.07.039.

Hennings P (1893a) Fungi africani. Botanische Jahrbücher für Systematik, Pflanzengeschichte und Pflanzengeographie 14:337–373.

Hennings P (1893b) Fungi africani II. Botanische Jahrbücher für Systematik, Pflanzengeschichte und Pflanzengeographie 17:1–42.

Hennings P (1897a) Fungi Camerunenses I. Botanische Jahrbücher für Systematik, Pflanzengeschichte und Pflanzengeographie 22:72–111.

Hennings P (1897b) Fungi Camerunenses II. Botanische Jahrbücher für Systematik, Pflanzengeschichte und Pflanzengeographie 23:537–558.

Hennings P (1901) Fungi Camerunensis novi III. Botanische Jahrbücher für Systematik, Pflanzengeschichte und Pflanzengeographie 30:39–57.

Hennings P (1905a) Fungi Africae Orientalis IV. Botanische Jahrbücher für Systematik, Pflanzengeschichte und Pflanzengeographie 38:102–118.

Hennings P (1905b) Fungi camerunenses IV. Botanische Jahrbücher für Systematik, Pflanzengeschichte und Pflanzengeographie 38:119–129.

Hernández JR, Eboh DO, Rossman AY (2005) New reports of rust fungi (Uredinales) from Nigeria. Caldasia 27:213–221.

Herrera CS, Rossman AY, Samuels GJ, Lechat C, Chaverri P (2013) Revision of the genus *Corallomycetella* with *Corallonectria* gen. nov. for *Corallomycetella jatrophae* (Nectriaceae, Hypocreales). Mycosystema 32:518–544.

Hjortstam K, Ryvarden L (1981) Studies in tropical Corticiaceae Basidiomycetes III. Two new species of *Laxitextum.* Mycotaxon 13:35–40.

Hjortstam K, Ryvarden L, Watling R (1993) Preliminary checklist of non-agaricoid macromycetes in the Korup National Park, Cameroon and surrounding area. Edinburgh Journal of Botany 50:105–119. https://doi.org/10.1017/S0960428600000743.

Holden M (1970) Notes on the agaric flora of Ghana. Journal of the West African Science Association 15-16:25–34.

Holland JH (1922) The useful plants of Nigeria. Mushrooms. Fungi. Bulletin of Miscellaneous Information 9:881.

Houssou PA, Ahohuendo BC, Fandohan P, Kpodo K, Hounhouigan DJ, Jakobsen M (2009) Natural infection of cowpea (*Vigna unguiculata* (L.) Walp.) by toxigenic fungi and mycotoxin contamination in Benin, West Africa. Journal of Stored Products Research 45:40–44. https://doi.org/10.1016/j.jspr.2008.07.002.

Huang LH (1973) *Zopfiella flammifera*, a new species from Nigerian soil. Mycologia 65:690–694.

Huang LH, Raper KB (1971) *Aspergillus longivesica*, a new species from Nigerian soil. Mycologia 63:50–57. https://doi.org/10.2307/3757684.

Hughes SJ (1951a) Studies on micro-fungi. VII. *Allescheriella crocea, Oidium simile,* and *Pellicularia pruinata*. Mycological Papers 41:1–17.

Hughes SJ (1951b) Studies on micro-fungi. IX. *Calcarisporium, Verticladium* and *Hansfordia* (gen. nov.). Mycological Papers 43:1–25.

Hughes SJ (1951c) Studies on micro-fungi. X. *Zygosporium.* Mycological Papers 44:1–18.

Hughes SJ (1951d) Studies on micro-fungi. XI. Some hyphomycetes which produce phialides. Mycological Papers 45:1–36.

Hughes SJ (1951e) Studies on micro-fungi. XII. *Triposporium, Tripospermum, Ceratosporella,* and *Tetraposporium* (gen.nov.). Mycological Papers 46:1–35.

Hughes SJ (1951f) Studies on micro-fungi. XIII. *Beltrania, Ceratocladium, Diplorhinotrichum* and *Hansfordiella* (gen. nov.). Mycological Papers 47:1–15.

Hughes SJ (1952a) Fungi from the Gold Coast. I. Mycological Papers 48:1–91.

Hughes SJ (1952b) Studies on micro-fungi. XIV. *Stigmella, Stigmina, Camptomeris, Polythrincium* and *Fusicladiella*. Mycological Papers 49:1–25.

Hughes SJ (1953) Fungi from the Gold Coast. II. Mycological Papers 50:1–104.

Huneck S, Follmann G (1975) Mitteilungen über Flechteninhaltsstoffe CX: Zur Phytochemie und Chemotaxonomie einiger *Usnea*-Arten. 2. Philippia 2:276–282.

Hyde KD, Cannon PF (1999) Fungi causing tar spots on palms. Mycological Papers 175:1–111.

Hywel-Jones NL, Samuels GJ (1998) Three species of *Hypocrella* with large stromata pathogenic on scale insects. Mycologia 90:36–46. https://doi.org/10.2307/3761009.

Ihayere CA, Oghenekaro AO, Osemwegie OO, Okhuoya JA (2010) Chemical nature of *Ganoderma lucidum* (Curtis) Karsten from woodlands of Edo State, Nigeria. Continental Journal of Biological Sciences 3:8–15.

Ing B (1964) Myxomycetes from Nigeria. Transactions of the British Mycological Society 47:49–55. https://doi.org/10.1016/S0007-1536(64)80079-6.

Ing B (1967) Myxomycetes from Sierra Leone. Transactions of the British Mycological Society 50:549-533.

Ing B, McHugh R (1968) Myxomycetes from Nigeria. II. Transactions of the British Mycological Society 51:215–220.

Ingleby K, Walker C, Mason, PA (1994) *Acaulospora excavata* sp. nov. - an endomycorrhizal fungus from Côte d'Ivoire. Mycotaxon 50:99–105.

Ingold CT (1956) Stream spora in Nigeria. Transactions of the British Mycological Society 39:108–110. https://doi.org/10.1016/S0007-1536(56)80058-2.

Ingold CT (1959) Aquatic spora of Omo Forest, Nigeria. Transactions of the British Mycological Society 42:479–485. https://doi.org/10.1016/S0007-1536(59)80049-8.

Ite AE, Udousoro, II, Ibok, UJ (2014) Distribution of some atmospheric heavy metals in lichen and moss samples collected from Eket and Ibeno local government areas of Akwa Ibom State, Nigeria. American Journal of Environmental Protection 2:22–31. https://doi.org/10.12691/env-2-1-5.

Jardiné S, Magloire L (1965) Sénégal - Côte d'Ivoire. Palynologie et stratigraphie du crétacé des bassins du Sénégal et de Côte d'Ivoire. Mémoires du Bureau de Recherches Géologiques et Minières 32:187–222.

Jeger MJ, Gilijamse E, Bock CH, Frinking HD (1998) The epidemiology, variability and control of the downy mildews of pearl millet and sorghum, with particular reference to Africa. Plant Pathology 47:544–569. https://doi.org/10.1046/j.1365-3059.1998.00285.x.

Johansen I, Ryvarden L (1979) Studies in the Aphyllophorales of Africa. VII. Some new genera and species in the Polyporaceae. Transactions of the British Mycological Society 72:189–199.

Johnson J-M, Houngnandan P, Kane A, Sanon KB, Neyra M (2013) Diversity patterns of indigenous arbuscular mycorrhizal fungi associated with rhizosphere of cowpea (*Vigna unguiculata* (L.) Walp.) in Benin, West Africa. Pedobiologia 56:121–128. https://doi.org/10.1016/j.pedobi.2013.03.003.

Johnson J-M, Houngnandan P, Kane A, Chatagnier O, Sanon KB, Neyra M, van Tuinen D (2015) Colonization and molecular diversity of arbuscular mycorrhizal fungi associated with the rhizosphere of cowpea (*Vigna unguiculata* (L.) Walp.) in Benin (West Africa): An exploratory study. Annals of Microbiology 66:207–221. https://doi.org/10.1007/s13213-015-1097-y.

Johnson TW Jr., Seymour RL, Padgett DE (2005) Systematics of the Saprolegniaceae: New taxa. Mycotaxon 92:1–10.

Jones FR (1964) *Nematoctonus robustus* sp. nov. Transactions of the British Mycological Society 47:57–60. https://doi.org/10.1016/S0007-1536(64)80080-2.

Jonsyn FE (1988) Seedborne fungi of sesame (*Sesamum indicum* L.) in Sierra Leone and their potential aflatoxin/mycotoxin production. Mycopathologia 104:123–127. https://doi.org/10.1007/BF00436937.

Kamou H, Nadjombe P, Guelly AK, Yorou NS, Maba DL, Akpagana K (2015) Les champignons sauvages comestibles du Parc National Fazao-Malfakassa (PNFM) au Togo (Afrique de l'Ouest): Diversité et connaissances ethnomycologiques. Agronomie Africaine 27:37–46.

Kamou H, Gbogbo KA, Yorou NS, Nadjombe P, Abalo-loko AG, Verbeken A, de Kesel A, Batawila K, Akpagana K, Guelly AK (2017) Inventaire préliminaire des macromycètes du Parc National Fazao-Malfakassa du Togo, Afrique de l´Ouest. Tropicultura 35:275–287.

Kane M, Courtecuisse R (2013) Liste préliminaire des Agaricomycotina (Basidiomycota, Fungi) du Sénégal. Documents Mycologiques 35:29–45.

Kane M, Noba K, Moreau P-A, Courtecuisse R (2013) Note sur quelques espèces de macromycètes (Basidiomycota, Fungi) nouvelles pour la gonge du Sénégal. Documents Mycologiques 35:3–28.

Kawakami S-I, Hagiwara H (2008) *Polysphondylium multicystogenum* sp. nov., a new dictyostelid species from Sierra Leone, West Africa. Mycologia 100:347–351.

Kee YJ, Suhaimi NN, Zakaria L, Mohd MH (2017) Characterisation of *Neoscytalidium dimidiatum* causing leaf blight on *Sansevieria trifasciata* in Malaysia. Australasian Plant Disease Notes 12:60 (pages 1–4). https://doi.org/10.1007/s13314-017-0284-z.

Kehinde IA (2013) Characteristic symptoms of melon diseaes caused by fungi in south western Nigeria. African Journal of Agricultural Research 8:5791–5801.

Kellog CA, Griffin DW, Garrison VH, Peak KK, Royall N, Smith RR, Shinn EA (2004) Characterization of aerosolized bacteria and fungi from desert dust events in Mali, West Africa. Aerobiologia 20:99–110. https://doi.org/10.1023/B:AERO.0000032947.88335.bb.

Kern H (1959) Über einige Ascomyceten von der Elfenbeinküste. Berichte der Schweizerischen Botanischen Gesellschaft = Bulletin de la Société Botanique Suisse 69:277–285. https://doi.org/10.5169/seals-48677.

Khemmuk W, Shivas RG, Henry RJ, Geering ADW (2016) Fungi associated with foliar diseases of wild and cultivated rice (*Oryza* spp.) in northern Queensland. Australasian Plant Pathology 45:297–308. https://doi.org/10.1007/s13313-016-0418-3.

Kini KR, Leth V, Mathur SB (2002) Genetic variation in *Fusarium moniliforme* isolated from seeds of different host species from Burkina Faso based on random amplified polymorphic DNA analysis. Journal of Phytopathology 150:209–212. https://doi.org/10.1046/j.1439-0434.2002.00739.x.

Kirk PM (1980) *Pseudocercospora abelmoschi*, Set 68. CMI 68:No. 678.

Klaubauf S, Tharreau D, Fournier E, Groenewald JZ, Crous PW, Vries RP de, Lebrun M-H (2014) Resolving the polyphyletic nature of *Pyricularia* (Pyriculariaceae). Studies in Mycology 79:85–120. https://doi.org/10.1016/j.simyco.2014.09.004.

Kohlmeyer J (1966) Neue Meerespilze an Mangroven. Berichte der Deutschen Botanischen Gesellschaft 79:27–37.

Kohlmeyer J (1968a) A new *Trematosphaeria* from roots of *Rhizophora racemosa*. Mycopathologie et Mycologia Applicata 34:1–5. https://doi.org/10.1007/BF02050837.

Kohlmeyer J (1968b) Marine fungi from the tropics. Mycologia 60:252–270. https://doi.org/10.2307/3757156.

Kohlmeyer J (1969) Marine fungi of Hawaii including the new genus *Helicascus*. Canadian Journal of Botany 47:1469–1487.

Kohlmeyer J (1981) Marine fungi from Martinique. Canadian Journal of Botany 59:1314–1321.

Kohlmeyer J, Kohlmeyer E (1971) Marine fungi from tropical America and Africa. Mycologia 63:831–861. https://doi.org/10.2307/3758050.

Kolawole RM, Thomas BT, Adekunle AA, Oluwadun A (2013) Postharvest pathogenic fungi of wheat circulation in Lagos State, Nigeria. American Journal of Research Communication 1:421–428.

Koné NA, Koné D, Nicot P (2010) State of knowledge of fungal diversity in Côte d’Ivoire. In: Konate S, Kampmann D (eds) Atlas de la biodiversité de l'Afrique de l'Ouest, Tome III: Côte d'Ivoire. Goethe-Universität Frankfurt am Main, Frankfurt, pp 172–177.

Koné NA, Yéo K, Konaté S, Linsenmair KE (2013) Socio-economical aspects of the exploitation of *Termitomyces* fruit bodies in central and southern Côte d’Ivoire: Raising awareness for their sustainable use. Journal of Applied Biosciences 70:5580–5590.

Kranz J (1964a) Fungi collected in the Republic of Guinea I. Collections from the rain forest. Sydowia 17:132–138.

Kranz J (1964b) Fungi collected in the Republic of Guinea II. Collection from the Kindia area in 1962. Sydowia 17:174–185.

Kranz J (1966a) Neue *Cercospora-*Arten aus Westafrika. Sydowia 19:73–83.

Kranz J (1966b) Fungi collected in the Republic of Guinea III. Collections from the Kindia area 1963-64, and host index. Sydowia 19:92–107.

Kranz J (1967) Über parasitische Pilzgesellschaften kleinster Areale. Zeitschrift für Pflanzenkrankheiten und Pflanzenschutz / Journal of Plant Diseases and Protection 73:27–34.

Kranz J (1968) Neue Hyphomyceten aus Guinea. Sydowia 20:211–217.

Kranz J (1969) Neue Spaeropsidales aus Guinea. Sydowia 22:360–365.

Kranz J (1970) Neue *Mycosphaerella*-Arten aus Guinea. Nova Hedwigia 18:235–239.

Kreisel H (2001) Checklist of the gasteral and secotioid Basidiomycetes of Europe, Africa, and the Middle East. Österreichische Zeitschrift für Pilzkunde 10:213–313.

Kreisel H, Dring DM (1967) An emendation of the genus *Morganella* Zeller (Lycoperdaceae) with 2 plates and 3 figures. Feddes Repertorium 74:109–122. https://doi.org/10.1002/fedr.19670740105.

Kukwa M, Piątek M (2014) First records of the lichen *Septotrapelia usnica* (Lecanorales, Ascomycota) from West Africa. Polish Botanical Journal 59:105–108. https://doi.org/10.2478/pbj-2014-0006.

Kurtzman CP (2001) Four new *Candida* species from geographically diverse locations. Antonie van Leeuwenhoek 79:353–361.

Kurtzman RH Jr. (2011) The good and the bad: Fungi in Africa. Micologia Aplicada International 23:29–33.

Kutama AS, Bashir B, James D (2010) Incidence of sorghum diseases in Dawakin-Kudu Local Government Area, Kano State, Nigeria. African Journal of General Agriculture 6:307–313.

Leakey CLA (1964) *Dactuliophora*, a new genus of mycelia sterilia from tropical Africa. Transactions of the British Mycological Society 47:341–350. https://doi.org/10.1016/S0007-1536(64)80006-1.

Leather RI (1959) Diseases of economic plants in Ghana other than cacao, Bulletin No.1. Ghana Ministry of Food and Agriculture, Accra, Ghana.

Lenné JM (1990) A world list of fungal diseases of tropical pasture species. Phytopathological Papers, vol 31. CAB International, Wallingford, UK.

Lenné JM, Calderón M (1989) Problemas causados por plagas y enfermedades en *Andropogon gayanus*. In: Toledo JM, Vera R, Lascano C, Lenné JM (eds) *Andropogon gayanus* Kunth: Un pasto para los suelos ácidos del trópico. CIAT, pp 191–238.

Ling L (1953) Taxonomic notes on the Ustilaginales III. Sydowia 7:151–157.

Liyanage KK, Khan S, Mortimer PE, Hyde KD, Xu J, Brooks S, Ming Z, Sieber T (2016) Powdery mildew disease of rubber tree. Forest Pathology 46:90–103. https://doi.org/10.1111/efp.12271.

Lloyd CG (1917) Mycological notes. Mycological Notes 50:701–706.

Lockwood TF (2007) Chasing the rain: My treasure hunt for the world's most beautiful mushrooms, 1st ed. Taylor F. Lockwood, Mendocino, Calif.

Locquin N (1954) Une chanterelle comestible de la Côte d'Ivoire: *Hygrophoropsis mangenotii* sp. nov. Journal d'Agriculture Tropicale et de Botanique Appliquée 1:359–361.

Lubbe CM, Denman S, Cannon PF, Groenewald JZ, Lamprecht SC, Crous PW (2004) Characterization of *Colletotrichum* species associated with diseases of Proteaceae. Mycologia 96:1268–1279. https://doi.org/10.2307/3762144.

Luc M (1951) Champignons nouveaux du colatier en Côte d'Ivoire. Revue de Mycologie 16:107–123.

Luc M (1953a) Champignons graminicoles de Côte d’Ivoire I. - Pyrénomycètes. Revue de Mycologie, Supplément Colonial 18:1–37.

Luc M (1953b) Sur trois champignons du palmier à huile en Côte d'Ivoire. Revue de Mycologie, Supplément Colonial 18:94–102.

Lücking R (2008) Foliicolous lichenized fungi. Flora Neotropica, vol 103. The New York Botanical Garden Press, Bronx, N.Y.

Lücking R (2014) Three new species of thelotremoid Graphidaceae (lichenized Ascomycota: Ostropales) from tropical Africa. Phytotaxa 189:176–179.

Lücking R, Kalb K (2002) New species and further additions to the foliicolous lichen flora of Kenya (East Africa), including the first lichenicolous *Aulaxina* (Ostropales: Gomphillaceae). Botanical Journal of the Linnean Society 139:171–180. https://doi.org/10.1046/j.1095-8339.2002.00058.x.

Lücking R, Santesson R (2001) New species or interesting records of foliicolous lichens. VIII. Two new taxa from tropical Africa, with a key to sorediate *Fellhanera* species. The Lichenologist 33:111–116. https://doi.org/10.1006/lich.2001.0312.

Lücking R, Santesson R (2002) On the identity of *Pyrenotrichum atrocyaneum, P. mirum* and *P. podosphaera,* campylidia of lichenized Ascomycota (Lecanorales: Ectolechiaceae). The Bryologist 105:57–62.

Lücking R, Vězda A (1998) Taxonomic studies in foliicolous species of the genus *Porina* (lichenized Ascomycotina Trichotheliaceae ) *-* II. The *Porina epiphylla* group. Willdenowia 28:181–225. https://doi.org/10.3372/wi.28.2818.

Lücking R, Becker U, Follmann G (1998) Foliikole Flechten aus dem Taï-Nationalpark, Elfenbeinküste (Tropisches Afrika). II. Ökologie und Biogeografie. Herzogia 13:207–228.

Lunghini D, Onofri S (1980) *Craspedodidymum abigianense* sp. nov., a new dematiaceous hyphomycete from Ivory Coast forest litter. Transactions of the British Mycological Society 74:208–211. https://doi.org/10.1016/S0007-1536(80)80033-7.

Lunghini D, Rambelli A (1978) Ifomiceti nuovi o rari rinvenutinella foresta tropicale Africana. Giornale Botanico Italiano 112:175–195.

Lunghini D, Rambelli A (1979) *Helicoubisia* e *Talekpea,* due novi generi di fali demaziacei. Micologia Italiana 8:21–24.

Lunghini D, Rambelli A, Onofri S (1982) New *Codinaea* species from tropical forest litter. Mycotaxon 14:116–124.

Lustrati L (1980) *Cercosporella caliculata*, sp. nov. nuova specie di ifale demaziaceo. Micologia Italiana 9:11–14.

Maas Geesteranus RA, Lanquetin P (1975) Observations sur quelques champignons hydnoides de l'Afrique. Persoonia 8:145–165.

Maba DL, Guelly AK, Yorou NS, de Kesel A, Verbeken A, Agerer R (2014a) The genus *Lactarius* s. str. (Basidiomycota, Russulales) in Togo (West Africa): phylogeny and a new species described. IMA Fungus 5:39–49. https://doi.org/10.5598/imafungus.2014.05.01.05.

Maba DL, Guelly AK, Yorou NS, Verbeken A, Agerer R (2014b) Two new *Lactifluus* species (Basidiomycota, Russulales) from Fazao Malfakassa National Park (Togo, West Africa). Mycological Progress 13:513–524. https://doi.org/10.1007/s11557-013-0932-4.

Maba DL, Guelly AK, Yorou NS, Verbeken A, Agerer R (2015a) Phylogenetic and microscopic studies in the genus *Lactifluus* (Basidiomycota, Russulales) in West Africa, including the description of four new species. IMA Fungus 6:13–24. https://doi.org/10.5598/imafungus.2015.06.01.02.

Maba DL, Guelly AK, Yorou NS, Agerer R (2015b) Diversity of *Lactifluus* (Basidiomycota, Russulales) in West Africa: 5 new species described and some considerations regarding their distribution and ecology. Mycosphere 6:737–759.

Maggi O, Bartoli A, Rambelli A (1978) Two new species of *Triadelphia* from rhizosphere of *Loudetia simplex* in the Ivory Coast. Transactions of the British Mycological Society 71:148–154. https://doi.org/10.1016/S0007-1536(78)80018-7.

Maggi O, Persiani AM (1994) *Aspergillus implicatus*, a new species isolated from Ivory Coast forest soil. Mycological Research 98:869–873. https://doi.org/10.1016/S0953-7562(09)80256-6.

Maharachchikumbura SSN, Hyde KD, Groenewald JZ, Xu J, Crous PW (2014) *Pestalotiopsis* revisited. Studies in Mycology 79:121–186. https://doi.org/10.1016/j.simyco.2014.09.005.

Mahoney DP, Huang LH, Backus, MP (1969) New homothallic neurosporas from tropical soils. Mycologia 61:264–272.

Mains EB (1949) New species of *Torrubiella, Hirsutella* and *Gibellula*. Mycologia 41:303–310.

Makun, HA, Gbodi, TA, Akanya HO, Salako, EA, Ogbadu, GH (2007) Fungi and some mycotoxins contaminating rice (*Oryza sativa*) in Niger state, Nigeria. African Journal of Biotechnology 6:99–108.

Manier J-F, Gasc E, Bouix G (1972a) *Enterobryus tuzetae* n. sp. (Trichomycètes - Eccrinales) de l'intestin postérieur de *Pachybolus ligulatus* (Voges) (Diplopodes - Spirobolidae) récoltés au Dahomey (Afrique). Biologia Gabonica 8:305–322.

Manier J-F, Gasc C, Bouix G (1972b) *Mononema demangei* thallophyte de l'oesophage de *Orthomorpha coarctata* (Saussure) et de *Cordyloporus ornatus* (Peters) myriapodes polydesmides du Dahomey. Biologia Gabonica 8:323–331.

Manier J-F, Gasc C, Bouix G (1974) Sur quelques *Enterobryus* (Trichomycètes Eccrinales) parasites de myriapodes diplopodes du Sud-Dahomey. Bulletin de l'Institut Français d'Afrique Noire, Ser. A 36:614–641.

Marbach B (2000) Corticole und lignicole Arten der Flechtengattung *Buellia* sensu lato in den Subtropen und Tropen: Mit 6 Tafeln. Bibliotheca Lichenologica, Bd. 74. Cramer in der Gebr.-Borntraeger-Verl.-Buchh, Berlin.

Marley PS, Diourté M, Neya A, Nutsugah SK, Sérémé P, Katilé SO, Hess DE, Mbaye DF, Ngoko Z (2002) Sorghum and pearl millet diseases in West and Central Africa. In: Leslie JF (ed) Sorghum and millets diseases [based on contributions to the Third Global Conference on Sorghum and Millets Diseases in Guanajuato, Mexico, September 2000], 1st ed. Iowa State Press, Ames, pp 419–425.

Mason EW (1941) Annotated account of fungi received at the Imperial Mycological Institute: List II. Mycological Papers 2:101–144.

Massee GE (1899) Fungi exotici, II. Bulletin of Miscellaneous Information 1899:164–184. https://doi.org/10.2307/4111355.

Massee GE (1901) Fungi exotici, III. Bulletin of Miscellaneous Information 1901:150–169. https://doi.org/10.2307/4114928.

Massee GE (1906) Fungi exotici, V. Bulletin of Miscellaneous Information 1906:255–258. https://doi.org/10.2307/4118220.

Massee GE (1907) Fungi exotici, VI. Bulletin of Miscellaneous Information 1907:121–124. https://doi.org/10.2307/4111826.

Massee GE (1908) Fungi exotici, VIII. Bulletin of Miscellaneous Information 1908:216–219.

Massee GE (1910a) Fungi exotici, X. Bulletin of Miscellaneous Information 1910:1–6. https://doi.org/10.2307/4115015.

Massee GE (1910b) Fungi exotici, XI. Bulletin of Miscellaneous Information 1910:249–253. https://doi.org/10.2307/4111851.

Massee GE, Salmon ES (1902) Researches on coprophilous fungi. II. Annals of Botany os-16:57–94. https://doi.org/10.1093/oxfordjournals.aob.a088871.

Masuka AJ, Ryvarden L (1999) *Dichomitus* in Africa. Mycological Research 103:1126–1130. https://doi.org/10.1017/S0953756299008436.

Matzer M (1996) Lichenicolous ascomycetes with fissitunicate asci on foliicolous lichens. Mycological Papers, vol 171. CAB International, Wallingford.

Mayor E, Viennot-Bourgin G (1951) Contribution à la connaissance des micromycètes de la Côte d’Ivoire. Bulletin de la Société Mycologique de France 67:113–139.

Mayorquin JS, Wang DH, Twizeyimana M, Eskalen A (2016) Identification, distribution, and pathogenicity of Diatrypaceae and Botryosphaeriaceae associated with Citrus Branch Canker in the Southern California desert. Plant Disease 100:2402–2413. https://doi.org/10.1094/PDIS-03-16-0362-RE.

Mbogene JT, Temegene CN, Houngnandan P, Youmbi E, Tonfack LB, Ntsomboh-Ntsefong G (2015) Biodiversity of arbuscular mycorrhizal fungi of pumkins (*Cucurbita* spp.) under the influence of fertilizers in ferralitic soils of Cameroon and Benin. Journal of Applied Biology and Biotechnology 3:1–10.

Meeboon J, Hidayat I, Takamatsu S (2013) *Pseudoidium javanicum*, a new species of powdery mildew on *Acalypha* spp. from Indonesia. Mycoscience 54:183–187. https://doi.org/10.1016/j.myc.2012.08.006.

Meléndez-Howell LM (1965) Un nouveau cas de bourgenonnement et de polymorphisme sporaux chez les agarics. Revue de Mycologie 29:315–325.

Mercado Sierra A, Holubová-Jechová V, Mena Portales J (1997) Hifomicetes demaciáceos de Cuba: Enteroblásticos. Monografie, vol 23. Museo regionale di scienze naturali, Torino.

Merli S, Garofano L, Rambelli A, Pasqualetti M (1992) *Chaetopsina nimbae*, a new species of dematiaceous hyphomycetes. Mycotaxon 44:323–331.

Meswaet Y, Mangelsdorff RD, Yorou NS, Piepenbring M (2019) A new species of *Pseudocercospora* on *Encephalartos barteri* from Benin. Asian Journal of Mycology 2:101–109.

Mibey RK, Hawksworth DL (1997) Meliolaceae and Asterinaceae of the Shimba Hills, Kenya. Mycological Papers, vol 174. CAB International, Wallingford.

Millogo A, Ki-Zerbo G-A, Andonaba JB, Lankoandé D, Sawadogo A, Yaméogo AB (2004) La cryptococcose neuroméningée au cours de l'infektion par le VIH au Centre hospitalier de Bobo-Dioulasso (Burkina Faso). Bulletin de la Société de Pathologie Exotique 97:119–121.

Minter DW, Brady BL (1980) Mononematous species of *Hirsutella*. Transactions of the British Mycological Society 74:271–282. https://doi.org/10.1016/S0007-1536(80)80157-4.

Mohammed C, Guillaumin JJ (1994) *Armillaria* in tropical Africa. In: Isaac (ed) Aspects of tropical mycology. pp 207–217.

Montagne C (1845) Note sur deux nouveaux champignons du Sénégal: Botanique et biologie végétale. Annales des Sciences Naturelles Botanique 3:272–274.

Montegut J (1967) Contribution à étude d'un complexe parasitaire s'attaquant à l'appareil aérien des cotonniers et entrainant son déssèchement. Rapport de mission à l’Office du Niger (Mali). Coton et Fibres Tropicales 22:439–453.

Mordue JEM (1980a) *Pestalotiopsis dichaeta*. CMI 68:No 675.

Mordue JEM (1980b) *Pestalotiopsis mangiferae*. CMI 68:No 676.

Moreau C (1949) Micromycètes africains. I. Revue de Mycologie, Supplément Colonial 14:15–22.

Moreau C (1950a) Les maladies parasitaires des principales cultures coloniales. Revue de Mycologie, Supplément Colonial 14/15:23–40.

Moreau C (1950b) Les mycocécidies des régions tropicales. Revue de Mycologie, Supplément Colonial 15:1–44.

Moreau C (1951) Les maladies parasitaires des principales cultures coloniales: Revue bibliographique. VIII. Revue de Mycologie, Supplément Colonial 16:124–138.

Moreau C (1952) Les maladies parasitaires des principales cultures coloniales: Revue Bibliographique. X. Revue de Mycologie, Supplément Colonial 17:84–96.

Moreau C, Moreau M (1951) Pyrénomycètes du caféier en Côte d'Ivoire. Revue de Mycologie, Supplément Colonial 16:12–80.

Moreau C, Moreau M (1955a) Ascomycètes de Côte d'Ivoire I. *Chevalieropsis ctenotricha* (Pat. et Har.) Arn. Revue de Mycologie 20:48–62.

Moreau C, Moreau M (1955b) Ascomycètes de Côte d'Ivoire II. Trois ascomycètes foliicoles du *Macaranga*. Revue de Mycologie, Supplément Colonial 20:113–122.

Moreau C, Moreau M (1956) Ascomycète de Côte d'Ivoire IV. Une diaporthacée sur feuilles de *Trachyphrynium*. Revue de Mycologie, Supplément Colonial 21:119-124, 2 fig.

Moreau C, Moreau M (1959a) Champignons foliicoles de Guinée I. Ascomycètes du *Lophira alata* Banks et leur parasites. Revue de Mycologie 24:324–348.

Moreau C, Moreau M (1959b) Champignons foliicoles de Guinée II. Ascomycètes à organisation fruste. Revue de Mycologie 24:349–359.

Morris B (1990) An annotated check-list of the macrofungi of Malawi. Kirkia 13:323–364.

Mossebo DC, Amougou A, Atangana RE (2002) Contribution à l'étude du genre *Termitomyces* (Basidiomycètes) au Cameroun: écologie et systématique. Bulletin de la Société Mycologique de France 118:195–249.

Mossebo DC, Essouman EPF, Machouart MC, Gueidan C (2017) Phylogenetic relationships, taxonomic revision and new taxa of *Termitomyces* (Lyophyllaceae, Basidiomycota) inferred from combined nLSU- and mtSSU-rDNA sequences. Phytotaxa 321:71–102. https://doi.org/10.11646/phytotaxa.321.1.3.

Motyka J (1938) Lichenum generis *Usnea* studium monographicum: Pars Systematica. 1-2, Leopoli.

Mulder JL (1982) New species and combinations in *Stenella*. Transactions of the British Mycological Society 79:469–478. https://doi.org/10.1016/S0007-1536(82)80039-9.

Müller E, von Arx JA (1962) Beiträge zur Kryptogamenflora der Schweiz. Die Gattungen der didymosporen Pyrenomyceten, 11, Teil 2. Büchler, Wabern-Bern.

Müller J (1893) Lichenes Scottiani in Sierra Leone Africae occidentalis a cl. Scott-Elliot lecti et missi, quos enumerat. Bulletin de l'Herbier Boissier 1:304.

Nag Raj TR (1993) Coelomycetous anamorphs with appendage-bearing conidia. Mycologue Publ; Department of Biology Univ. of Waterloo, Waterloo Ontario.

Nag Raj TR (1995) *Lomachashaka* revisted. Mycotaxon 53:311–324.

Nicholson, RA (2000) Mushroom from the forests of South East Nigeria. The Nigerian Field 65:169–191.

Nicot J (1955) Quelques hyphomycètes corticoles de Côtes d'Ivoire. Revue de Mycologie, Supplément Colonial:123–131.

Nielsen DS, Jakobsen M, Jespersen L (2010) *Candida halmiae* sp. nov., *Geotrichum ghanense* sp. nov. and *Candida awuaii* sp. nov., isolated from Ghanaian cocoa fermentations. International Journal of Systematic and Evolutionary Microbiology 60:1460–1465. https://doi.org/10.1099/ijs.0.016006-0.

Nipa KK, Kamal AHM, Imtiaj A (2020) Prevalence and clinicomycological studies of otomycosis: A review. Journal of Bio-Science 28:121–135. https://doi.org/10.3329/jbs.v28i0.44718.

Nirenberg HI, O'Donnell KL (1998) New *Fusarium* species and combinations within the *Gibberella fujikuroi* species complex. Mycologia 90:434–458.

Nwokocha NJ, Umechuruba CI, Wokocha RC, Opara EU, Nwokocha JV (2015) Reduction of seed-borne fungi of the genus *Aspergillus* associated with egusi melon *Colocynthis citrullus* (L.) seeds using chlorine disinfectants–implications on seed germination. Journal of Agriculture and Sustainability 7:87–98.

Nyarko BJB, Adomako D, Serfor-Armah Y, Dampare SB, Adotey D, Akaho EHK (2006) Biomonitoring of atmospheric trace element deposition around an industrial town in Ghana. Radiation Physics and Chemistry 75:954–958. https://doi.org/10.1016/j.radphyschem.2005.08.021.

Ocansey BK, Pesewu GA, Codjoe FS, Osei-Djarbeng S, Feglo PK, Denning DW (2019) Estimated Burden of Serious Fungal Infections in Ghana. Journal of Fungi (Basel, Switzerland) 5:1–15.

Oehl F, Palenzuela J, Sánchez-Castro I, Hountondji F, Tchabi A, Lawouin L, Barea JM, Coyne D, Alves da Silva G (2011) *Acaulospora minuta,* a new arbuscular mycorrhizal fungal species from sub-Saharan savannas of West Africa. Journal of Applied Botany and Food Quality 84:213–218.

Oehl F, Tchabi A, Silva GA, Sánchez-Castro I, Palenzuela J, do Monte Junior IP, Lawouin L, Coyne D, Hountondji F (2014) *Acaulospora spinosissima*, a new arbuscular mycorrhizal fungus from the Southern Guinea Savanna in Benin. Sydowia 66:29–42. https://doi.org/10.12905/0380.sydowia66(1)2014-0029.

Ogundana SK (1979) Nigeria and the mushrooms. Mushroom Science 10:537–545.

Ogundana SK, Fagade OE (1982) Nutritive value of some Nigerian edible mushrooms. Food Chemistry 8:263–268. https://doi.org/10.1016/0308-8146(82)90028-0.

Ogunlana EO (1975) Fungal air spora at Ibadan, Nigeria. Applied Microbiology 29:458–463.

Okafor N (1965) Micro-organisms associated with dead insect larvae in Nigeria. Nature 208:1015–1016. https://doi.org/10.1038/2081015a0.

Okhuoya JA, Akpaja EO, Osemwegie OO, Oghenekaro AO, Ihayere CA (2010) Nigerian mushrooms: underutilized non-wood forest resources. Journal of Applied Science and Evironmental Management 14:43–54.

Okigbo R, Emeka AN (2010) Biological control of rot-inducing fungi of water yam (*Dioscorea alata*) with *Trichoderma harzianum*, *Pseudomonas syringae* and *Pseudomonas* *chlororaphis*. Journal of Stored Products and Postharvest Research 1:18–23.

Okoli I, Oyeka CA, Kwon-Chung KJ, Theelen B, Robert V, Groenewald JZ, McFadden DC, Casadevall A, Boekhout T (2007) *Cryptotrichosporon anacardii* gen. nov., sp. nov., a new trichosporonoid capsulate basidiomycetous yeast from Nigeria that is able to form melanin on niger seed agar. FEMS Yeast Research 7:339–350. https://doi.org/10.1111/j.1567-1364.2006.00164.x.

Olou BA, Ordynets A, Langer E (2019b) First new species of *Fulvifomes* (Hymenochaetales, Basidiomycota) from tropical Africa. Mycological Progress 18:1383–1393. https://doi.org/10.1007/s11557-019-01536-9.

Olou BA, Yorou NS, Striegel M, Bässler C, Krah F-S (2019) Effects of macroclimate and resource on the diversity of tropical wood-inhabiting fungi. Forest Ecology and Management 436:79–87. https://doi.org/10.1016/j.foreco.2019.01.016.

Omoifo CO (1996) Dimorphic fungi isolated from spontaneously fermented juice of soursoo, *Annona muricata* L. Hindustan antibiotics bulletin 37:1–11.

Ono Y (2015) *Kuehneola* species (Phragmidiaceae, Pucciniales) on Vitaceae plants. Mycological Progress 14:112–118. https://doi.org/10.1007/s11557-015-1076-5.

Ono Y, Hennen JF (1979) Teliospore ontogeny in *Ypsilospora baphiae* and *Y. africana* sp. nov. (Uredinales). Transactions of the British Mycological Society 73:229–233. https://doi.org/10.1016/S0007-1536(79)80106-0.

Osagualekhor DO, Okhuoya JA (2005) Sociocultural and ethnomycological uses of mushrooms among the Esan people of Nigeria. International Journal of Medicinal Mushrooms 7:442–445.

Osemwegie OO, Okhuoya JA (2009) Diversity of macrofungi in oil palm agroforests of Edo State, Nigeria. Journal of Biological Sciences 9:584–593. https://doi.org/10.3923/jbs.2009.584.593.

Osemwegie OO, Okhuoya JA (2011) Diversity and abundance of macrofungi in rubber agroforests in southwestern Nigeria. Nordic Journal of Botany 29:119–128. https://doi.org/10.1111/j.1756-1051.2010.00717.x.

Osemwegie OO, Isikhuemhen OS, Onyolu OJ, Okhuoya JA (2002) Cultivation of a selected sporophore-only-producing strain of edible and medicinal mushroom, *Pleurotus tuberregium* (Fr.) Singer (Agaricomycetideae) on waste paper and plantain peelings. International Journal of Medicinal Mushrooms 4:343–348.

Osemwegie OO, Eriyamremu, GE, Abdulmalik J (2006) A survey of macrofungi in Edo/Delta region of Nigeria, their morphology and uses. Global Journal of Pure and Applied Sciences 12:149–157.

Osemwegie OO, Oghenekaro AO, Ihayere CA, Sule E (2010a) Folk uses of mushrooms by the Akoko-Edo people in Nigeria. Asian Journal of Microbiological, Biotechnological, Envirenmental Science 12:709–714.

Osemwegie OO, Okhuoya JA, Oghenekaro AO, Evueh, GA (2010b) Macrofungi community in rubber plantations and a forest of Edo State, Nigeria. Journal of Applied Sciences 10:391–398.

Osemwegie OO, John OA, Theophilus DA (2014) Ethnomycological conspectus of West African mushrooms: An awareness document. Advances in Microbiology 4:39–54. https://doi.org/10.4236/aim.2014.41008.

Oso BA (1975) Mushrooms and the Yoruba people of Nigeria. Mycologia 67:311–319.

Oso BA (1976) *Phallus aurantiacus* from Nigeria. Mycologia 68:1076–1082. https://doi.org/10.2307/3758723.

Oso BA (1977a) Mushrooms in Yoruba mythology and medicinal practices. Economic Botany 31:367–371. https://doi.org/10.1007/BF02866888.

Oso BA (1977b) *Pleurotus tuber-regium* from Nigeria. Mycologia 69:271–279. https://doi.org/10.2307/3758652.

Osuji CN, Nwabueze EU, Akunna TO, Ahaotu EO (2013) Nutritional composition and antibacterial activity of indigenous edible mushroom *Coprinopsis atramentaria*. International Journal of Applied Sciences and Engineering 1:61–65.

Ou SH (1972) Rice diseases. Commonwealth Mycological Institute, Farnham Royal, Slough, England.

Ouoba LII, Nielsen DS, Anyogu A, Kando C, Diawara B, Jespersen L, Sutherland JP (2015) *Hanseniaspora jakobsenii* sp. nov., a yeast isolated from Bandji, a traditional palm wine of *Borassus akeassii*. International Journal of Systematic and Evolutionary Microbiology 65:3576–3579. https://doi.org/10.1099/ijsem.0.000461.

Oyetayo, OV (2011) Medicinal uses of mushrooms in Nigeria: Towards full and sustainable exploitation. African Journal of Traditional, Complementary and Alternative Medicines 8:267–274.

Oyetunji OJ, Osonubi O (2007) Assessment of influence of alley cropping system and arbuscular mycorrhizal (AM) fungi on cassava productivity in derived savanna zone of Nigeria. World Journal of Agricultural Sciences 3:489–495.

Páez de Badillo I (1982) Notas sobre el género *Heterochaete*. (I). Ernstia 13:1–36.

Pagano S, Zucconi L (1995) *Rhinocladiella pyriformis* (Fungi, Hyphomycetes), a new species from Ivory Coast. Nova Hedwigia 61:243–247.

Palisot de Beauvois AMFJ (1804) Flore d'Oware et de Bénin, en Afrique, Vol. I: 1-100 pp. Fain Jeune, Paris.

Palisot de Beauvois AMFJ (1807) Flore d'Oware et de Bénin, en Afrique, Vol. II: 1-95 pp. Fain Jeune, Paris.

Papierok B, Charpentie M-J (1982) Les champignons se développant en Côte-d'Ivoire sur la fourmi *Paltothyreus tarsatus* F. Rélation entre l'hyphomycète *Tilachlidiopsis* *catenulata* sp. nov. et l'ascomycète *Cordyceps myrmecophila* Cesati 1846. Mycotaxon 14:351–368.

Paris M (1902) Lichens de Madagascar et de l'Afrique Occidentale Francaise. Bulletin de la Société Botanique de France 49:269–273.

Pasqualetti M, Rambelli A (1999) *Dactylaria asymetrica*, a new species of mitosporic fungi from Ivory Coast forest litter. 1. Mycotaxon 72:27–31.

Patouillard N (1889) Le genre *Ganoderma.* Société Mycologique de France 5:64-80, Plates 10, 11.

Patouillard N (1896) Champignons nouveaux ou peu connus. Bulletin de la Société Mycologique de France 12:132-136, Plance XIV.

Patouillard N (1907) Quelques champignons de l´Afrique occidentale. Bulletin de la Société Mycologique de France 23:80–85.

Patouillard N, Hariot P (1900) Enumération des champignons récoltés par M.A. Chevalier au Sénégal et dans le Soudan occidental. In: Morot LM (ed) Journal de botanique. Bureau de journaux, Paris, pp 234–246.

Patouillard N, Hariot P (1912) Fungorum novorum decas quarta. Bulletin de la Société Mycologique de France 28:280–284.

Paul NC, Deng JX, Lee HB, Yu S-H (2015) Characterization and pathogenicity of *Alternaria burnsii* from seeds of *Cucurbita maxima* (Cucurbitaceae) in Bangladesh. Mycobiology 43:384–391. https://doi.org/10.5941/MYCO.2015.43.4.384.

Pegler DN (1966) Tropical African Agaricales. Persoonia 4:73–124.

Pegler DN (1968) Studies on African Agaricales I. Kew Bulletin 21:499–533.

Pegler DN (1969) Studies on African Agaricales II. Kew Bulletin 21:219–249.

Pegler DN (1977a) A preliminary agaric Flora of East Africa, 1. publ. Kew Bulletin Additional Series 6. HMSO, London.

Pegler DN (1977b) A new species of *Richoniella* (Hymenogastrales) from Ghana. Kew Bulletin 32:12.

Pegler DN (1983) The genus *Lentinus*: A world monograph. Kew Bulletin Additional Series X. Royal Botanic Gardens Kew, Norwich.

Pegler DN, Rayner RW (1969) A contribution to the agaric flora of Kenya. Kew Bulletin 23:347–412. https://doi.org/10.2307/4117177.

Pegler DN, Young TWK (1969) *Phyllogaster* gen. nov., an agaricoid member of the Secotiaceae s.str. Proceedings van de Koninklijke Nederlandse Akademie van Wetenschappen Section C 72:222–230.

Pegler DN, Young TWK (1992) Tropical species of *Psathyrella* with ornamented spores. Mycological Research 96:503–506. https://doi.org/10.1016/S0953-7562(09)81098-8.

Penney D (2009) Field guide to wildlife of the Gambia: An introduction to common flowers & animals. Siri Scientific Press, Manchester.

Penney D (2012) Field guide to wildlife of the Gambia: An introduction to common flowers & animals. 786 colour photographs, 2nd edition. Siri Scientific Press, Manchester.

Perreau J (1983) *Russula coffeata* sp. nov., d'Afrique occidentale subéquatoriale. Cryptogamie Mycologie 4:157–164.

Persiani AM, Maggi O (1987 (1986)) A new species of *Heterocephalum* from Ivory Coast soil. Transactions of the British Mycological Society 87:631–635. https://doi.org/10.1016/S0007-1536(86)80104-8.

Persiani AM, Onofri S (1982) A new genus of synnematous Hyphomycetes from tropical rain forest. Mycotaxon 15:254–260.

Petch T (1928) Notes. Transactions of the British Mycological Society 13:142–143. https://doi.org/10.1016/S0007-1536(28)80010-6.

Petch T (1939) Notes on entomogenous fungi. Transactions of the British Mycological Society 23:127–148.

Petersen RH (1975) *Ramaria* subgenus *Lentoramaria* with emphasis on North American taxa. Bibliotheca Mycologica, vol 43. Cramer, Vaduz.

Petersen RH (1981) *Ramaria* subgenus *Echinoramaria*. Bibliotheca Mycologica, vol 79. Cramer, Vaduz.

Petersen RH (2008) Species of *Xerula* from sub-Saharan Africa. Fungal Diversity 30:121–147.

Peterson SW, Jurjević Ž, Bills GF, Stchigel AM, Vega FE (2010) Genus *Hamigera*, six new species and multilocus DNA sequence based phylogeny. Mycologia 102:847–864.

Petrak F (1947) *Deightonia* n. gen., eine neue Gattung der Melanconieen. Sydowia 1:114–116.

Petrak F (1954) Über eine neue, westafrikanische Art der Gattung *Neomelanconium* Petr. Sydowia 8:51–53.

Petrak F, Deighton FC (1952) Beiträge zur Pilzflora von Sierra Leone. Sydowia 6:309–322.

Piątek M (2015) The identity of *Entyloma anadelphiae*: reclassification and redescription of leaf and stem smut infecting *Anadelphia pumila* in Guinea. Phytotaxa 192:44–49. https://doi.org/10.11646/phytotaxa.192.1.5.

Piątek M, Yorou NS (2018) *Pseudocercospora avicenniicola* on black mangrove (*Avicennia germinans*) in Benin: The first report from Africa. Forest Pathology 49:e12478 (1-4). https://doi.org/10.1111/efp.12478.

Piątek M, Piątek J, Yorou NS (2014) Emended description and geographical distribution of *Sporisorium elegantis* (Ustilaginaceae), a species shared between West Africa and India. Phytotaxa 175:148–154. https://doi.org/10.11646/phytotaxa.175.3.4.

Piątek M, Lutz M, Yorou NS (2015) A molecular phylogenetic framework for *Anthracocystis* (Ustilaginales), including five new combinations (inter alia for the asexual *Pseudozyma flocculosa*), and description of *Anthracocystis grodzinskae* sp. nov. Mycological Progress 14:88 (1-15). https://doi.org/10.1007/s11557-015-1114-3.

Piątek M, Riess K, Karasiński D, Yorou NS, Lutz M (2016) Integrative analysis of the West African *Ceraceosorus* *africanus* sp. nov. provides insights into the diversity, biogeography, and evolution of the enigmatic Ceraceosorales (Fungi - Ustilaginomycotina). Organisms Diversity & Evolution 16:743–760. https://doi.org/10.1007/s13127-016-0285-3.

Piening LJ (1962) A check list of fungi recorded from Ghana: Part I. Ghana Ministry of Agriculture, Bulletin 2:1–92.

Piepenbring M (2000) The species of *Cintractia* s. l. (Ustilaginales, Basidiomycota). Nova Hedwigia 70:289–372.

Piepenbring M, Nold F, Trampe T, Kirschner R (2012) Revision of the genus *Graphiola* (Exobasidiales, Basidiomycota). Nova Hedwigia 94:67–96. https://doi.org/10.1127/0029-5035/2012/0094-0067.

Pirozynski KA (1962) *Circinotrichum* and *Gyrothrix*. Mycological Papers 84:1–28.

Pirozynski KA (1965) African species of *Uncinula*. Mycological Papers 101:2–23.

Punithalingam E (1969) New species of *Monochaetiella* and *Septoria*. Transactions of the British Mycological Society 53:311–315. https://doi.org/10.1016/S0007-1536(69)80069-0.

Punithalingam E (1970) Studies on Sphaeropsidales in culture. Mycological Papers 119:1–24.

Punithalingam E (1981a) New microfungi from cereals and grasses. II. Nova Hedwigia 34:67–96.

Punithalingam E (1981b) Studies on Sphaeropsidales in culture. III. Mycological Papers 149:1-42 and plates.

Raboin L-M, Selvi A, Oliveira KM, Paulet F, Calatayud C, Zapater M-F, Brottier P, Luzaran R, Garsmeur O, Carlier J, D'Hont A (2007) Evidence for the dispersal of a unique lineage from Asia to America and Africa in the sugarcane fungal pathogen *Ustilago scitaminea*. Fungal Genetics and Biology 44:64–76. https://doi.org/10.1016/j.fgb.2006.07.004.

Raciborski M (1900) Parasitische Algen und Pilze Java's. II. Teil. Bibliotheca Mycologica 37:1–46.

Rambelli A, Bartoli A (1978) *Guedea*, a new genus of dematiaceous hyphomycetes. Transactions of the British Mycological Society 71:340–342. https://doi.org/10.1016/S0007-1536(78)80121-1.

Rambelli A, Ciccarone C (1987 (1985)) Two new dematiaceous hyphomycetes from humid tropic forest litter. Giornale Botanico Italiano 119:291–294.

Rambelli A, Lunghini D (1976) *Chaetopsina ivoriensis,* a new species of dematiaceous hyphomycetes. Giornale Botanico Italiano 110:253–258.

Rambelli A, Lunghini D (1979) *Chaetopsina* species from tropical forest litter. Transactions of the British Mycological Society 72:491–494.

Rambelli A, Onofri S (1987) New species of *Kylindria* and *Xenokylindria* and notes on *Cylindrotrichum* (Hyphomycetes). Transactions of the British Mycological Society 88:393–397. https://doi.org/10.1016/S0007-1536(87)80012-8.

Rambelli A, Onofri S, Lunghini D (1981) New dematiaceous hyphomycetes from Ivory Coast forest litter. Transactions of the British Mycological Society 76:53–58.

Rammeloo J (1978) *Hemitrichia rubrobrunnea*, a new myxomycete from Sierra Leone. Bulletin du Jardin Botanique National de Belgique / Bulletin van de National Plantentuin van België 48:383–386. https://doi.org/10.2307/3667937.

Rammeloo J (1983) Combinaisons et taxons nouveaux de Gyrodontaceae (Boletineae). Bulletin du Jardin Botanique National de Belgique / Bulletin van de National Plantentuin van België 53:294–297.

Rammeloo J, Walleyn R (1993) The edible fungi of Africa, south of the Sahara: A literature survey. Scripta Botanica Belgica, vol. 5. National Botanic Garden of Belgium, Meise.

Rao V, Sutton BC (1975) Synnematous fungi I. Kavaka 3:21–28.

Raper KB, Fennell DI (1952) Two noteworthy fungi from Liberian soil. American Journal of Botany 39:79–86. https://doi.org/10.2307/2438097.

Reddy, PP (2015) Plant protection in tropical root and tuber crops. Springer, New Delhi.

Redhead JF (1968) *Inocybe* sp. associated with ectotrophic micorrhiza on *Afzelia bella* in Nigeria. The Commonwealth Forestry Review 47:63–65.

Reid DA (1975a) A new species of *Lindtneria* (Basidiomycetes) from West Africa. Kew Bulletin 30:597–600. https://doi.org/10.2307/4102901.

Reid DA (1975b) (title not found) *Heteroporus roseus, Microporellus defibulatus*. Microscopy 32:arround 449 and 452. n.v.

Resplandy R, Chevaugeon J, Delassus M, Luc M (1954) Première liste annotée de champignons parasites de plantes cultivées en Côte d'Ivoire. Annales de Epiphytes 1:1–61.

Richardson MJ (1990) An annotated list of seed-borne disease, 4. Edition. International Seet Testing Association, Zürich.

Riviere T, Diédhiou AG, Diabaté M, Senthilarasu G, Natarajan K, Verbeken A, Buyck B, Dreyfus B, Béna G, Bâ AM (2007) Genetic diversity of ectomycorrhizal Basidiomycetes from African and Indian tropical rain forests. Mycorrhiza 17:415–428. https://doi.org/10.1007/s00572-007-0117-6.

Roberts P (2000) Corticioid fungi from Korup National Park, Cameroon. Kew Bulletin 55:803–842. https://doi.org/10.2307/4113628.

Roberts P (2001) Heterobasidiomycetes from Korup National Park, Cameroon. Kew Bulletin 56:163–187.

Rodríguez Justavino D, Kirschner R, Piepenbring M (2015) New species and new records of Meliolaceae from Panama. Fungal Diversity 70:73–84. https://doi.org/10.1007/s13225-014-0292-7.

Rodriguez K, Stchigel AM, Guarro J (2002) Three new species of *Chaetomium* from soil. Mycologia 94:116–126. https://doi.org/10.2307/3761851.

Roger L (1951) Phytopathologie des pays chauds. Tome I. Encyclopédie Mycologique, XVII, Paris.

Roger L (1953) Phytopathologie des pay chauds. Tome II. Encyclopédie Mycologique, XVIII, Paris.

Roger ML (1936) Quelques champignons exotiques nouveaux ou peu connus II. Bulletin Trimestriel de la Société Mycologique de France 52:80–84.

Romagnesi H, Gilles G (1979) Les rhodophylles des forêts côtières du Gabon et de la Côte d'Ivoire: Avec une introduction générale sur la taxonomie du genre. Beihefte zur Nova Hedwigia, Heft 59. J. Cramer, Vaduz.

Rossi W (1978) Due nuove laboulbeniali della Sierra Leone (Ascomycetes). Natura: Rivista di Scienze Naturali 69:17–22.

Rossi W (1982) Laboulbeniali della Sierra Leone (Ascomycetes). Accademia Nazionale dei Lincei 255:9–22.

Rossi W (1986) Su alcune specie di *Laboulbenia* nuove o interessanti della Sierra Leone. Accademia Nazionale dei Lincei 260:77–96.

Rossi W (1988) New or interesting Laboulbeniales (Ascomycetes) parasitic on Diptera. Webbia 42:170–178.

Rossi W (1990) New or interesting Laboulbeniales (Ascomycetes) from Sierra Leone. Quaderni dell'Accademia Nazionale dei Lincei 265:5–13.

Rossi W (1994) A new contribution to the knowledge of the Laboulbeniales (Ascomycetes) from Sierra Leone. Quaderni dell'Accademia Nazionale dei Lincei 267:5–17.

Rossi W, Blackwell M (1986) New species of *Laboulbenia* from termite hosts in Africa. Mycologia 78:142–145.

Rossi W, Blackwell M (1990) Fungi associated with African earwigs and their relationship to South American forms. Mycologia 82:138–140. https://doi.org/10.2307/3759975.

Rossi W, Cesari Rossi MG (1979) Trois laboulbéniales (Ascomycetes) nouvelles, parasites de diptères. Canadian Journal of Botany 57:993–996.

Rossi W, Leonardi M (2013) New species of *Stigmatomyces* (Laboulbeniomycetes) from Sierra Leone. Plant Biosystems 147:79–83. https://doi.org/10.1080/11263504.2012.695297.

Ryvarden L (2004) Studies in neotropical polypores 19. Two wood-inhabiting *Amauroderma* species. Synopsis Fungorum 18:57–61.

Saccardo PA, Trotter A (eds) (1913) Sylloge fungorum omnium hucusque cognitorum: Supplementum universale, vol 22. sumptibus auctoris

Salisbury G (1971) The thelotremata of Angola and Mocambique. Revista de Biologia Lisboa 7:271–280.

Samson RA (1974) *Paecilomyces* and some allied hyphomycetes. Studies in Mycology 6:1–119.

Samson RA, Evans HC (1973) Notes on entomogenous fungi from Ghana. I. The genera *Gibellula* and *Pseudogibellula*. Acta Botanica Neerlandica 22:522–528. https://doi.org/10.1111/j.1438-8677.1973.tb00873.x.

Samson RA, Evans HC (1974) Notes on entomogenous fungi from Ghana. II. The genus *Acanthomyces*. Acta Botanica Neerlandica 23:28–35.

Samson RA, Evans HC (1975) Notes on entomogenous fungi from Ghana. III. The genus *Hymenostilbe*. Proceedings van de Koninklijke Nederlandse Akademie van Wetenschappen Section C 78:73–80.

Samson RA, Evans HC (1977) Notes on entomogenous fungi from Ghana. IV. The genera *Paecilomyces* and *Nomuraea.* Proceedings van de Koninklijke Nederlandse Akademie van Wetenschappen Section C 80:128–134.

Samson RA, Evans HC (1982) *Clathroconium*, a new helicosporous hyphomycete genus from spiders. Canadian Journal of Botany 60:1577–1580.

Samson RA, Pitt JI (eds) (1986) Advances in *Penicillium* and *Aspergillus* systematics: Proceedings. NATO Advanced Science Institutes series. Series A. Life sciences, Vol. 102. Plenum Press, New York, London.

Samson RA, Evans HC, van de Klashorst G (1981) Notes on entomogenous fungi from Ghana. V. The genera *Stilbella* and *Polycephalomyces*. Proceedings van de Koninklijke Nederlandse Akademie van Wetenschappen Section C 84:289–301.

Samson RA, Evans HC, Hoekstra ES (1982) Notes on entomogenous fungi from Ghana. VI. Proceedings van de Koninklijke Nederlandse Akademie van Wetenschappen Section C 85:589–605.

Samson RA, van Reenen-Hoekstra, ES, Evans HC (1989) New species of *Torrubiella* (Ascomycotina: Clavicipitales) on insects from Ghana. Studies in Mycology 31:123–132.

Samuels GJ, Ismaiel A, Souza J de, Chaverri P (2012) *Trichoderma stromaticum* and its overseas relatives. Mycological Progress 11:215–254. https://doi.org/10.1007/s11557-011-0743-4.

Sanginga N, Carsky RJ, Dashiell K (1999) Arbuscular mycorrhizal fungi respond to rhizobial inoculation and cropping systems in farmers' fields in the Guinea savanna. Biology and Fertility of Soils 30:179–186. https://doi.org/10.1007/s003740050606.

Sankaran KV, Sutton BC (1991) *Compsosporiella deightonii* gen. et sp. nov. (Coelomycetes) from Sierra Leone. Mycological Research 95:1289–1293. https://doi.org/10.1016/S0953-7562(09)80576-5.

Sanon E, Guissou KML, Yorou NS, Buyck B (2014) Le genre *Russula* au Burkina Faso (Afrique de l'Ouest): Quelques espèces nouvelles de couleur brunâtre. Cryptogamie Mycologie 35:377–397. https://doi.org/10.7872/crym.v35.iss4.2014.377.

Sanon KB, Bâ AM, Dexheimer J (1997) Mycorrhizal status of some fungi fruiting beneath indigenous trees in Burkina Faso. Forest Ecology and Management 98:61–69. https://doi.org/10.1016/S0378-1127(97)00089-3.

Sanon KB, Bâ AM, Delaruelle C, Duponnois R, Martin F (2009a) Morphological and molecular analyses in *Scleroderma* species associated with some caesalpinioid legumes, Dipterocarpaceae and Phyllanthaceae trees in southern Burkina Faso. Mycorrhiza 19:571–584. https://doi.org/10.1007/s00572-009-0272-z.

Sanon KB, Dianda M, Guissou T, Bâ AM (2009b) Description des champignons ectomycorhiziens du genre *Scleroderma* de quelques formations forestieres du Burkina Faso. Cameroon Journal of Experimental Biology 5:69–78.

Santamaria S, Faille A (2009) New species of *Laboulbenia* and *Rhachomyces* (Laboulbeniales, Ascomycota), some of them polymorphic, parasitic on termiticolous ground beetles from tropical Africa. Nova Hedwigia 89:97–120.

Santesson R (1952) Foliicolous lichens - I. A revision of the taxonomy of the obligately foliicolous, lichenized fungi. Symbolae Botanicae Upsalienses, vol 12. A.-B. Lundequistska Bokhandeln, Uppsala.

Santesson R, Lücking R (1999) Additions to the foliicolous lichen flora of the Ivory Coast and Guinea (Tropical West Africa). Nordic Journal of Botany 19:719–734. https://doi.org/10.1111/j.1756-1051.1999.tb00681.x.

Santesson R, Tibell L (1988) Foliicolous lichens from Australia. Austrobaileya 2:529–545.

Sarr P, Ndiaye M'B, Groenewald JZ, Crous PW (2014) Genetic diversity in *Macrophomina phaseolina,* the causal agent of charcoal rot. Phytopathologia Mediterranea 53:250–268.

Savary S, Bosc J-P, Noirot M, Zadoks J (1988) Peanut rust in West Africa: A new component in a multiple pathosystem. Plant Disease 72:1001–1009. https://doi.org/10.1094/PD-72-1001.

Sawadogo A, Cayrol J-C (1990) *Dactylaria sahelensis,* une nouvelle espèce de champignon nematophage prédatrice et parasite. Riviera Scientifique 1:27–35.

Scholz H (1981) Die Gramineen- und Cyperaceen-Brandpilze (Ustilaginales) aus Togo. Willdenowia 11:101–113.

Schreurs J (1972) Black thread disease, control measures and yield stimulation in *Hevea brasiliensis* in Liberia. Doctoral dissertation. H. Veenman & Zonen N. V., Wageningen.

Segretain G, Baylet J, Darasse H, Caimain R (1959) Le *Ptosphaeria senegalensis* n. sp. agent de mycétome à grains noire. Comptes rendus hebdomadaires des séances de l'Académie des Sciences 248:3730–3732.

Seifert KA (1985) A monograph of *Stilbella* and some allied hyphomycetes. Studies in Mycology, vol 27. Centraalbureau voor Schimmelcultures, Baarn.

Séré Y, Onasanya A, Afolabi A, Mignouna, HD, Akator K (2007) Genetic diversity of the blast fungus, *Magnaporthe grisea* (Hebert) Barr, in Burkina Faso. African Journal of Biotechnology 6:2568–2577. https://doi.org/10.5897/AJB2007.000-2410.

Sérusiaux E (1984) New species or interesting records of foliicolous lichens. Mycotaxon 20:283–306.

Sérusaux E, Lücking R, Sparrius, LB (2008) *Opegrapha viridistellata* (Roccellaceae), a new foliicolous lichen species from the paleotropics. Mycotaxon 104:223–227.

Séry DJ-M, Kouadjo ZGC, Voko BRR, Zézé A (2016) Selecting native arbuscular mycorrhizal fungi to promote cassava growth and increase yield under field conditions. Frontiers in Microbiology 7:2063 (pages 1–13). https://doi.org/10.3389/fmicb.2016.02063.

Shah PA, Godonou I, Gbongboui C, Lomer CJ (1994) Natural levels of fungal infections in grasshoppers in Northern Benin. Biocontrol Science and Technology 4:331–341. https://doi.org/10.1080/09583159409355341.

Shen S, Goodwin P, Hsiang T (2001) Hemibiotrophic infection and identity of the fungus, *Colletotrichum destructivum*, causing anthracnose of tobacco. Mycological Research 105:1340–1347. https://doi.org/10.1017/S0953756201005111.

Sherwood MA (1977) The ostropalean fungi. I. Mycotaxon 5:1–277.

Sherwood MA (1980) Taxonomic studies in the Phacidiales: The genus *Coccomyces* (Rhytismataceae). Occasional papers of the Farlow Herbarium of Cryptogamic Botany, vol 15, Cambridge, Massachusetts.

Sikirou R, Ezin V, Beed F, Etchiha Afoha, S A P, Tosso FD, Ouessou Idrissou F (2015) Geographical distribution and prevalence of the main tomato fungal wilt diseases in Benin. International Journal of Biological and Chemical Sciences 9:603–613. https://doi.org/10.4314/ijbcs.v9i2.3.

Simmons EG (2000) *Alternaria* themes and variations (244-286) species on Solanaceae. Mycotaxon 75:1–115.

Simmons EG (2007) *Alternaria*: An identification manual fully illustrated and with catalogue raisonné 1796 - 2007. CBS biodiversity series, vol 6. Centraalbureau voor Schimmelcultures, Utrecht.

Singer R (1944) New genera of fungi. Mycologia 36:358–368. https://doi.org/10.2307/3754752.

Singer R (1945a) The Boletineae of Florida with notes on extralimital species I: The Strobilomycetaceae. Farlowia 2:97–141.

Singer R (1945b) The Boletineae of Florida with notes on extralimital species II: The Boletaceae (Gyroporoideae). Farlowia 2:223–303.

Singer R (1945c) The *Laschia*-complex (Basidiomycetes). Lloydia 8:170–230.

Singer R (1947) The Boletoideae of Florida with notes on extralimital species III. The American Midland Naturalist 37:1–135.

Singer R (1948a) New and interesting species of Basidiomycetes II. Papers of the Michigan Academy of Science, Arts and Letters 32:103–150.

Singer R (1948b) Diagnoses fungorum novorum agaricalium. Sydowia 2:26–42.

Singh N (1972) *Pyramidospora constricta* sp. nov., a new aquatic hyphomycete. Transactions of the British Mycological Society 59:336–339. https://doi.org/10.1016/S0007-1536(72)80027-5.

Singh N (1976) *Pyramidospora herculiformis* sp. nov., a new aquatic hyphomycete from Sierra Leone. Transactions of the British Mycological Society 66:347–350. https://doi.org/10.1016/S0007-1536(76)80070-8.

Sivanesan A (1970) *Parmulariopsella burseracearum* gen. et sp. nov. and *Microcyclus placodisci* sp. nov. Transactions of the British Mycological Society 55:509–514. https://doi.org/10.1016/S0007-1536(70)80079-1.

Sivanesan A (1973a) New species of *Vizella* on *Pycnanthus*. Transactions of the British Mycological Society 60:586–588. https://doi.org/10.1016/S0007-1536(73)80045-2.

Sivanesan A (1973b) *Gibbera anthonothae* sp. nov. and *Gibbera bolusiellae* sp. nov. Transactions of the British Mycological Society 61:396–399. https://doi.org/10.1016/S0007-1536(73)80166-4.

Sivanesan A (1974a) Two new genera of Coronophorales with descriptions and key. Transactions of the British Mycological Society 62:35–43. https://doi.org/10.1016/S0007-1536(74)80003-3.

Sivanesan A (1974b) Two new species of *Coccostroma*. Transactions of the British Mycological Society 62:422–424. https://doi.org/10.1016/S0007-1536(74)80054-9.

Sivanesan A (1975) New ascomycetes and some revisions. Transactions of the British Mycological Society 65:19-27. https://doi.org/10.1016/S0007-1536(75)80177-X.

Sivanesan A (1978) *Lasiobertia africana* gen. et sp. nov. and a new variety of *Bertia moriformis*. Transactions of the British Mycological Society 70:383–387. https://doi.org/10.1016/S0007-1536(78)80136-3.

Sivanesan A (1979) *Mycosphaerella sieberiana* sp. nov. with a *Pseudocercospora* conidial state. Transactions of the British Mycological Society 72:157–161.

Sivanesan A (1984) New species of *Exserohilum*. Transactions of the British Mycological Society 83:319–329. https://doi.org/10.1016/S0007-1536(84)80154-0.

Sivanesan A (1985) New species of *Bipolaris*. Transactions of the British Mycological Society 84:403–421. https://doi.org/10.1016/S0007-1536(85)80003-6.

Sivanesan A (1987) Graminicolous species of *Bipolaris, Curvularia, Drechslera*, *Exserohilum* and their teleomorphs. Mycological Papers, vol 158. C A B International Mycological Institute, Wallingford, Oxon.

Sivanesan A, Okpala EU (1979) New ascomycetes from Nigeria. Transactions of the British Mycological Society 72:520–524. https://doi.org/10.1016/S0007-1536(79)80173-4.

Smith G (1956) Some new species of soil moulds. Transactions of the British Mycological Society 39:111–114. https://doi.org/10.1016/S0007-1536(56)80059-4.

Spegazzini C (1915) Laboulbeniali ritrovate nelle collezioni di alcuni musei italiani. Annales del Museo Nacional de Historia Natural Buenos Aires 26:451–511.

Stadler M, Fournier J, Gardt S, Peršoh D (2010) The phylogenetic position of *Rhopalostroma* as inferred from a polythetic approach. Persoonia 25:11–21. https://doi.org/10.3767/003158510X524231.

Staiger B, Kalb K (1995) *Haematomma-*Studien: I. Die Flechtengattung *Haematomma*. Bibliotheca Lichenologica, vol 59. Cramer, Berlin u.a.

Stchigel AM, Cano J, Guarro J, Gugnani HC (2000) An new *Apiosordaria* from Nigeria, with a key to the soil-borne species. Mycologia 92:1206–1209. https://doi.org/10.2307/3761487.

Stevens, GN (1983) Tropical-subtropical Ramalinae in the *Ramalina farinacea* complex. The Lichenologist 15:213–229.

Steyaert RL (1949) Contribution à l'étude monographique de *Pestalotia* de Not. et *Monochaetia* Sacc. (*Truncatella* gen. nov. et *Pestalotiopsis* gen. nov.). Bulletin du Jardin botanique de l'État à Bruxelles 19:285–354.

Steyaert RL (1953a) New and old species of *Pestalotiopsis*. Transactions of the British Mycological Society 36:81–89.

Steyaert RL (1953b) *Pestalotiopsis* from the Gold Coast and Togoland. Transactions of the British Mycological Society 36:235–242. https://doi.org/10.1016/S0007-1536(53)80008-2.

Stolk A (1968) Studies on the genus *Eupenicillium* Ludwig III. Four new species of *Eupenicillium*. Antonie van Leeuwenhoek 34:37–53.

Strong RP, Shattuck GC (1930) Medical and pathological investigations in Liberis and the Belgian Congo: Infectious diseases. In: Strong, RP (ed) The African Republic of Liberia and the Belgian Congo: based on the observations made and material collected during the Harvard African Expedition 1926–27. Harvard University Press, Harvard, pp 210–230.

Subramanian CV (1972) *Padixonia*, a new genus of hyphomycetes. Current Science 41:282–283.

Sutton BC (1963) Two new species of *Coryneum* Nees ex Fries. Kew Bulletin 17:309–314.

Sutton BC (1964) Coelomycetes III. Mycological Papers 97:1–42.

Sutton BC (1968) *Kellermania* and its generic segregates. Canadian Journal of Botany 46:181–196.

Sutton BC (1969a) Type studies of *Coniella, Anthasthoopa* and *Cyclodomella*. Canadian Journal of Botany 47:603–608.

Sutton BC (1969b) *Minimidochium setosum* n. gen., n. sp. and *Dinemasporium aberrans* n. sp. from West Africa. Canadian Journal of Botany 47:2095–2100.

Sutton BC (1971) Coelomycetes IV. The genus *Harknessia* and similar fungi on *Eucalyptus*. Mycological Papers 123:1–46.

Sutton BC (1980) The Coelomycetes: Fungi imperfecti with pycnidia, acervuli, and stromata. Commonwealth Mycological Institute, Kew, Surrey, England.

Sutton BC, Sellar PW (1966) *Toxosporiopsis* n. gen. an unusual member of the Melanconiales. Canadian Journal of Botany 44:1505–1513.

Sutton BC, Pirozynski KA, Deighton FC (1972) *Microdochium* Syd. Canadian Journal of Botany 50:1899–1907.

Swinscow TD, Krog H (1975) Further observations on *Pyxine* in Africa. Norwegian Journal of Botany 22:125–128.

Swinscow TD, Krog H (1988) Macrolichens of East Africa. British Museum (Natural History), London.

Sydow H (1937) Novae fungorum species XXV. Annales Mycologici 35:244–286.

Sydow H (1938) Novae fungorum species XXVI. Annales Mycologici 36:156–197.

Sydow H (1939) Novae fungorum species XXVII. Annales Mycologici 37:195–253.

Sydow H, Sydow P (1904) Novae fungorum species. Annales Mycologici 2:162–174.

Talbot PHB (1951) Studies of some South African resupinate hymenomycetes. Bothalia 6:1–116.

Talbot PHB (1956) The cyphelloid fungi of South Africa. Bothalia 6:465–487.

Tam LTT, Hoai HT, Thao LP, Huong NT, Khue NM (2015) First report of *Microidium phyllanthi* causing powdery mildew on chamber bitter in Vietnam. New Disease Reports 32:32. https://doi.org/10.5197/j.2044-0588.2015.032.032.

Tchabi A, Coyne D, Hountondji F, Lawouin L, Wiemken A, Oehl F (2008) Arbuscular mycorrhizal fungal communities in sub-saharan savannas of Benin, West Africa, as affected by agricultural land use intensity and ecological zone. Mycorrhiza 18:181–195. https://doi.org/10.1007/s00572-008-0171-8.

Tchabi A, Hountondji F, Laouwin L, Coyne D, Oehl F (2009a) *Racocetra beninensis* from sub-saharan savannas: A new species in the Glomeromycetes with ornamented spores. Mycotaxon 110:199–209. https://doi.org/10.5248/110.199.

Tchabi A, Burger S, Coyne D, Hountondji F, Lawouin L, Wiemken A, Oehl F (2009b) Promiscuous arbuscular mycorrhizal symbiosis of yam (*Dioscorea* spp.), a key staple crop in West Africa. Mycorrhiza 19:375–392. https://doi.org/10.1007/s00572-009-0241-6.

Tehler A (1986) *Dirina paradoxa* (Fée) Tehler, the correct name for *Dirina approximata* Zahlbr. The Lichenologist 18:295–296.

Thoen D, Bâ AM (1989) Ectomycorrhizas and putative ectomycorrhizal fungi of *Afzelia africana* Sm. and *Uapaca guineensis* Mull. Arg. in southern Senegal. New Phytologist 113:549–559.

Thoen D, Ducousso M (1989) Champignons et ectomycorrhizes du Fouta Djalon. Revue Bois et Forêts des Tropiques 221:45–63.

Thomas MD (1991) Development of Gray Leaf Spot on *Sorghum* in Burkina Faso. Plant Disease 75:45–47.

Thomas MD, Mayango D, Oberly W (1985) Suppression and elimination of *Rhynchosporium oryzae* by benomyl in rice foliage and seed in Liberia. Plant Disease 69:884–886.

Thomas RJ (1987a) Distribution of *Termitomyces* Heim and other fungi in the nests and major workers of *Macrotermes bellicosus* (Smeathman) in Nigeria. Soil Biology and Biochemistry 19:329–333.

Thomas RJ (1987b) Distribution of *Termitomyces* and other fungi in the nests and major workers of several Nigerian Macrotermitinae. Soil Biology and Biochemistry 19:335–341. https://doi.org/10.1016/0038-0717(87)90019-8.

Thorold CA (1952) The epiphytes of *Theobroma cacao* in Nigeria in relation to the incidence of black-pod disease (*Phytophthora palmivora*). Journal of Ecology 40:125–142. https://doi.org/10.2307/2258025.

Thouvenel JC, Fauquet C (1980) *Polymyxa graminis* on new *Sorghum* species in Africa. Plant Disease 64:957–958.

Tibell L (1981) Notes on Caliciales III. Some species from Africa. The Lichenologist 13:161–165.

Tkalčec Z, Mešić A, Čerkez M (2010) *Galerella nigeriensis* (Agaricales), a new species from tropical Africa. Mycotaxon 114:263–270. https://doi.org/10.5248/114.263.

Tovide N, Adéoti K, Noumavo, Pacôme, A., Garba K, Ohin B, Soninhekpon A, Tchobo F, Gandonou C, Toukourou F, Baba-Mussa F (2018) Occurrence of molds and identification of mycoflora contaminating millet and sorghum produced and consumed in Benin. International Journal of Current Microbiology and Applied Sciences 7:3750–3763.

Tuovila H, Cobbinah JR, Rikkinen J (2011) *Chaenothecopsis khayensis*, a new resinicolous calicioid fungus on African mahogany. Mycologia 103:610–615.

Turnbull E, Watling R (1999) Some records of *Termitomyces* from old world rainforests. Kew Bulletin 54:731–738.

Turner BC, Fairfield A (1990) A putative fifth heterothallic species in *Neurospora*. Fungal Genetics Newsletter, Kansas City 37:46.

Turner PD (1971) Micro-organisms associated with oil palm (*Elaeis guineensis* Jaco.). Phytopathological Papers 14:1–58.

Tuzet O, Manier J-F (1957) Troisième contribution à la connaissance des Eccrinida commensaux de l'intestin postérieur de myriapodes diplopodes du Brésil. Révision des Eccrinida déja identifiés chez les diplopodes. Archives de Zoologie Expérimentale et Géneralé 94:121–147.

Tuzet O, Manier J-F, Vogeli-Zuber M (1952) Sur quelques parasites intestinaux de *Mardonius piceus* Attems 1952, myriapode-diplopode de Daloa (Côte d'Ivoire). Bulletin de l'Institut Français d'Afrique Noire, Ser. A 14:1143–1151.

Vainio A (1901) Lichenes. In: Natural History (ed) Catalogue of the African plants collected by Dr. Friedrich Welwitsch in 1853-61. Printed by order of the Trustees, pp 396–463.

Valenzuela-Lopez N, Cano-Lira JF, Guarro J, Sutton DA, Wiederhold N, Crous PW, Stchigel AM (2018) Coelomycetous Dothideomycetes with emphasis on the families Cucurbitariaceae and Didymellaceae. Studies in Mycology 90:1–69. https://doi.org/10.1016/j.simyco.2017.11.003.

van de Putte, de Kesel A, Nuytinck J, Verbeken A (2009) A new *Lactarius* species from Togo with an isolated phylogenetic position. Cryptogamie Mycologie 30:39–44.

van der Aa HA (1983) A new species of *Coniella*. Proceedings van de Koninklijke Nederlandse Akademie van Wetenschappen Section C 86:121–125.

van der Westhuizen GCA, Eicker A (1990) Species of *Termitomyces* occurring in South Africa. Mycological Research 94:923–937. https://doi.org/10.1016/S0953-7562(09)81306-3.

van Rooij P, de Kesel A, Verbeken A (2003) Studies in tropical African *Lactarius* species (Russulales, Basidiomycota) 11. Records from Benin. Nova Hedwigia 77:221–251. https://doi.org/10.1127/0029-5035/2003/0076-0221.

Vandemeulebroucke E, Mounkassa B, Loye de J, Jousserand P, Foujade F, Petithory, JC (1999) Teignes du cuir chevelu en milieu scolaire rural au Mali. Journal de Mycologie Médicale 9:111–113.

Vanie-Léabo LPL, Yorou NS, N´Golo AK, Francois N’GK, de Kesel A, Daouda K (2017) Diversity of ectomycorrhizal fungal fruit bodies in Como National Park, a biosphere reserve and world heritage in Côte d'Ivoire (West Africa). International Journal of Biodiversity and Conservation 9:27–44. https://doi.org/10.5897/IJBC2016.0999.

Vánky K (1997) Taxonomical studies on Ustilaginales - XVI. Mycotaxon 65:133–158.

Vánky K (2003) Taxonomical studies on Ustilaginales - XXIII. Mycotaxon 85:1–65.

Vánky K, Piątek M (2006) The genus *Testicularia* (Ustilaginomycetes). Mycologia Balcanica 3:163–167.

Vánky K, Vánky C, Denchev CM (2011) Smut fungi in Africa - a checklist. Mycologia Balcanica 8:1–77.

Verbeken A (1995) Studies in tropical African *Lactarius* species 1: *Lactarius gymnocarpus* R. Heim ex Singer and allied species. Mycotaxon 55:515–542.

Verbeken A (2010) Monograph of *Lactarius* in tropical Africa. Fungus flora of tropical Africa, vol 2. National Botanical Garden of Belgium, Meise.

Verbeken A, Buyck B (2002) Diversity and ecology of tropical ectomycorrhizal fungi in Africa. In: Watling R (ed) Macromycetes. CABI Publ, Wallingford, pp 11–24.

Vězda A (1973) Foliicole Flechten aus der Republik Guinea (W.-Afrika). Acta Musei Silesiae, Serie A 22:67–90.

Vězda A (1974) Foliicole Flechten aus der Republik Guinea (W-Afrika). II. Acta Musei Silesiae, Serie A 23:173–190.

Vězda A (1975) Foliicole Flechten aus der Republik Guinea (W-Afrika). III. Acta Musei Silesiae, Serie A 24:117–126.

Vězda A (1979) Flechtensystematische Studien XI. Beiträge zur Kenntnis der Familie Asterothyriaceae (Discolichenes). Folia Geobotanica & Phytotaxonomica 14:43–94.

Vězda A (1987) Foliicole Flechten aus Zaire (III) Die Gattung *Byssoloma* Trevisan. Folia Geobotanica & Phytotaxonomica 22:71–83.

Viennot-Bourgin G (1950) Urédinées d'Afrique. Revue de Mycologie, Supplément Colonial 15:99–105.

Viennot-Bourgin G (1951) Ustilaginales d'Afrique (première note). Revue de Mycologie, Supplément Colonial 16:101–106.

Viennot-Bourgin G (1952a) Urédinales d'Afrique (2° note). Urédinales de la Côte d'Ivoire (1° note). Bulletin de la Société Mycologique de France 67:429–435.

Viennot-Bourgin G (1952b) Ustilaginales d'Afrique (deuxième note). Etude critique du genre *Mycosyrinx*. Revue Internationale de Botanique Appliquée et d'Agriculture Tropicale 32:253–264. https://doi.org/10.3406/jatba.1952.6500.

Viennot-Bourgin G (1955) Urédinales d’Afrique 4. Urédinales de Côte d’Ivoire 3. Bulletin de la Société Mycologique de France 70:410–419.

Viennot-Bourgin G (1957) Trois Ustilaginales nouvelles de Guinée française. Bulletin de la Société Botanique de France 104:266–275. https://doi.org/10.1080/00378941.1957.10835108.

Viennot-Bourgin G (1958a) Ustilaginales nouvelles de Guinée française (deuxième note). Revue de Pathologie Végétale et d'Entomologie Agricole de France 37:167–178.

Viennot-Bourgin G (1958b) Urédinales d'Afrique (5e note). Urédinales de Côte d'Ivoire (4e note). Uredineana 5:137–248.

Viennot-Bourgin G (1959) Étude de micromycètes parasites récoltés en Guinée. Annales de l'Institut National Agronomique 45:1–91.

Vincent MA, Blackwell WH (1988) *Botrysporium hughesii,* a new species from Africa. Mycotaxon 32:467–470.

Voglmayr H, Piątek M (2008) *Peronospora* causing downy mildew disease of sweet basil newly reported in Cameroon. New Disease Reports 18:1–6.

Voglmayr H, Piątek M (2009) *Peronospora* causing downy mildew disease of sweet basil newly reported in Cameroon. Plant Pathology 58:805. https://doi.org/10.1111/j.1365-3059.2009.02047.x.

von Arx JA (1975) Revision of *Microascus* with the description of a new species. Persoonia 8:191–197.

von Arx JA, Hennebert GL (1965) Deux champignons ambrosia. Mycopathologie et Mycologia Applicata 25:309–315.

von Arx JA, Guarro J, van der Aa HA (1987) *Asordaria*, a new genus of the Sordariaceae, and a new species of *Melanocarpus*. Persoonia 13:263–272.

Wakefield AE, Stewart TJ, Moxon ER, Marsh K, Hopkin JM (1990) Infection with *Pneumocystis carinii* is prevalent in healthy Gambian children. Transactions of the Royal Society of Tropical Medicine and Hygiene 84:800–802. https://doi.org/10.1016/0035-9203(90)90087-U.

Wakefield EM (1912) Nigerian fungi. Bulletin of Miscellaneous Information 1912:141–144.

Wakefield EM (1914) Nigerian Fungi - II. Bulletin of Miscellaneous Information 1914:253–261.

Wakefield EM (1917) Nigerian fungi - III. Bulletin of Miscellaneous Information 1917:105–111.

Wakefield EM (1918a) A disease of the yam (*Bagnisiopsis dioscoreae*). Bulletin of Miscellaneous Information 6:199–201.

Wakefield EM (1918b) Fungi exotici - XXIV. Kew Bulletin 1918:207–210.

Wakefield EM, Hansford CG (1949) Contributions towards the fungus flora of Uganda. IX. The Uredinales of Uganda. Proceedings of the Linnean Society of London 161:162–198. https://doi.org/10.1111/j.1095-8312.1949.tb00565.x.

Walleyn R, Rammeloo J (1994) The poisonous and useful fungi of Africa south of the Sahara. Scripta Botanica Belgica 10:1–56.

Waterhouse GM, Brothers MP (1981) The taxonomy of *Pseudoperonospora*. Mycological Papers 148:1–28.

Watling R, Turnbull E (1992) Boletes from South and East Central Africa – I. Edinburgh Journal of Botany 49:343–361. https://doi.org/10.1017/S0960428600000585.

Wendt L, Sir EB, Kuhnert E, Heitkämper S, Lambert C, Hladki, AI, Romero AI, Luangsa-ard, JJ, Srikitikulchai P, Peršoh D, Stadler M (2017) Resurrection and emendation of the Hypoxylaceae, recognised from a multigene phylogeny of the Xylariales. Mycological Progress 48:1–40. https://doi.org/10.1007/s11557-017-1311-3.

West J (1938) A preliminary list of plant diseases in Nigeria. Bulletin of Miscellaneous Information 1938:17–23.

Whalley AJS, Hammelev D, Taligoola HK (1988) Two new species of *Hypoxylon* from Nigeria. Transactions of the British Mycological Society 90:139–141. https://doi.org/10.1016/S0007-1536(88)80195-5.

Wickerham L (1969) New homothallic taxa of *Hansenula*. Mycopathologie et Mycologia Applicata 37:15–32.

Wilson JP, Hess DE, Kumar KA (2000) *Dactuliophora* leaf spot of pearl millet in Niger and Mali. Plant Disease 84:201. https://doi.org/10.1094/PDIS.2000.84.2.201D.

Wolf FA (1949) Two unusual conidial fungi. Mycologia 41:561–564. https://doi.org/10.2307/3755076.

Woudenberg JHC, Groenewald JZ, Binder M, Crous PW (2013) *Alternaria* redefined. Studies in Mycology 75:171–212. https://doi.org/10.3114/sim0015.

Woudenberg JHC, Seidl MF, Groenewald JZ, Vries M de, Stielow JB, Thomma BPHJ, Crous PW (2015) *Alternaria* section *Alternaria*: Species, formae speciales or pathotypes? Studies in Mycology 82:1–21. https://doi.org/10.1016/j.simyco.2015.07.001.

Wu W, Sutton BC (1995) Additions to the genus *Xenidiocercus* (Coelomycetes) from Ghana. Mycoscience 36:271–275. https://doi.org/10.1007/BF02268601.

Wulff EG, Sørensen JL, Lübeck M, Nielsen KF, Thrane U, Torp J (2010) *Fusarium* spp. associated with rice Bakanae: Ecology, genetic diversity, pathogenicity and toxigenicity. Environmental Microbiology 12:649–657. https://doi.org/10.1111/j.1462-2920.2009.02105.x.

Yamamoto Y, Hagiwara H, Murano H, Sando H (1996) Several myxomycetes from Sierra Leone. Bulletin of the National Museum of Nature and Science, Ser. B 22:22–26.

Yaninek JS, Saizonou S, Onzo A, Zannou I, Gnanvossou D (1996) Seasonal and habitat variability in the fungal pathogens, *Neozygites* cf. *floridana* and *Hirsutella thompsonii*, associated with cassava mites in Benin, West Africa. Biocontrol Science and Technology 6:23–34. https://doi.org/10.1080/09583159650039502.

Yen J-M (1974) Les *Cercospora* de Côte d'Ivoire - I. Bulletin de la Société Mycologique de France 90:307–324.

Yen J-M (1975) Les *Cercospora* de Côte d'Ivoire - II. Bulletin de la Société Mycologique de France 91:89–103.

Yen J-M (1976a) Les Urédinées de Côte d'Ivoire - I. Revue de Mycologie 40:125–139.

Yen J-M (1976b) Les Urédinées de Côte d'Ivoire - II. Revue de Mycologie 40:283–294.

Yen J-M (1978) Les *Cercospora* de Côte d'Ivoire - III. Bulletin de la Société Mycologique de France 94:381–383.

Yorou NS (2010) Champignons: Champignons supérieurs. In: Konate S, Kampmann D (eds) Atlas de la biodiversité de l'Afrique de l'Ouest, Tome III: Côte d'Ivoire. Goethe-Universität Frankfurt am Main, Frankfurt, pp 324–335.

Yorou NS, Agerer R (2007) *Tomentella furcata*, a new species from Benin (West Africa) with basidia forming internal hyphae. Mycological Progress 6:239–247. https://doi.org/10.1007/s11557-007-0543-z.

Yorou NS, Agerer R (2008) *Tomentella africana*, a new species from Benin (West Africa) identified by morphological and molecular data. Mycologia 100:68–80. https://doi.org/10.3852/mycologia.100.1.68.

Yorou NS, de Kesel A (2002) Connaissances ethnomycologiques des peuples Nagot du centre du Bénin (Afrique de l'Ouest). Systematics and Geography of Plants 71:627–637.

Yorou NS, de Kesel A (2011) Champignons supérieurs: Larger fungi. In: Neuenschwander P, Sinsin B, Goergen G (eds) Protection de la nature en Afrique de l'Ouest: Une liste rouge pour le Bénin = Nature conservation in West Africa: Red List for Benin. International Institute of Tropical Agriculture (IITA), Ibadan, Oyo State, Nigeria, pp 47–60.

Yorou NS, de Kesel A, Sinsin B, Codjia JT (2001) Diversité et productivité des champignons comestibles de la forêt classée de Wari-Maro (Benin, Afrique de l'Ouest). Systematics and Geography of Plants 71:613–625. https://doi.org/10.2307/3668706.

Yorou NS, Kõljalg U, Sinsin B, Agerer R (2007) Studies in African thelephoroid fungi: 1. *Tomentella capitata* and *Tomentella brunneocystidia*, two new species from Benin (West Africa) with capitate cystidia. Mycological Progress 6:7–18. https://doi.org/10.1007/s11557-006-0519-4.

Yorou NS, Diabaté M, Agerer R (2011a) Phylogenetic placement and anatomical characterisation of two new West African *Tomentella* (Basidiomycota, Fungi) species. Mycological Progress 11:171–180. https://doi.org/10.1007/s11557-011-0739-0.

Yorou NS, Guelly AK, Agerer R (2011b) Anatomical and ITS rDNA-based phylogenetic identification of two new West African resupinate thelephoroid species. Mycoscience 52:363–375. https://doi.org/10.1007/S10267-011-0117-4.

Yorou NS, Diabaté M, Agerer R (2012a) Two new resupinate Thelephorales (Basidiomycota, Agaricomycetes) from Guinea (West Africa). Nova Hedwigia 96:167–180. https://doi.org/10.1127/0029-5035/2012/0056.

Yorou NS, Gardt S, Guissou M-L, Diabaté M, Agerer R (2012b) Three new *Tomentella* species from West Africa identified by anatomical and molecular data. Mycological Progress 11:449–462. https://doi.org/10.1007/s11557-011-0760-3.

Yorou NS, Koné NA, Guissou KML, Guelly AK, Maba DL, Ekue MR, de Kesel A (2014) Biodiversity and sustainable use of wild edible fungi in the Sudanian centre of endemism: a plea for valorization. In: Bâ AM (ed) Ectomycorrhizal symbioses in tropical and neotropical forests. CRC Press, Boca Raton, pp 241–269.

Zambettakis C (1951a) *Diplodia natalensis* Pole Evans stem-end rot des argrumes. Revue de Mycologie, Supplément Colonial 16:1–8.

Zambettakis C (1951b) Sur une sphaeropsidacée nouvelle parasite de *Rauwolfia vomitoria* Afz. Bulletin de la Société Mycologique de France 66:228–232.

Zambettakis C (1970) Recherches sur les Ustilaginales d'Afrique. Bulletin de la Société Mycologique de France 86:306–692.

Zambettakis C (1973) Recherches sur les charbons des Arundinelleae. Revue de Mycologie 38:67–90.

Zambettakis C (1977) *Ustilago paspalidicola* nov. sp. et sa position taxonomique. In: Société française de phytopathologie (ed) Travaux dédiés à Georges Viennot-Bourgin, pp 413–416.

Zare R, Gams W (2001) A revision of *Verticillium* section *Prostrata -* IV. The genera *Lecanicillium* and *Simplicillium* gen. nov. Nova Hedwigia 73:1–50.

Zhao R, Karunarathna SC, Raspé O, Parra LA, Guinberteau J, Moinard M, de Kesel A, Barroso G, Courtecuisse R, Hyde KD, Guelly AK, Desjardin DE, Callac P (2011) Major clades in tropical *Agaricus*. Fungal Diversity 51:279–296. https://doi.org/10.1007/s13225-011-0136-7.

Zida PE, Séréme P, Leth V, Sankara P, Somda I, Néya A (2008) Importance of seed-borne fungi of sorghum and pearl millet in Burkina Faso and their control using plant extracts. Pakistan Journal of Biological Science 11:321–331.

Zoberi MH (1972) Tropical macrofungi: Some common species. Macmillan, London.

Zoberi MH (1973) Some edible mushrooms from Nigeria. The Nigerian Field 38:81–90.

Zoberi MH (1979) Some edible mushrooms from the tropics. Mushroom Science 10:519–536.

Zucconi L (1991) *Pseudobeltrania guerensis* sp. nov. from Ivory Coast forest litter. Mycological Research 95:1017–1018.

Zucconi L (1992) *Hemibeltrania cymbiformis* sp. nov., a new hyphomycete from Ivory Coast forest litter. Mycological Research 96:145–146. https://doi.org/10.1016/S0953-7562(09)80930-1.

Zucconi L, Onofri S (1986) Two new dematiaceous hyphomycetes from tropical forest litter. Mycotaxon 27:147–153.

Zucconi L, Onofri S (1989) *Gyrothrix ramosa* sp. nov. and notes on *G. citricola*. Mycological Research 92:380–382. https://doi.org/10.1016/S0953-7562(89)80086-3.

Zucconi L, Rambelli A (1982) Una nuova specie di *Zanclospora* dalla lettiera del Parco Nazionale di Tai. Micologia Italiana 11:51–52.

Zucconi L, Rambelli A (1993) A new species of *Chaetopsina* from tropical forest litter. Mycotaxon 48:5–12.
