# Supplementary figures and images for: Mapping mycological ignorance – checklists and diversity patterns of fungi known for West Africa
Source: IMA Fungus. 2020 Jul 7;11:13. doi: 10.1186/s43008-020-00034-y (PMC7341642; doi:10.1186/s43008-020-00034-y)

**$R = 0.12, P = 0.14$**

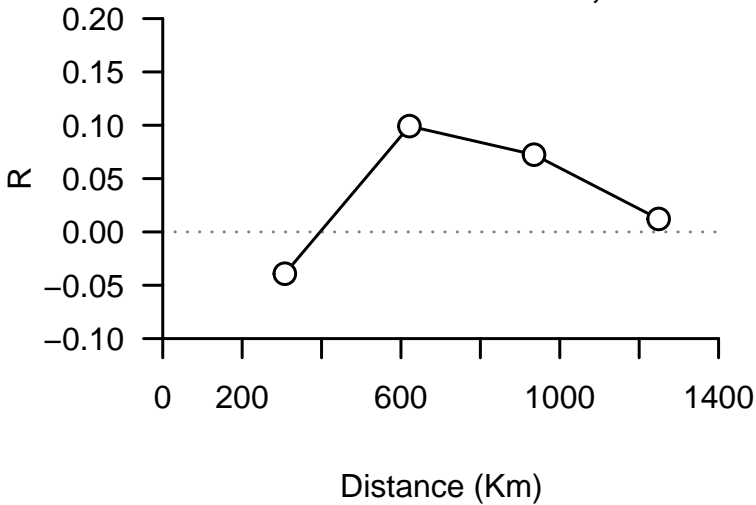

Supplement: Supplementary file 16 — Additional file 16. Mantel correlogram for knowledge of fungal species in West African countries. [file 43008_2020_34_MOESM16_ESM.pdf]
