# Supplementary material for: Mapping mycological ignorance – checklists and diversity patterns of fungi known for West Africa
Source: IMA Fungus. 2020 Jul 7;11:13. doi: 10.1186/s43008-020-00034-y (PMC7341642; doi:10.1186/s43008-020-00034-y)
Supplement: Supplementary file 17 — Additional file 17. Presentation and discussion of fungal species new to Benin. [file 43008_2020_34_MOESM17_ESM.docx]

**Additional file 17: New records of fungal species for Benin**

***Ascomycota***

***Asterinales***

# 1. *Asterina opiliae* Mibey

Fig. 4a

Specimen examined: On *Opilia celtidifolia* Endl. ex Walp. (Opiliaceae), det. N. S. Yorou. Benin: Department of Borgou, near Parakou, Okpara forest, N 9°15'32'' E 2°43'30'', alt. 350 m a.s.l., 5.8.2017, leg. M. Piepenbring, N.S. Yorou, and participants of the Summer School MP 5360, det. M. Piepenbring.

Colonies on the upper side of leaves, composed of loose hyphae with appressoria and thyriothecia. Appressoria opposite or alternate, each formed by one cell, elongate, (10–)11–14(–15) x 4–6 µm (n= 12). Thyriothecia circular, diam. (110–)120–150(–160) µm (n=14). Asci rarely seen, one ascus seen containing two ascospores. Ascospores two-celled, (21–)22–24(–25) x 10–12 µm (n=10), brown, wall covered by warts.

Known distribution: *Asterina opiliae* is only known from the type locality in Kenya (Mibey & Hawksworth 1997). It is reported here for the first time for West Africa.

Known host plants: *Asterina opiliae* is only known from the host species of the type, *Opilia* sp. It is reported here for the first time for *Opilia celtidifolia*.

The characteristics observed in the specimen from Benin correspond to those described for the specimen from Kenya except the size of the thyriothecia (Kenya: 185–270 µm diam. versus Benin: 120–160 µm) and the fact that Mibey and Hawksworth (1997) describe asci with eight ascospores each.

***Erysiphales***

# 2. *Leveillula clavata* Nour

Fig. 4b

Specimen examined: On *Euphorbia heterophylla* L. (Euphorbiaceae), det. M. Piepenbring. Benin: Department of Donga, Taneka, Hotel Taneka Coco, alt. 430 m a.s.l., N 9°52'27.06" E 1°30'49.13", 31.07.2017, leg. M. Piepenbring, N.S. Yorou, and participants of the Summer School B 16, det. M. Piepenbring.

Only asexual structures of *Ovulariopsis* type were found. Hyphae with unlobed appressoria. Conidia clavate to spathulate, developing singly. Size of conidia (47–)50–68(–80) x 12–18(–20) µm (n=10).

Known host plants: *Euphorbia acalyphoides*, *Euphorbia balsamifera*, *Euphorbia convolvuloides*, *Euphorbia pulcherrima* (Braun & Cooke 2012). This is the first record of this species on *Euphorbia heterophylla*.

Known distribution: *Leveillula clavata* is known from Africa (Canary Islands, Kenya, Senegal, Sudan, Tanzania) and Asia (India, Indonesia), and it was sporadically introduced to Europe (Italy, UK) (Braun & Cooke 2012). It is reported here for the first time for Benin.

According to Braun and Cooke (2012) conidia measure 45–65 x 14–18 µm, so they are slightly shorter than those measured from Benin. The shape of the conidia is similar.

# 3. *Pseudoidium azadirachtae* (Narayanas. & K. Ramakr.) U. Braun & R.T.A. Cook

Fig. 4c

Specimen examined: On *Azadirachta indica* A. Juss. (Meliaceae), det. M. Piepenbring. Benin: Department of Atlantique, Cotonou, Université d´Abomey Calavi, Botanical Garden, N 6°26'59" E 2°20'48", alt. ca. 15 m a.s.l., 10.08.2017, leg. M. Piepenbring MP 5377, det. M. Piepenbring.

Only asexual structures were found on both sides of the leaves, with more abundant fungal cells on the upper side than on the lower side of the leaves. Superficial hyphae with lobed appressoria. Conidia develop singly. Size of conidia (25–)27–37(–38) x 14–16(–18) µm (n=10).

Known distribution: *Pseudoidium azadirachtae* is known from India, Niger, and Pakistan (Braun & Cooke 2012). It is reported here for Benin for the first time.

According to Brown and Cooke (2012) conidia measure (22–)25–34.5 x 11–19 µm, so they are slightly shorter than those measured from Benin. The host plant species, the shape of the appressoria, and conidia developing singly on both sides of the leaves but more prominently on the upper side are characteristics showing the conspecificity.

***Meliolales***

# 4. *Meliola clerodendricola* Henn.

Fig. 4d

Specimen examined: On *Clerodendrum capitatum* (Willd.) Schumach. (Lamiaceae), det. M. Piepenbring. Benin: Department of Atlantique, north of Cotonou, close to Pahou, border of Pahou forest, N 6°22'56.17" E 2° 9'36.04", alt. 20 m a.s.l., 9.8.2017, leg. M. Piepenbring, N.S. Yorou, and participants of the Summer School MP 5371, det. M. Piepenbring & M. Bermúdez.

Colonies dense, epiphyllous. Spore-producing bodies perithecia, spherical, black, diam. (100–)124–168(–175) µm (n=10). Ascospores with 4 septa each, constricted at the septa, 33–38 x 12–14 µm (n=10). Mycelial setae straight or slightly bent, mostly simple at the tip, some clavate or with irregular shape, rarely with one short ramification and a septum, (205–)220–260(–280) x 6–7 µm (n=10). Capitate hyphae alternate, with stem cell (5–)6–10(–12) x 6–7.5(–8) µm (n=7) and appressorial cell globose to slightly pointed, 13–16 x 11–13 µm (n=7). Mucronate hyphae (10–)12–20(–22) x 6–8 µm (n=10).

Known distribution: *Meliola clerodendricola* is known from many tropical and subtropical countries all over the world including several countries in West Africa (Farr & Rossman 2018). It is reported here for the first time for Benin.

The characteristics observed for the specimen from Benin correspond to those described for this species by Hansford (1961) except for the size of the ascospores that are somewhat shorter (29–35 µm) than those from Benin.

***Xylariales***

# 5. *Podosordaria ustorum* (Pat.) P.M.D. Martin

Figs. 4e-f

Specimens examined: On burnt tussock of *Andropogon tectorum* Schumach. & Thonn. (Poaceae), det. M. Piepenbring. Benin: Department of Donga, Taneka, close to Hotel Taneka Coco, alt. 440 m a.s.l., N 9°52'27" E 1°30'49", 25.7.2017, leg. M. Piepenbring, N.S. Yorou, and participants of the Summer School, det. M. Piepenbring (too young, photos but no specimen). On burnt tussock of *Andropogon gayanus* Kunth (Poaceae), det. B. Sinsin & M. Piepenbring. Benin: Department of Donga, Kota waterfalls, N 10°12'42" E 1°26'49", alt. ca. 500 m a.s.l., 26.7.2017, leg. M. Piepenbring, K. Reschke, N.S. Yorou, and participants of the Summer School B 9, det. M. Piepenbring & H. Milenge.

Spore-producing bodies with terminal disc of mostly 3–6(–7) mm diam. (n=10), with a stipe 3–7 mm long and 1–1.5 mm diam. (n=10). Tips of perithecia evident as black dots in the white stroma of the disc, perithecia (120–)150–220 µm diam. (n=10). In longitudinal section, perithecia of approx. 400 µm diam. Asci with amyloid tip, wall of empty asci pointed. Ascospores eight in each ascus, uniseriate, ellipsoidal, 8–9 x 4–5 µm (n=10), with two oil drops each.

Known distribution: *Podosordaria ustorum* is known from New Caledonia (Patouillard 1887), Nigeria (Wakefield 1912) and Venezuela (Patouillard 1887). It is reported here for Benin for the first time.

The characteristics provided by Patouillard (1887) correspond to those observed for the specimen from Benin.

***Basidiomycota***

***Agaricales***

# 6. *Chlorophyllum globosum* (Mossebo) Vellinga

Fig. 4g

Specimen examined: On soil. Benin: Department of Borgou, Parakou, Centre Guy Riobe, N 9°20'34.5" E 2°38'20.7", 380 m a.s.l., 29.07.2016, leg. K. Reschke, N.S. Yorou, and participants of the Summer School KaiR111, det. K. Reschke, ITS sequence MT110230.

Spore-producing bodies agaricoid, pileus up to about 15 cm diam., white with brownish squamules. Stipe with double ring. Spore powder greenish.

Basidiospores thick-walled, amygdaloid, truncate, with germ porus, 10–12 x 7.5–8.5 µm (n = 15). Lamellae edge sterile, cheilocystidia broadly clavate to sphaeropedunculate, slightly brownish pigmented, about 32–41 x 12–21 µm. The ITS sequence is 100 % identical to sequences of specimens identified by Ge *et al.* (2018).

Known distribution: *Chlorophyllum globosum* is known from Cameroon, Nigeria, South Africa, China, India, and Thailand (Ge *et al.* 2018). It is reported here for Benin for the first time. Specimens reported for West African countries as *Chlorophyllum molybdites* (G. Mey.) Massee probably belong to the species *C. globosum* or *C. palaeotropicum* Z.W. Ge & A. Jacobs, because *C. molybdites* apparently does not occur in Africa.

# 7. *Gymnopus gibbosus* (Corner) A.W. Wilson, Desjardin & E. Horak

Fig. 4h

Specimen examined: On dead stem of *Detarium microcarpum*. Benin: Kota waterfalls, N 10°12'40.8'' E001°26'50.9'', 480 m a.s.l., 01.08.2016, leg. K. Reschke, N.S. Yorou, and participants of the Summer School KaiR72, det. K. Reschke, ITS sequence MT110227.

Spore-producing bodies agaricoid. Pileus 1.2–2.3 cm diam., brownish cream, smooth, innately fibrillose, translucently striate to about one third of the radius. Basidia 4-spored. Basidiospores oblong to slightly dacryoid, 6–9 x 3–4 µm (n = 11). Cheilocystidia scattered single or in clusters between basidia, irregularely lageniform, some capitate, few branched. Clamp connections abundant. The ITS sequence is identical in 671 of 673 nucleotides to the sequence of a specimen identified by Desjardin and Perry (2017).

Known distribution: *Gymnopus gibbosus* is known from São Tomé, Indonesia (Java), and Singapore (Desjardin & Perry 2017). It is reported here for the first time from the West African mainland.

# 8. *Hohenbuehelia* aff. *grisea* (Peck) Singer

Fig. 4i

Specimen examined: On bark of log, tree not identified. Benin: Department of Atlantique, north of Allada, Niaouli, N 06°44'44.9'' E 002°07'43.0'', 60 m a.s.l., 08.08.2017, leg. K. Reschke, N.S. Yorou, and participants of the Summer School KaiR770, det. K. Reschke, ITS sequence MT110235.

Spore-producing bodies spathulate, without stipe, up to 0.5 cm diam. Pileus greyish. Lamellae edge sterile, cheilocystidia lageniform with capitulum. Pleurocystidia metuloid, wall up to 6.5 µm thick, acute obclavate, with cristal cap. Basidia 4-spored. Few mature basidiospores found, 7–7.5 x 3.5–4 µm (n = 3).

Known distribution: Members of the *Hohenbuehelia grisea* species complex are widespread, being documented in scientific literature for Europe, North and Central America, as well as East Asia (e.g. Consiglio *et al.* 2018). This species name has not yet been cited for any country in Africa, but the species may have been reported for Africa and even West Africa (Ghana) by another name (see below).

In the past, the species concept of *Hohenbuehelia grisea* was broad and covered specimens from diverse countries all over the world. Since the detailed study presented by Consiglio *et al.* (2018), we know that several species have been referred to by this name. The present specimen may represent *Hohenbuehelia robusta* (F.R. Jones) Consiglio, Setti & Thorn, that was described from Ghana (Jones 1964). However, this species is up to now only known in its asexual form *Nematoctonus robustus* F.R. Jones, so there are no morphological characteristics available for comparison, and there are no sequence data from the type of this species.

The ITS sequence from the specimen from Benin is 100 % identical to the sequence of a specimen annotated as *Hohenbuehelia* grisea s.l. from Thailand (GenBank accession MF150036). Sequences of *H. grisea* s.str., however, differ by approximately 4 % of the base pairs.

# 9. *Lycoperdon endotephrum* Pat.

Fig. 4j

Specimen examined: On soil in peanut field. Benin: Department of Donga, Taneka, N 9°52'34.1"E 1°30'40.2", 430 m a.s.l., 02.08.2016, leg. K. Reschke, M. Piepenbring, N.S. Yorou, and participants of the Summer School KaiR86, det. K. Reschke, ITS sequence MT110228.

Spore-producing bodies solitary or in pairs, gasteroid, globose, 0.7-2 cm diam., white to yellowish, older spore-producing bodies greyish-brown. Exoperidial spines joined at tips, concolorous. Gleba white in immature spore-producing bodies, separated from the subgleba by an inconspicuous, umbonate diaphragma. Mature spore-producing bodies with ostiole at the tip and brown, powdery gleba. Spore powder brown. Basidiospores globose, verruculose, (3-)3.5-4 µm diam. (n = 15). Paracapillitium and capillitium present.

Known distribution: *Lycoperdon endotephrum* is known for Madagascar (Patouillard 1902), the Democratic Republic of Congo, and Ruanda (Demoulin & Dring 1975). It is reported here for the first time for Benin and West Africa.

The ITS sequence of this specimen is similar to sequences annotated as *Lycoperdon curtisii* Berk. and *Lycoperdon pratense* Pers., with p-distances of about 1.5 %. This close relationship is also evident by morphological characteristics as noticed by Patouillard (1902) and Demoulin and Dring (1975). *Lycoperdon endotephrum* is not represented by sequence data in GenBank up to now, so the present identification is mainly based on morphological characteristics.

# 10. *Panaeolus bisporus* (Malençon & Bertault) Ew. Gerhardt

Fig. 5a

Specimen examined: On soil. Benin: Department of Borgou, close to the village Wari Maro, N 09°08'36.7'' E002°07'59.7'', 410 m a.s.l., 05.08.2016, K. Reschke, N.S. Yorou, and participants of the Summer School KaiR95, det. K. Reschke, ITS sequence MT110229.

Spore-producing bodies agaricoid. Pileus 1–1.2 cm diam., whitish in the analyzed collection, but maybe hygrophanous. Stipe 3–3.8 cm long. Spore powder blackish brown. Basidia 2-spored. Cheilocystidia thin-walled, hyaline, lageniform, about 15–25 x 5–10 µm. Pleurocystidia thick-walled, with brownish pigment, about 45–54 x 13–17 µm. Basidiospores thick-walled, citriform to amygdaloid, with dark brown pigment and about 2 µm wide germ porus, 12.5–13.5 x 8–10 µm in side view (n = 11).

Known distribution: *Panaeolus bisporus* is known for Morocco, Spain, and Switzerland (Gerhardt 1996, Senn-Irlet *et al.* 1999). It is reported here for the first time for West Africa.

# 11. *Tetrapyrgos atrocyanea* (Métrod) E. Horak

Fig. 5b

Specimen examined: On sticks of 6-8 mm diam. of unknown tree. Benin: Department of Atlantique, north of Cotonou, close to Pahou, Pahou forest, N 06°22'56.8'' E 002°09'36.6'', 20 m a.s.l., leg. K. Reschke, N.S. Yorou, and participants of the Summer School KaiR783, det. K. Reschke, ITS-sequence MT110236.

Spore-producing bodies agaricoid. Pileus up to 1.8 cm diam., cream to olivaceous brown, pruinose. Stipe black with white pruina. Lamellae edge sterile, cheilocystidia 36–48 µm long, diverticulate. Basidiospores tetrahedral, 7–8 x 6.5–7.5 µm (n = 10). The ITS sequence is identical in 665 of 669 nucleotides to the ITS-sequence of the epitype (MF075137).

Known distribution: *Tetrapyrgos atrocyanea* is known from Madagascar (Métrod 1949), Argentina, Bolivia, Brazil, British Virgin Islands, Costa Rica, and Puerto Rico (Honan *et al.* 2015). It is reported here for the first time for the African continent.

According to descriptions in literature, blueish tinges develop in old spore-producing bodies, but they were not observed on pilei of the analyzed specimen. Desjardin *et al.* (2017) showed that *Tetrapyrgos longicystidiata* is a synonym of *T. atrocyanea*. According to Honan *et al.* (2015) the cheilocystidia of *T. longicystidiata* are mostly longer than 50 µm. All analyzed cheilocystidia of the examined specimen, however, were smaller than 50 µm.

***Boletales***

# 12. *Scleroderma dictyosporum* Pat.

Fig. 5c

Specimen examined: On soil. Benin: Department of Donga, Kota waterfalls, N 10°12'41.62" E 1°26'46.50", alt. 500 m a.s.l., 26.7.2017, leg. M. Piepenbring, N.S. Yorou, and participants of the Summer School B 06, det. M. Piepenbring.

Spore-producing bodies globose, mostly (8–)10–12 mm diam. (n=10), attached to the soil by a short stipe with dense hyphae. Peridia smooth, with very shallow brownish warts. Gleba ochre coloured when mature, composed of basidiospores and sterile hyphae (capillitium). Capillitium strands mostly 3–4 µm diam., no septa observed, branched, smooth, not pigmented. Basidiospores spherical to globose, 7–9 x (6–)7–8 µm (measured without reticulum), covered by coarse reticulum 2–3 µm high.

Known distribution: *Scleroderma dictyosporum* is known from several tropical countries all around the world including several countries in West Africa (Guzmán 1970). It is reported here for Benin for the first time.

According to Guzmán (1970) spore-producing bodies can be larger, up to 40 mm.

***Doassansiales***

# 13. *Rhamphospora nymphaeae* D.D. Cunn.

Fig. 5d

Specimen examined: On *Nymphaea* sp. (Nymphaeaceae). Benin: Department of Zou, close to Kouzounkpa, Lokoli, swamp forest, N 7° 3'41.08" E 2°15'32.76", 80 m a.s.l., 11.08.2017, leg. M. Piepenbring, B. Sinsin, N.S. Yorou, and participants of the Summer School 5382, det. M. Piepenbring & S. Mullings Dreer.

Infection evident by yellowish spots on leaves. Teliospores intercellular in leaf tissue, lemon shaped, 15–21(–25) x 11–15(–19) µm (n=20).

Known distribution: *Rhamphospora nymphaeae* is a cosmopolitan species according to Vánky (2012). It is reported here for West Africa for the first time and may also be new to Africa, as no record of *R. nymphaeae* could be found for any African country.

According to Vánky (2012) teliospores measure 8–14 x 7–11 µm, so teliospores from Benin are larger. As all the other characteristics of the specimen from Benin are similar to characteristics described by Vánky (2012), we apply the name *R. nymphaeae* in spite of this difference.

***Exobasidiales***

# 14. *Graphiola phoenicis* (Moug. ex Fr.) Poit.

Specimen examined: On *Phoenix* sp. (Arecaceae), det. M. Piepenbring. Benin: Department of Atlantique, Cotonou, campus of the Université d´Abomey Calavi, in front of the Botany building, N 6°26'59" E 2°20'48", alt. ca. 15 m a.s.l., 23.7.2017, leg. M. Piepenbring, N.S. Yorou, and participants of the Summer School B 7, det. M. Piepenbring & S. Mullings Dreer.

Spore-producing bodies pot shaped, black, often fused and peridia of irregular shape, spore-producing bodies (0.5–)0.6–0.9(–1.0) mm diam. (n=10) and (0.2–)0.3–0.4 mm high (n=10). Primary spores (basidiospores) attached to basidial cells in dispersed positions. Size of the secondary (part) spores 2–3.5(–4) x 2–3 µm (n=10).

Known distribution: *Graphiola phoenicis* is known from many tropical and subtropical countries all over the world including countries of West Africa (Piepenbring *et al.* 2012). It is reported here for the first time for Benin.

According to Piepenbring *et al.* (2012) spore-producing bodies of *G. phoenicis* measure (0.3–)0.4–0.6(–0.7) mm in diameter, so they are smaller than those measured from Benin. The height of the spore-producing bodies and the size of the secondary spores are similar.

***Lepidostromatales***

# 15. *Sulzbacheromyces miomboensis* De Kesel & Ertz

Fig. 5e

Specimens examined: On bare soil. Benin: Department of Donga, Taneka, close to Hotel Taneka Coco, N 09°52'36.2'' E001°30'59.1'', 430 m a.s.l., grassland with scattered trees, 25.07.2017, leg. K. Reschke, M. Piepenbring, N.S. Yorou, and participants of the Summer School, KaiR 714, det. K. Reschke, ITS sequence MT110232; On bare soil. Benin: Department of Atakora, near Natitingou, Kota waterfalls, N 10°12'43.2'' E001°26'46.9'', 500 m a.s.l., open forest, 26.07.2017, leg. K. Reschke, M. Piepenbring, N.S. Yorou, and participants of the Summer School KaiR 720, det. K. Reschke, ITS sequence MT110233; On bare soil and the surface of a termite mound. Benin: Department of Donga, Taneka, agricultural land around Taneka, N 09°52'16.1'' E001°30'06.1'', 430 m a.s.l., 29.07.2019, leg. K. Reschke, M. Piepenbring, N.S. Yorou, and participants of the Summer School KaiR 733, det. K. Reschke, ITS sequence MT110234.

Spore-producing bodies clavarioid, 1.5–3.8 x 0.15–0.3 cm, orange. Basidiospores oblong to slightly phaseoliform, (8.5–)9–12(–12.5) x (3.5–)4–5 µm (n = 37 spores of 3 collections). Clamp connections absent. ITS sequences are identical in 539 of 543 nucleotides to the ITS sequence of the holotype (NR_159036).

Known distribution: *Sulzbacheromyces miomboensis* has been described recently from the Democratic Republic of the Congo (Liu *et al.* 2017). It is reported here for the first time for West Africa. This is the first basidiolichen being reported for West Africa.

***Phallales***

# 16. *Phallus aurantiacus* Mont.

Fig. 5f

Specimen examined: On soil. Benin: Department of Borgou, close to Parakou, Songhai Atagara project site, N 9°24'35" E 2°41'32", alt. ca. 340 m a.s.l., 2.8.2017, leg. M. Piepenbring, N.S. Yorou, and participants of the Summer School B 17 (specimen lost, only photo available), det. M. Piepenbring, J. M. Tchotet Tchoum & G. Konomou.

Size of the spore-producing bodies 6–7.8 x 1.6–1.8 cm (n=2). Basidiospores cylindrical, 4–6 x 2–3 µm, hyaline, smooth.

Known distribution: *Phallus aurantiacus* is known from several countries in West Africa (Dring & Rose 1977). It is reported here for Benin for the first time.

According to Dring and Rose (1997) basidiospores of *P. aurantiacus* measure 3.5–4 x 2–2.5 µm. The basidiospores from Benin are slightly longer than those described by Dring and Rose (1997).

***Polyporales***

# 17. Nigroporus stipitatus Douanla-Meli & Ryvarden

Fig. 5g

Specimen examined: On burried, decayed wood. Benin: Department of Plateau, close to Pobé, Pobé forest, N 6°57'47.5"E 2°40'13.1", 120 m a.s.l., 13.08.2016, leg. K. Reschke, M. Piepenbring, N.S. Yorou, and participants of the Summer School KaiR116, ITS sequence MT110231, det. K. Reschke.

Spore-producing bodies polyporoid, tough, thin-fleshed. Pileus spathulate to infundibuliform, 3–7 cm diam., dark violet, violet brown to black, concentrically zonate. Stipe central to excentric, 1.5–2 x 0.5–0.8 cm, dark violet to black. Pore layer violet-brown to grey with violet tinge. Pores very small, almost not visible without lense. Trama and pores blackening in 5 % KOH. Basidiospores not seen.

Known distribution: *Nigroporus stipitatus* has been described from Cameroon (Douanla-Meli *et al.* 2007). It is reported here for the first time for Benin and for West Africa.

The morphology of the specimen from Benin perfectly matches the data provided by Douanla-Meli *et al.* (2007). The ITS sequence obtained for the specimen from Benin is identical in 274 of 281 overlapping nucleotides to the partial ITS sequence of the isotype (JN710574). However, the ITS sequence of the isotype includes three ambiguous sites and the isotype sequence in the overlapping region is apparently of rather poor quality.

***Pucciniales***

# 18. *Aecidium flavidum* Berk. & Broome

Fig. 5h

Specimen examined: On *Pavetta crassipes* K. Schum. (Rubiaceae), det. N.S. Yorou. Benin: Department of Donga, close to Djougou, Bellefoungou forest, N 9°47'33'' E 1°42'40'', alt. 420 m a.s.l., 27.07.2017, leg. M. Piepenbring, N.S. Yorou, and participants of the Summer School B 11, det. M. Piepenbring & S. Mullings Dreer.

Spermatogonia in groups in the central area of circular leaf spots on the upper surface of the leaf and less numerous on the lower surface of the leaf surrounded by aecidia, lenticular, subcuticular (group VI, type 7, according to Cummins & Hiratsuka 2003), approx. 100–120 µm in diameter and 30–55 µm high.

Aecidia on lower side of leaves, with peridia. Peridial cells in front view (26–)27–32(–34) x (14–)15–19(–21) µm (n=10), with outer walls approx. 4–5 µm and inner walls approx. 2–3 µm thick. Size of aecidiospores (18–)20–23(–24) x (15–)16–19(–21) µm (n=20).

Known distribution: *Aecidium flavidum* was described very briefly on *Pavetta indica* from Sri Lanka (Berkeley & Broome 1873) and is known from several countries in Africa, like Guinea (Viennot-Bourgin 1959), Sierra Leone (Deighton 1936a, 1936b), and Uganda (Wakefield & Hansford 1948–49), as well as from the Philippines (Arthur & Cummins 1936) and New Guinea (Cummins 1941). It is reported here for the first time for Benin.

Known host plants: *Aecidium flavidum* is known from four species of *Pavetta* (Farr & Rossman 2018). *P. crassipes* is reported here for the first time as host species for this rust.

The size of the aecidiospores from Benin is similar to sizes of spores of *Aecidium flavidum* (17–23 x 16–19 µm) and *Aecidium pienaarii* (16–24 x 15–17 µm), as described by Viennot-Bourgin (1959). The size of the peridial cells from Benin is more similar to the size of the peridial cells described for *A. flavidum* (24–35 x 16–21 µm) than to *A. pienaarii* (22–30 x 16–22 µm). The size of the peridial cells, however, is difficult to measure and the difference is not clear.

Spermatogonia are described here for the first time for this species.

***Tilletiales***

# 19. *Conidiosporomyces ayresii* (Berk.) Vánky & R. Bauer

Specimen examined: On *Panicum maximum* Jacq. (Poaceae), det. M. Piepenbring. Benin: Department of Plateau, close to the village Pobé, Estation de Recherche sur le Palmier à l´Huile, border of forest, N 6°57'38" E 2°40'08", alt. 100 m a.s.l., 13.8.2016, leg. M. Piepenbring, N.S. Yorou, and participants of the Summer School 5321, det. M. Piepenbring & S. Mullings Dreer.

Sori corresponding to galls formed by ovaries of individual spikelets, sac-shaped, filled with teliospores and sterile cells. Teliospores spherical, 12–15(–16) x (10–)11–15 µm (n=10), brown, warty.

According to Vánky (2012), teliospores of *Conidiosporomyces ayresii* measure 13–17 x 12–16 µm.

Known distribution: *Conidiosporomyces ayresii* is known from many tropical countries all over the world (Vánky 2012). It is reported here for the first time for Benin.

***Oomycota***

***Albuginales***

# 20. *Albugo ipomoeae-panduratae* (Schwein.) Swingle

Figs. 5i, j

Specimen examined: On *Merremia aegyptia* (L.) Urb. (Convolvulaceae), det. M. Piepenbring. Benin: Department of Borgou, close to the village Wari Maro, at the base of the inselberg Soubakperou, N 9°08'19'' E 2°09'42'', alt. 430 m a.s.l., 3.8.2017, leg. M. Piepenbring, N.S. Yorou, and participants of the Summer School B 22, det. M. Piepenbring.

Sori hypophyllous, mostly 0.5–1 mm diam. Spores (sporangia) mostly bluntly cylindrical, some globose, (17–)19–22 x (14–)15–17(–18) µm (n=10), not pigmented, walls approx. 1 µm thick, smooth.

Known distribution: *Albugo ipomoeae-panduratae* is known from tropical and subtropical regions all over the world (Farr & Rossman 2018). It is reported here for the first time for Benin.

According to Viégas and Teixeira (1943), the spores of this species are somewhat smaller (14–18 x 13–14 µm). Other characteristics coincide.

# References for the taxonomic supplement

Arthur JC, Cummins GB (1936) Philippine rusts in the Clemens collection 1923-1926. Philippine Journal of Science, C. Botany 61:463-488 + plates.

Berkeley MJ, Broome CE (1873) Enumeration of the fungi of Ceylon. Part II, containing the remainder of the hymenomycetes, with the remaining established tribes of Fungi. (Continued). Journal of the Linnean Society of London, Botany 14:29-140.

Braun U, Cook RTA (2012) Taxonomic manual of the Erysiphales (powdery mildews). CBS biodiversity series, vol 11. CBS-KNAW Fungal Biodiversity Centre, Utrecht.

Consiglio G, Setti L, Thorn RG (2018) New species of *Hohenbuehelia*, with comments on the *Hohenbuehelia atrocoerulea* - *Nematoctonus robustus* species complex. Persoonia 41:202–212. https://doi.org/10.3767/persoonia.2018.41.10.

Cummins GB (1941) Uredinales of New Guinea. III. Mycologia 33:143–154. https://doi.org/10.2307/3754926.

Cummins GB, Hiratsuka Y (2003) Illustrated genera of rust fungi, 3rd edn., St. Paul.

Deighton FC (1936a) Preliminary list of fungi and diseases of plants in Sierra Leone. Bulletin of Miscellaneous Information 1936:397–424. https://doi.org/10.2307/4111838.

Deighton FC (1936b) List of fungi collected in Sierra Leone. Bulletin of Miscellaneous Information 7:424–433.

Demoulin V, Dring DM (1975) Gasteromycetes of Kivu (Zaire), Rwanda and Burundi. Bulletin du Jardin Botanique National de Belgique / Bulletin van de National Plantentuin van België 45:339–372.

Desjardin DE, Perry BA (2017) The gymnopoid fungi (Basidiomycota, Agaricales) from the Republic of São Tomé and Príncipe, West Africa. Mycosphere 8:1317–1391. https://doi.org/10.5943/mycosphere/8/9/5.

Desjardin DE, Perry BA, Shay E, Newman DS, Randrianjohany E (2017) The type species of *Tetrapyrgos* and *Campanella* (Basidiomycota, Agaricales) are redescribed and epitypified. Mycosphere 8:977–985. https://doi.org/10.5943/mycosphere/8/8/1.

Douanla-Meli C, Ryvarden L, Langer E (2007) Studies of tropical African pore fungi (Basidiomycota, Aphyllophorales): three new species from Cameroon. Nova Hedwigia 84:409–420. https://doi.org/10.1127/0029-5035/2007/0084-0409.

Dring DM, Rose AC (1977) Additions to West African phalloid fungi. Kew Bulletin 31:741–751.

Farr DF, Rossman AY (2018) Fungal databases, U.S. National Fungus Collections, ARS, USDA. https://nt.ars-grin.gov/fungaldatabases/. Accessed 26 Jul 2019

Ge Z-W, Jacobs A, Vellinga EC, Sysouphanthong P, van der Walt R, Lavorato C, An Y-F, Yang ZL (2018) A multi-gene phylogeny of *Chlorophyllum* (Agaricaceae, Basidiomycota): New species, new combination and infrageneric classification. MycoKeys 32:65–90. https://doi.org/10.3897/mycokeys.32.23831.

Gerhardt E (1996) Taxonomische Revision der Gattungen *Panaeolus* und *Panaeolina* (Fungi, Agaricales, Coprinaceae). Berlin, Freie Univ., Diss. Bibliotheca Botanica, vol 147. Schweizerbart, Stuttgart.

Guzmán G (1970) Monografía del género *Scleroderma* Pers. emend. Fr. Darwiniana 16:233–407.

Hansford CG (1961) The Meliolineae - A monograph. Beihefte zur Sydowia, Annales Mycologici, Ser. II., vol 2. Verlag von Ferdinand Berger, Horn.

Honan AH, Desjardin DE, Perry BA, Horak E, Baroni T (2015) Towards a better understanding of *Tetrapyrgos* (Basidiomycota, Agaricales): New species, type studies, and phylogenetic inferences. Phytotaxa 231:101–132.

Jones FR (1964) *Nematoctonus robustus* sp. nov. Transactions of the British Mycological Society 47:57–60. https://doi.org/10.1016/S0007-1536(64)80080-2.

Liu D, Goffinet B, Ertz D, de Kesel A, Wang X, Hur J-S, Shi H, Zhang Y, Yang M, Wang L (2017) Circumscription and phylogeny of the Lepidostromatales (lichenized Basidiomycota) following discovery of new species from China and Africa. Mycologia 109:730–748. https://doi.org/10.1080/00275514.2017.1406767.

Métrod G (1949) Les Mycènes de Madagascar. Prodome à une flore mycologique de Madagascar 3:1–146.

Mibey RK, Hawksworth DL (1997) Meliolaceae and Asterinaceae of the Shimba Hills, Kenya. Mycological Papers, vol 174. CAB International, Wallingford.

Patouillard N (1887) Champignons de la Nouvelle-Calédonie. Bulletin de la Société Mycologique de France 3:168–178, plate 17.

Patouillard N (1902) Descriptions de quelques champignons extra-européens. Bulletin de la Société Mycologique de France 18:300–304, 1 plate. (n.v.)

Piepenbring M, Nold F, Trampe T, Kirschner R (2012) Revision of the genus *Graphiola* (Exobasidiales, Basidiomycota). Nova Hedwigia 94:67–96. https://doi.org/10.1127/0029-5035/2012/0094-0067.

Senn-Irlet B, Nyffenegger A, Brenneisen R (1999) *Panaeolus bisporus* — an adventitious fungus in central Europe, rich in psilocin. Mycologist 13:176–179. https://doi.org/10.1016/S0269-915X(99)80107-4.

Vánky K (2012) Smut fungi of the world. APS Press, St. Paul.

Viégas AP, Teixeira AR (1943) Alguns fungos do Brasil (Phycomycetos). Bragantia 3:223–269.

Viennot-Bourgin G (1959) Étude de micromycètes parasites récoltés en Guinée. Annales de l'Institut National Agronomique 45:1–91.

Wakefield EM (1912) Nigerian fungi. Bulletin of Miscellaneous Information 1912:141–144.

Wakefield EM, Hansford CG (1949) Contributions towards the fungus flora of Uganda. IX. The Uredinales of Uganda. Proceedings of the Linnean Society of London 161:162–198. https://doi.org/10.1111/j.1095-8312.1949.tb00565.x.
